# Nr2f1a and Isl1 repress acquisition of epicardial identity in venous atrial cardiomyocytes

**DOI:** 10.1242/dev.205396

**Published:** 2026-06-04

**Authors:** Kendall E. Martin, Andrew T. Fernandes, Mohammed Sayed, Margaret A. Hanlon, Samuel Fallon, Hee-Woong Lim, Joshua S. Waxman

**Affiliations:** ^1^ Molecular Genetics, Biochemistry, and Microbiology Graduate Program, University of Cincinnati College of Medicine, Cincinnati, OH 45267, USA; ^2^ Molecular Cardiovascular Biology Division and Heart Institute, Cincinnati Children's Hospital Medical Center, Cincinnati, OH 45229, USA; ^3^ Molecular and Developmental Biology Graduate Program, University of Cincinnati College of Medicine, Cincinnati, OH 45229, USA; ^4^ Division of Biomedical Informatics, Cincinnati Children's Hospital Medical Center, Cincinnati, OH 45229, USA; ^5^ Department of Pediatrics, University of Cincinnati College of Medicine, Cincinnati, OH 45229, USA

**Keywords:** Heart development, Zebrafish, Atrial cardiomyocytes, Epicardial cells, Nr2f transcription factors, Isl1 transcription factor

## Abstract

Regulatory networks that maintain cardiomyocyte identity are not completely understood. Here, we have examined the relationship of the transcription factors Nr2f1a and Isl1, which, respectively, repress and promote pacemaker cardiomyocyte (PC) differentiation within the venous pole of zebrafish atria. Using zebrafish *nr2f1a*;*isl1* mutants, we found that loss of Isl1 exacerbates the inability to maintain the atrial cardiomyocyte (AC) identity found in *nr2f1a* mutant hearts. Subsequently, while *nr2f1a*;*isl1* mutants have a failure of PC differentiation and their ACs are unable to transdifferentiate into PC identity, ACs in *nr2f1a*;*isl1* mutant hearts lose myocardial marker expression, gain epicardial cell (EC) gene expression and exit the myocardial layer. Single-cell RNA-sequencing analysis of hearts supports the observation that the ACs and ECs of *nr2f1a*;*isl1* mutant embryos share common myocardial and EC transcriptomic signatures. Depletion of *tcf21* and *tbx18* in *nr2f1a*;*isl1* mutants was sufficient to prevent the acquisition of EC identity within putatively transdifferentiating ACs. Thus, our results reveal reiterative requirements for Nr2f and Isl1 transcription factors in binary fate decisions within the genetic hierarchy that maintains distinct AC, PC and EC identities at the venous pole of vertebrate hearts.

## Teaser

**Summary:** Nr2f and Isl1 transcription factors function concurrently within a regulatory network to repress epicardial cell identity and maintain venous atrial cardiomyocyte identity within zebrafish hearts.

## INTRODUCTION

Construction of the vertebrate heart involves the generation, incorporation and maintenance of distinct cardiac cell types (Bootman et al., 2006; Brandenburg et al., 2016; Keith and Flack, 1907; Marques et al., 2022; Moorman and Christoffels, 2003; Ng et al., 2010). Cardiomyocyte and non-cardiomyocyte populations of the heart originate from later-differentiating second heart field progenitor populations at the arterial and venous poles (Colombo et al., 2018; Knight and Yelon, 2016; Lazic and Scott, 2011; Rydeen and Waxman, 2016). Regulatory networks deployed within developmental windows have been shown to drive differentiation of the different cardiac cell types (myocardial, epicardial and endocardial), as well as establish cardiomyocyte diversity within the myocardium from these regions of greater potential that initially share gene expression (Harris and Black, 2010; Hu et al., 2000; Peralta et al., 2014; Sedmera et al., 2000; Song et al., 2019; Witzel et al., 2017; Zhou et al., 2011). Subsequently, interactions between key signaling pathways mediated via transcription factors (TFs) within these regulatory networks, many of which are used reiteratively, also maintain the identity of atrial, ventricular, atrioventricular canal (AVC) and pacemaker cardiomyocytes within the embryonic vertebrate heart (Arrenberg et al., 2010; Barth et al., 2005; Bloomekatz et al., 2016; Burkhard et al., 2017; Christoffels et al., 2010; Martin et al., 2023; Moorman and Christoffels, 2003; Ng et al., 2010; Pradhan et al., 2017; Tabibiazar et al., 2003; Targoff et al., 2013; van Weerd and Christoffels, 2016; Xin et al., 2007). However, our understanding of the regulatory networks necessary to maintain cardiac cell identity, which also underlie cellular plasticity within vertebrate hearts, remains incomplete.

Nr2f (formerly Coup-tf) TFs play a prominent role in vertebrate atrial cardiomyocyte (AC) differentiation and identity maintenance. Mutations in *NR2F2* are associated with a spectrum of congenital heart defects, including atrial septal defects (Al Turki et al., 2014; Nakamura et al., 2011; Qiao et al., 2018; Upadia et al., 2018). Within human cardiomyocyte-derived stem cell models, Nr2f1 and Nr2f2 are required for AC differentiation (Churko et al., 2018; Devalla et al., 2015). Global knockout (KO) of *Nr2f2* in mice and *nr2f1a* mutant zebrafish have shown that, *in vivo*, these factors are necessary to promote AC differentiation (Duong et al., 2018; Pereira et al., 1999). However, subsequent to heart tube formation, murine *Nr2f2* and zebrafish *nr2f1a* also are needed to maintain AC identity. Conditional KOs in murine cardiomyocytes using the *Myh6-Cre* showed that Nr2f2 is necessary to repress ventricular identity within ACs (Wu et al., 2013a). Similarly, our recent work showed that zebrafish Nr2f1a is necessary to repress the acquisition of ventricular cardiomyocyte (VC) identity within a specific subset of sensitized cardiomyocytes adjacent to the AVC, as well as to repress pacemaker cardiomyocyte (PC) identity at the venous pole via the maintenance of *nkx2.5* expression (Martin et al., 2023). Despite these reiterative requirements, our understanding of how Nr2f TFs function within these regulatory networks during heart development remains incomplete.

In mice, the TF Isl1 is expressed in the later differentiating second heart field and is crucial for the differentiation of cardiac cells at both the arterial and venous poles of the heart (Cai et al., 2003; de Pater et al., 2009). In contrast to this role in mice, Isl1 in zebrafish does not have a prominent role in the differentiation of cardiac cells, in particular cardiomyocytes at the arterial pole (de Pater et al., 2009). Although the importance of Isl1 orthologues within the second heart field may differ between mice and zebrafish, Isl1 has conserved requirements promoting the differentiation of PCs (de Pater et al., 2009; Liang et al., 2015; Sun et al., 2007; Tessadori et al., 2012; Weinberger et al., 2012). Consistent with this later requirement, its expression becomes restricted to the PCs within the sinoatrial node (SAN) (Ren et al., 2021). Similarly in zebrafish, after the heart tube forms, Isl1 becomes expressed at the venous pole and is necessary for PC differentiation (Tessadori et al., 2012). Previous work using human embryonic stem cells (hESCs) demonstrated that Isl1 can inhibit *NR2F1* in the differentiation of ACs (Quaranta et al., 2018). However, our recent work supports the observation that Nr2f1a functions within a regulatory network that inhibits Isl1 expression in ACs to repress PC identity (Martin et al., 2023). Thus, we do not have a clear understanding of the functional relationship between these Nr2f and Isl1 TFs in vertebrate heart development.

Here, we examined the relationship of Nr2f1a and Isl1 within the developing zebrafish heart. During earlier stages of cardiogenesis, we found that *nr2f1a*;*isl1* mutant hearts have a greater expansion of the AVC and progressive acquisition of VC identity within the atrium than occurs in *nr2f1a* mutants. At subsequent stages of cardiogenesis, while there is an absence of PCs in *nr2f1a;isl1* mutant hearts, similar to that found in *isl1* mutants, we also observed that in *nr2f1a;isl1* mutant hearts a population of ACs lose cardiomyocyte marker expression and gain expression of epicardial cell (EC) markers. Lineage tracing revealed that these transdifferentiating ACs migrate out of the myocardium in *nr2f1a;isl1* mutant hearts. Single-cell RNA sequencing (scRNA-seq) of wild-type, *nr2f1a*, *isl1* and *nr2f1a*;*isl1* mutant hearts supports the observation that *nr2f1a*;*isl1* mutant ACs and ECs share common transcriptomic signatures, and corroborates that ECs in *nr2f1a;isl1* mutants express cardiomyocyte markers, while ACs express EC markers. Epistasis studies support the observation that ectopic expression of *tcf21* and *tbx18* in venous ACs is required for the acquisition of EC identity in *nr2f1a;isl1* mutant hearts. Together, our data indicate that *isl1* is unexpectedly required for initially enhancing Nr2f1a-mediated AC identity maintenance; however, subsequently, Nr2f1a and Isl1 are concurrently necessary to repress EC identity within remaining ACs at the venous pole of the heart.

## RESULTS

### Nr2f1a and Isl1 functionally interact to maintain AC identity

**Fig. 1.**
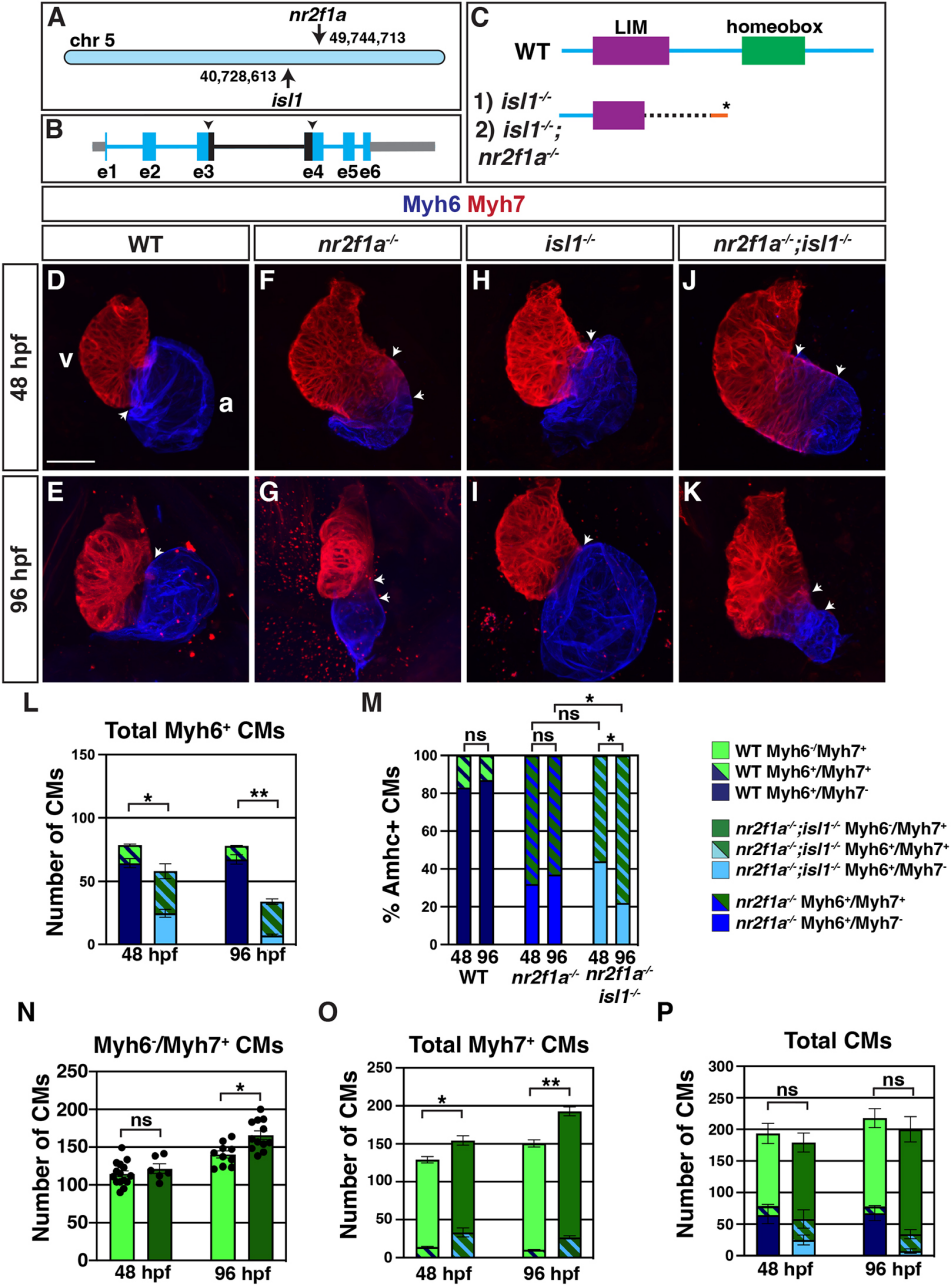
***nr2f1a;isl1* mutants have reduced atrial cardiomyocytes.** (A) Schematic of *nr2f1a* and *isl1* loci on chromosome 5. (B) Schematic of deletion in *isl1* locus. Location of guide RNAs and the boundaries of deletion (black arrowheads). (C) Schematic of predicted consequences of genomic deletion on Isl1 protein in *isl1* mutants and *nr2f1a;isl1* mutants. (D-K) Immunohistochemistry for Myh6 (blue) and Myh7 (red) in the wild-type, *nr2f1a*^−/−^, *isl1*^−/−^ and *nr2f1a*;*isl1^−/−^* hearts at 48 and 96 hpf. Overlap of Myh6 and Myh7 in the atrioventricular canal is indicated (white arrows). v, ventricle; a, atrium. Scale bar: 25 μm. 48 hpf: wild type (*n*=11), *nr2f1a^−/−^* (*n*=4), *isl1^−/−^* (*n*=13), *nr2f1a^−/−^;isl1^−/−^* (*n*=7); 96 hpf: wild type (*n*=16), *nr2f1a^−/−^*(*n*=11), *isl1^−/−^* (*n*=8), *nr2f1a^−/−^;isl1^−/−^* (*n*=8)*.* Individual channels with the merged images shown in D-K are presented in Fig. S1A-H. (L) Quantification of total Myh6^+^ (Myh6^+^/Myh7^−^+Myh6^+^/Myh7^+^) cardiomyocytes (CMs). (M) Proportions of Myh6^+^/Myh7^−^ and Myh6^+^/Myh7^+^ CMs at 48 and 96 hpf. Proportion of CMs for *nr2f1a* mutants are derived from Martin et al. (2023). (N-P) Quantification of Myh6^−^/Myh7^+^ (Myh7-only), total Myh7^+^ (Myh6^+^/Myh7^+^ and Myh6^−^/Myh7^+^) and total CMs. 48 hpf: wild type (*n*=15) and *nr2f1a^−/−^;isl1^−/−^* (*n*=6); 96 hpf: wild type (*n*=10) and *nr2f1a^−/−^;isl1^−/−^* (*n*=12). *P=0.05-0.001; **P<0.001 (ANOVA with multiple comparisons in L,N-P; Fisher's exact test in M).

We have previously found that Nr2f1a functions upstream of Nkx2.5 to repress PC identity in ACs of the embryonic zebrafish heart (Martin et al., 2023). However, we do not fully understand the functional relationship of Nr2f1a to other effectors of the PC regulatory network, such as Isl1, which, in contrast to Nkx2.5, promotes PC differentiation (Liang et al., 2015; Ren et al., 2021; Sun et al., 2007; Tessadori et al., 2012). To investigate the relationship between *nr2f1a* and *isl1*, we generated *nr2f1a;isl1* mutants. *nr2f1a* and *isl1* are located within close proximity (∼9 Mb apart) on chromosome 5 in zebrafish (Fig. 1A). Thus, the previously published *isl1* mutant allele (de Pater et al., 2009) could not be used and it was necessary to create a new *isl1* mutant allele on the same chromosome as our previously published *nr2f1a* mutant allele (Duong et al., 2018; Martin et al., 2023). Using two highly efficient CRISPR/Cas9 guide RNAs that target exons 3 and 4 of the *isl1* locus, we were able to generate a 1622 bp deletion on the same chromosome as our *nr2f1a* mutant allele (Fig. 1B). This guide RNA pair was so efficient that we were able to recover the same 1622 bp deletion in founders without the *nr2f1a* mutant allele, which served as controls. These large deletions are predicted to remove part of the LIM domain and cause early truncation of the protein, similar to the previously published *isl1* mutant allele with a non-sense mutation (Fig. 1C) (de Pater et al., 2009). The new *isl1* mutant embryos exactly phenocopy the defects shown with the previously published *isl1* mutant allele, displaying pericardial edema and arrhythmias, as well as being immotile and not responsive to touch (de Pater et al., 2009), supporting that they are *isl1* loss-of-function alleles.

*nr2f1a* mutants have smaller atria coupled with an expansion of VC markers into the atrium (Fig. 1D-G and Fig. S1A-D″) (Dohn et al., 2019; Martin et al., 2023). However, *isl1* mutants have overtly normal chamber morphology and marker expression (Fig. 1D,E,H,I and Fig. S1A-B″,E-F″) (de Pater et al., 2009). Although Isl1 was previously shown to be required for differentiation of cardiomyocytes at the venous pole of the heart (de Pater et al., 2009), we found no difference in cardiomyocyte number in either chamber of our *isl1* mutants at 48 hpf using the *Tg(myl7:DsRed2-NLS)* transgene (Mably et al., 2003) (Fig. S2A), supporting that Isl1 likely affects the timing of venous cardiomyocyte differentiation, but not the total number of Myh6^+^ (previously called atrial myosin heavy chain; Amhc) cells within the atrium that will differentiate. Examining hearts of *nr2f1a;isl1* mutant embryos, we found that they overtly resemble the hearts of the *nr2f1a* mutants, having smaller atria and an expansion of Myh7 (previously called ventricular myosin heavy chain; Vmhc) expression into the atrium (Fig. 1D-G,J,K and Fig. S1A-D″,G-H″). However, in contrast to *nr2f1a* mutants, in which the expanded population of Myh6^+^/Myh7^+^ cardiomyocytes, which we classify as AVC cardiomyocytes, becomes progressively smaller as Myh6 expression recedes from the outflow region of the atrium (Fig. 1D-G and Fig. S1A-D″) (Martin et al., 2023), in *nr2f1a;isl1* mutant embryos, the region of Myh6^+^/Myh7^+^ expression appears to remain the same size, and Myh7 expression continues to expand towards the venous pole (Fig. 1F,G,J,K and Fig. S1C-D″,G-H″).

To examine the dynamics of these cardiomyocyte populations, we quantified the cardiomyocytes in the atrium (Myh6^+^/Myh7^−^), AVC (Myh6^+^/Myh7^+^) and ventricle (Myh6^−^/Myh7^+^) using the *Tg(myl7:DsRed2-NLS)* transgene at 48 and 96 hpf. Similar to what we showed in *nr2f1a* mutants (Martin et al., 2023), we found a decrease in the total number of Myh6^+^ (Myh6^+^/Myh7^−^+Myh6^+^/Myh7^+^) cardiomyocytes from 48 to 96 hpf in the *nr2f1a;isl1* mutant hearts (Fig. 1L). However, within the Myh6^+^ population, the number of Myh6^+^/Myh7^−^ cardiomyocytes decreased significantly from 48 to 96 hpf, while Myh6^+^/Myh7^+^ cardiomyocytes decreased only a small amount (Fig. 1L and Fig. S2B,C). This result contrasts with our previous analysis of the *nr2f1a* mutants at 48 and 96 hpf, which showed that the Myh6^+^/Myh7^+^ population in *nr2f1a* mutants becomes progressively smaller, while the diminished number of Myh6^+^/Myh7^−^ cardiomyocytes remains constant over the same stages (Martin et al., 2023). Comparing the proportion of Myh6^+^/Myh7^+^ and Myh6^+^/Myh7^−^ cardiomyocytes at 48 and 96 hpf in the previously analyzed *nr2f1a* mutant hearts to the *nr2f1a;isl1* mutant hearts also supported the observation that the Myh6^+^/Myh7^−^ cardiomyocytes decreased in the *nr2f1a;isl1* mutant hearts (Fig. 1M). We also previously found that, in *nr2f1a* mutant hearts, total Myh7^+^ (Myh6^−^/Myh7^+^+Myh6^+^/Myh7^+^) cardiomyocyte expression is primarily expanded due to Myh7^+^ expression in the enlarged AVCs, with the increase in Myh6^−^/Myh7^+^ (Myh7-only) cardiomyocytes relative to wild-type hearts being relatively small and due to loss of Myh6 expression. However, in the *nr2f1a;isl1* mutants, we found an increase in Myh6^−^/Myh7^+^ cardiomyocytes relative to wild-type sibling embryos by 96 hpf (Fig. 1N), which complemented the exacerbated loss of Myh6^+^/Myh7^−^ cardiomyocytes. Furthermore, the total number of Myh7^+^ (Myh6^−^/Myh7^+^+Myh6^+^/Myh7^+^) cardiomyocytes was increased at 48 and 96 hpf in the *nr2f1a;isl1* mutant hearts (Fig. S1O), while the total number of cardiomyocytes in hearts of *nr2f1a*;*isl1* mutants and wild-type sibling controls was the same (Fig. S1P). Thus, while AVC cardiomyocytes resolve to Myh7-only cardiomyocytes in *nr2f1a* mutants (Martin et al., 2023), these results suggest that in *nr2f1a;isl1* mutant hearts cardiomyocytes within the AVC resolve to VC identity while there is potentially a continued Myh7 expansion into ACs (Myh6^+^ cardiomyocytes) toward the venous pole. Overall, these data indicate that, unexpectedly, loss of Isl1 exacerbates the expansion of VC identity and failure to maintain AC identity found in *nr2f1a* mutant hearts.

### *nr2f1a;isl1* mutants lack PCs

**Fig. 2.**
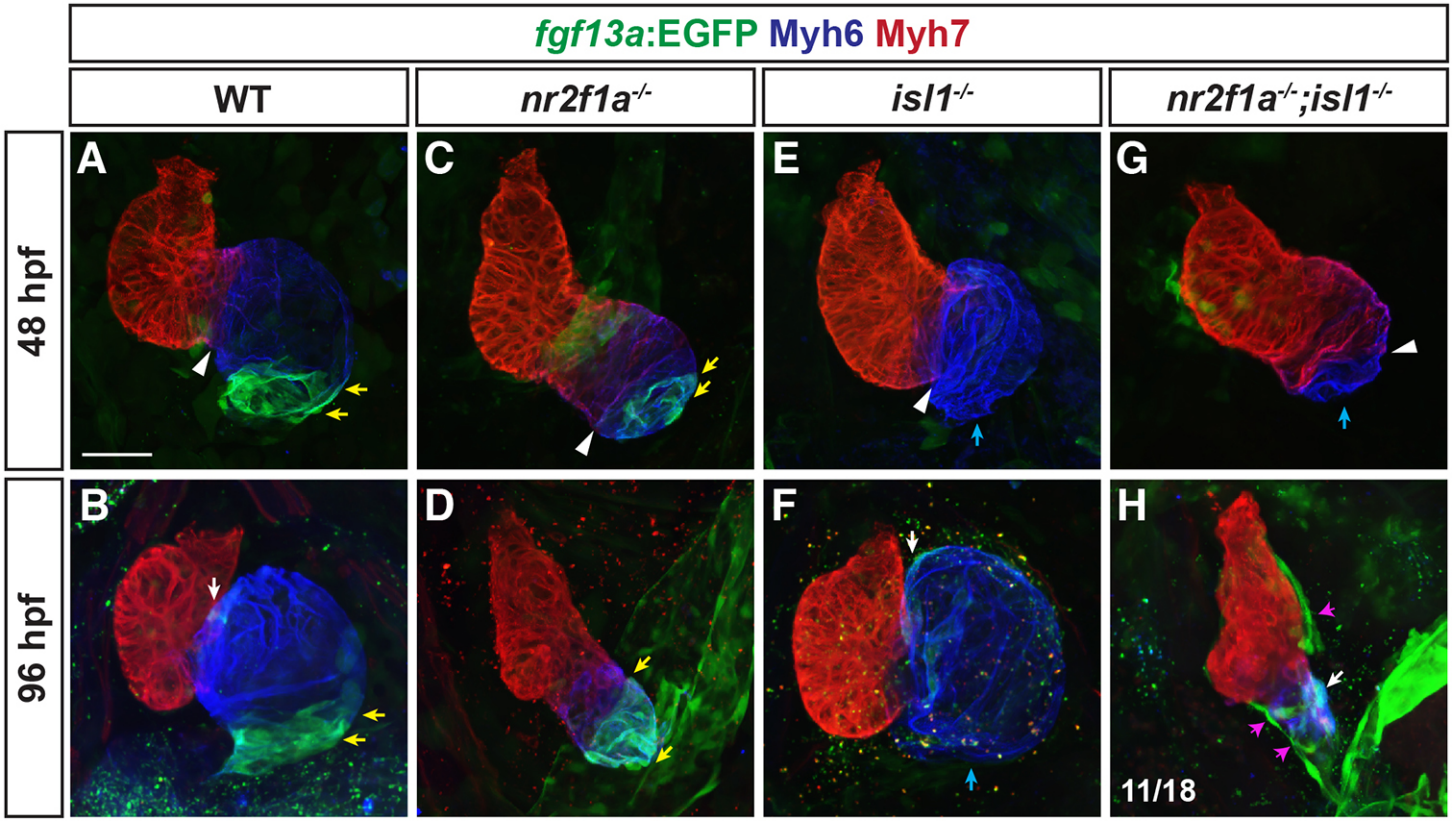
***nr2f1a*;*isl1* mutants lack pacemaker cardiomyocytes.** (A-H) Immunohistochemistry for Myh6 (blue), Myh7 (red) and *fgf13a*:EGFP (green) in hearts of wild-type, *nr2f1a* mutant, *isl1* mutant and *nr2f1a;isl1* mutant embryos at 48 and 96 hpf. The extent of Myh7 expression within the hearts (white arrowheads), the limits of *fgf13a:*EGFP expression within the heart (yellow arrows), the lack of *fgf13a:*EGFP expression (blue arrows), the AV node (white arrow) and *Fgf13a*:EGFP^+^ cells on the surface of the heart (magenta arrows) are shown. Representative image in H depicts the phenotype observed in the majority of *nr2f1a;isl1* mutant hearts (11/18) for this experiment. The 96 hpf wild-type heart in B is also presented in Fig. S3A for comparison to the phenotype observed in the minority of *nr2f1a;isl1* mutant hearts (7/18) presented in Fig. S3B. Scale bar: 25 μm. 48 hpf: wild type (*n*=15), *nr2f1a^−/−^* (*n*=9), *isl1^−/−^* (*n*=13) and *nr2f1a^−/−^;isl1^−/−^* (*n*=12); 96 hpf: wild type (*n*=13), *nr2f1a^−/−^*(*n*=11), *isl1^−/−^* (*n*=8) and *nr2f1a^−/−^;isl1^−/−^* (*n*=18).

In addition to preventing the acquisition of VC identity in the AVC/outflow region of the atrium, Nr2f1a restricts PC identity to the venous pole of the atrium (Fig. 2A-D) (Martin et al., 2023). Conversely, Isl1 is necessary for PC differentiation (Fig. 2E,F) (Abu Nahia et al., 2021; Tessadori et al., 2012). To examine PC differentiation in *nr2f1a;isl1* mutants, we used the *SqET33-mi59B* transgene (Poon et al., 2016), hereafter referred to as *fgf13a:EGFP*, to mark PCs. Like *isl1* mutants, *nr2f1a;isl1* mutant hearts lack *fgf13a:*EGFP at the venous pole of the heart at 48 hpf, despite their morphology phenocopying *nr2f1a* mutants (Fig. 2A,C,E,G). At 96 hpf, *fgf13a:*EGFP expands within the smaller atria of *nr2f1a* mutants, reflecting the expansion of PC identity (Fig. 2B,D), as we previously reported (Martin et al., 2023), while there is still an absence of *fgf13a:*EGFP at the venous pole in *isl1* mutants (Fig. 2B,F). In *nr2f1a*;*isl1* mutants, a significant proportion of the hearts overtly exhibited an expansion of *fgf13a:*EGFP at the venous pole by 96 hpf (Fig. 2B,H), which seemed reminiscent of the expansion of *fgf13a:*EGFP in *nr2f1a* mutants (Fig. 2D) (Martin et al., 2023). However, there was variability in the later appearance of *fgf13a:*EGFP expression within *nr2f1a*;*isl1* mutant hearts. A proportion of the hearts in *nr2f1a*;*isl1* mutant embryos overtly lacked the significant expansion of *fgf13a:*EGFP and had a clearer distinction between the diminished Myh6^+^ cardiomyocytes at the venous pole and adjacent *fgf13a:*EGFP^+^ cells of the inflow tract (Fig. S3A,B), suggesting that the reporter is marking the sinus venosus (Poon et al., 2016) and not PCs. The AV node was found to develop in the *isl1* and *nr2f1a;isl1* mutants, and also the *nr2f1a* mutants, although it can be difficult to observe due to the expansion of *fgf13a:*EGFP expression (Fig. 2B,D,F,H) (Martin et al., 2023).

**Fig. 3.**
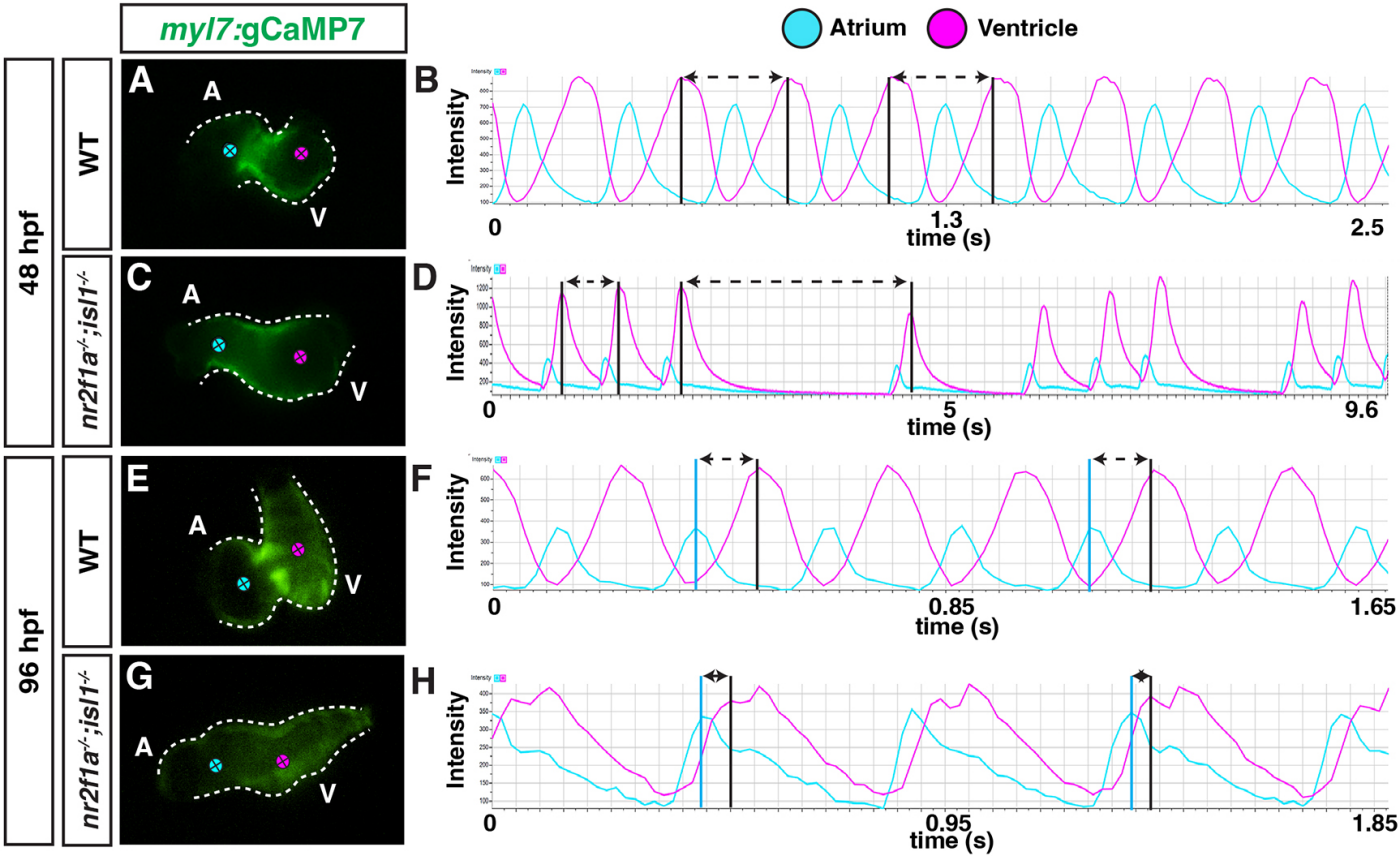
***nr2f1a;isl1* mutants functionally lack pacemaker cardiomyocytes.** (A,C,E,G) Images from videos of wild-type and *nr2f1a*;*isl1* mutant hearts carrying the *myl7:jGCaMP7c* transgene at 48 and 96 hpf. Magenta and blue dots indicate the location of jGCaMP7c fluorescence recordings in the ventricles and the atria, respectively. (B,D,F,H). Intensity traces of *myl7*:jGCaMP7c fluorescence from the ventricles (magenta) and atria (blue). Arrows between black lines in B and D indicate the time between maximum intensity of calcium waves in the ventricle. Arrows between blue and black lines in F and H indicate the time between maximum atrial intensity and initial peak in the ventricle. 48 hpf: wild type (*n*=5) and *nr2f1a^−/−^;isl1^−/−^* (*n*=3); 96 hpf: wild type (*n*=5) and *nr2f1a^−/−^;isl1^−/−^* (*n*=3).

The expansion of PC identity at the venous pole in *nr2f1a* mutants is accompanied by a loss of Nkx2.5 expression within the atrium (Martin et al., 2023). In contrast to *nr2f1a* mutant hearts, the PC repressor Nkx2.5 was maintained throughout the entire atria of the *nr2f1a;isl1* mutants through 96 hpf (Fig. S4A-D), suggesting that, despite apparent expression of *fgf13a:*EGFP in some hearts, PC identity was not expanded. To determine if there is a functional pacemaker in *nr2f1a;isl1* mutant hearts, we examined cardiac contractions and conduction using high-speed time-lapse bright-field imaging and fluorescent imaging of calcium transients with a transgenic cardiac-specific jGCaMP7c reporter (Dana et al., 2019) line we generated: *Tg(myl7:jGCaMP7c)*. At 48 hpf, *nr2f1a;isl1* mutants had significantly slower heart rates and periodic pausing compared to wild-type sibling embryos (Fig. 3A-D, Fig. S4E), similar what is found in *isl1* mutants (de Pater et al., 2009), supporting an absence of PCs and SAN activity. Interestingly, by 96 hpf, the periodic pausing of contractions and calcium transients diminished considerably, and the heart contractions were more consistent in the *nr2f1a;isl1* mutants embryos than at 48 hpf (Fig. 3E-H), which we hypothesize is due to development of the AV node, as it can function as a secondary pacemaker (Abu Nahia et al., 2021). Thus, *nr2f1a;isl1* mutants functionally lack PCs at the venous pole and their remaining ACs do not transdifferentiate into PCs.

### ACs take on EC identity in *nr2f1a;isl1* mutants

**Fig. 4.**
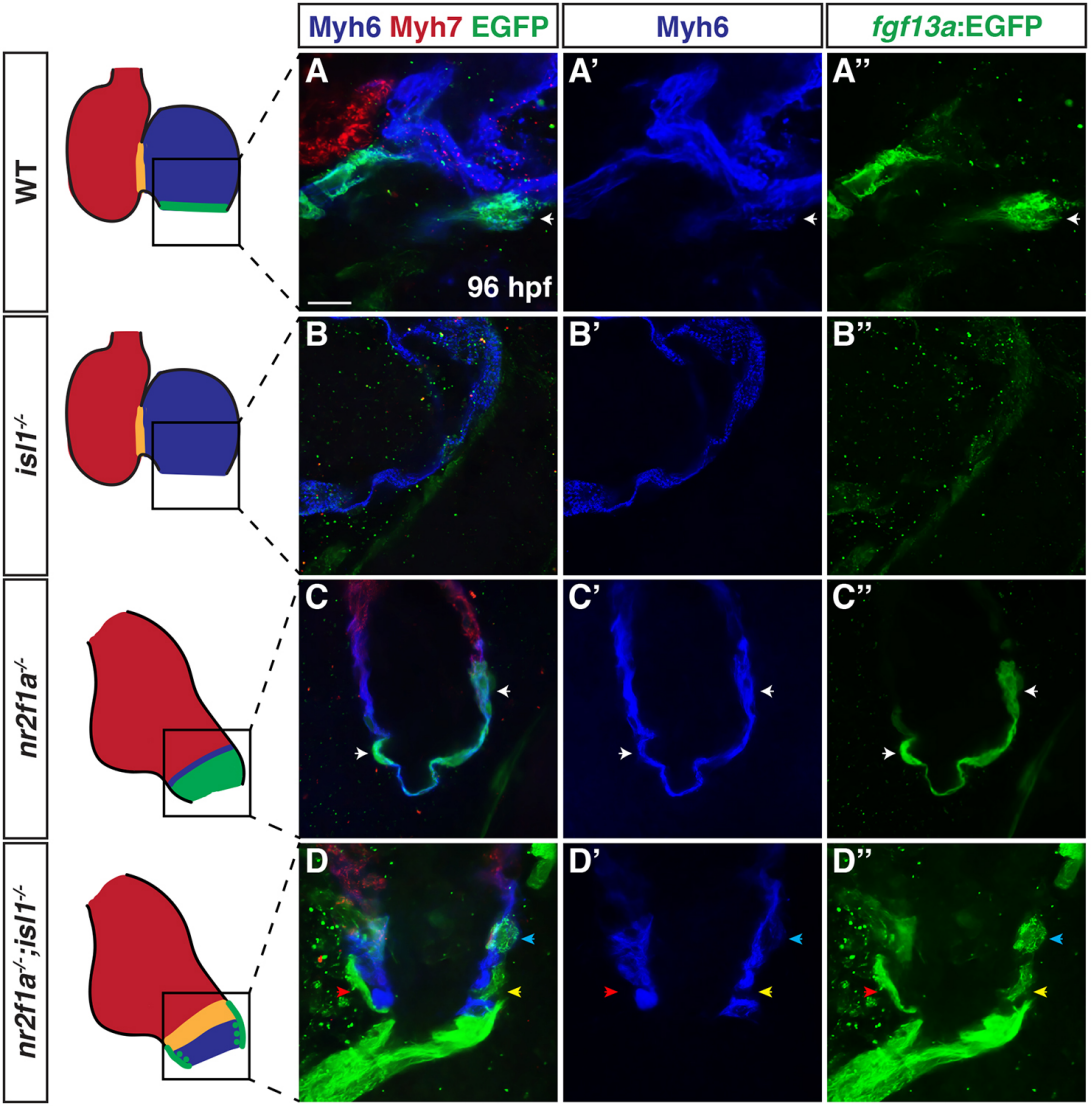
**Cells in the venous atria of *nr2f1a;isl1* mutants lack Myh6.** (A-D″) Optical sections of the venous pole of wild-type, *isl1*^−/−^*, nr2f1a*^−/−^ and *nr2f1a*^−/−^*;isl1*^−/−^ hearts at 96 hpf stained for Myh6 (blue), Myh7 (red) and *fgf13a:*EGFP (green). In the schematics, Myh7 (red), Myh6 (blue), Myh6 + Myh7 (orange) and *fgf13a*:EGFP (green) are shown. Overlap of Myh6 and *fgf13a*:EGFP in myocardium in A-A″ and C-C″ (white arrows). (D-D″) A *fgf13a:*EGFP^+^ cell in a gap of Myh6 (yellow arrows), a *fgf13a:*EGFP^+^ cell on the outside of the atrial myocardial wall (red arrows) and a *fgf13a:*EGFP^+^/Myh6^+^ cell in the atrial myocardial wall (blue arrows). Scale bar: 10 μm. Wild type (*n*=9), *nr2f1a^−/−^* (*n*=11), *isl1^−/−^* (*n*=4) and *nr2f1a^−/−^;isl1^−/−^* (*n*=13).

In addition to its apparent expansion within the atrial myocardial layer, we noticed that in *nr2f1a;isl1* mutant hearts the *fgf13a:EGFP* reporter appeared to label ECs on the ventricle and atrium at 96 hpf (Fig. 2H), which prompted us to more closely inspect *fgf13a:EGFP* reporter expression in wild-type embryos also carrying the *Tg(tcf21:H2A-mCherry)* transgene, a reporter of EC nuclei (Cao et al., 2017). We found that the *fgf13a:EGFP* transgene is expressed at low levels in sparse wild-type ECs and in proepicardial organ (PEO) cells adjacent to the venous pole and ventricle (Fig. S5A-A‴), in addition to PCs. Since *fgf13a:*EGFP appeared to be expressed more prominently in ECs in some *nr2f1a;isl1* mutant embryos, we examined the effect on ECs in *nr2f1a;isl1* mutants in the *Tg(tcf21:NLS-GFP)* line (Wang et al., 2011). We found that there was variability in the number of ECs on the ventricle in *nr2f1a;isl1* mutants: from sparse EC coverage to completely lacking ventricular ECs (Fig. S6A-C). However, there was an accumulation of ECs at the venous pole surrounding and immediately adjacent to the venous atrium (Fig. S6A,B), which is never observed in wild-type embryos. To determine which cells the *fgf13a:EGFP* transgene was labeling in the venous pole of *nr2f1a;isl1* mutant hearts, we examined optical sections from higher magnification confocal images. In wild-type embryo hearts at 96 hpf, the *fgf13a*:EGFP reporter overlapped with Myh6^+^ cells at the venous pole (Fig. 4A-A″), consistent with expression in PCs. As shown previously, *fgf13a:*EGFP was not expressed at the venous pole in *isl1* mutants (Fig. 4B-B″), while in *nr2f1a* mutant hearts, *fgf13a:*EGFP was expanded and overlapped with Myh6^+^ ACs (Fig. 4C-C″), consistent with their transdifferentiation to PCs (Martin et al., 2023). Interestingly, in *nr2f1a;isl1* mutant hearts, we observed three populations of *fgf13a*:EGFP^+^ cells at the venous pole: *fgf13a*:EGFP*^+^* cells that were Myh6^+^ and distinct from the AV node; *fgf13a*:EGFP^+^ cells that were on the surface of the atrium, consistent with expression in ECs; and *fgf13a:*EGFP*^+^* cells within gaps of Myh6^+^ cardiomyocytes in the venous atria (Fig. 4D-D″). Importantly, *fgf13a:*EGFP^+^ cells on the surface and within gaps of the Myh6^+^ myocardium concurrently expressed the *tcf21:H2A-mCherry* transgene, marking them as ECs (Fig. S5B-B‴).

**Fig. 5.**
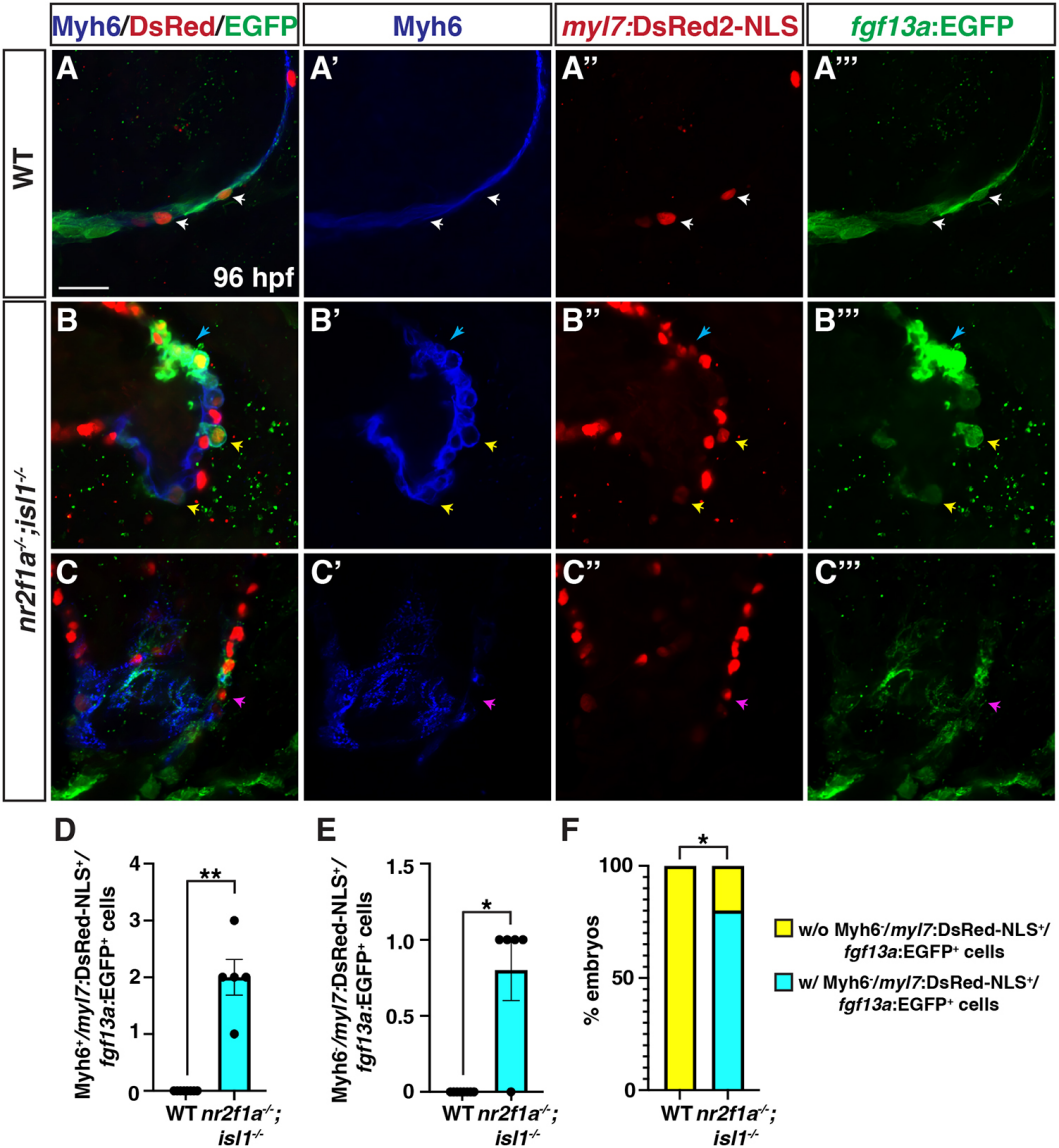
**Co-expression of cardiomyocyte markers with *fgf13a*:EGFP in *nr2f1a*;*isl1* mutant hearts.** (A-C‴) Optical sections of the venous pole of wild-type and *nr2f1a^−/−^*;*isl1*^−/−^ hearts at 96 hpf stained for Myh6 (blue), *myl7*:DsRed2-NLS (red) and *fgf13a*:EGFP (green). (A-A‴) Myh6^+^/*myl7*:DsRed2-NLS^+^/*fgf13a*:EGFP^+^ cells (pacemaker cardiomyocytes) in wild-type heart (white arrows). (B-B‴) Individual Myh6^+^/*myl7*:DsRed2-NLS^+^/*fgf13a*:EGFP^+^ cells in *nr2f1a^−/−^*;*isl1*^−/−^ heart (yellow arrows). (B-B‴) Myh6^+^/*myl7*:DsRed2-NLS^+^/*fgf13a*:EGFP^+^ AV node cardiomyocytes in *nr2f1a^−/−^*;*isl1*^−/−^ heart (blue arrows). (C-C‴) Myh6^−^/*myl7*:DsRed2-NLS^+^/*fgf13a*:EGFP^+^ cells in *nr2f1a^−/−^*;*isl1*^−/−^ heart (magenta arrows). Wild type (*n*=8) and *nr2f1a^−/−^;isl1^−/−^* (*n*=5). Scale bar: 10 μm. (D,E) Quantification of distinct Myh6^+^/*myl7*:DsRed2-NLS^+^/*fgf13a*:EGFP^+^ and Myh6^−^/*myl7*:DsRed2-NLS^+^/*fgf13a*:EGFP^+^ cells in wild-type and *nr2f1a^−/−^*;*isl1*^−/−^ embryos. (D) *P=0.0032; (E) **P=0.0161 (two-tailed Welch's *t*-test). (F) Proportion of embryos with at least one Myh6^−^/*myl7*:DsRed2-NLS^+^/*fgf13a*:EGFP^+^ cell. *P=0.007 (Fisher's exact test).

**Fig. 6.**
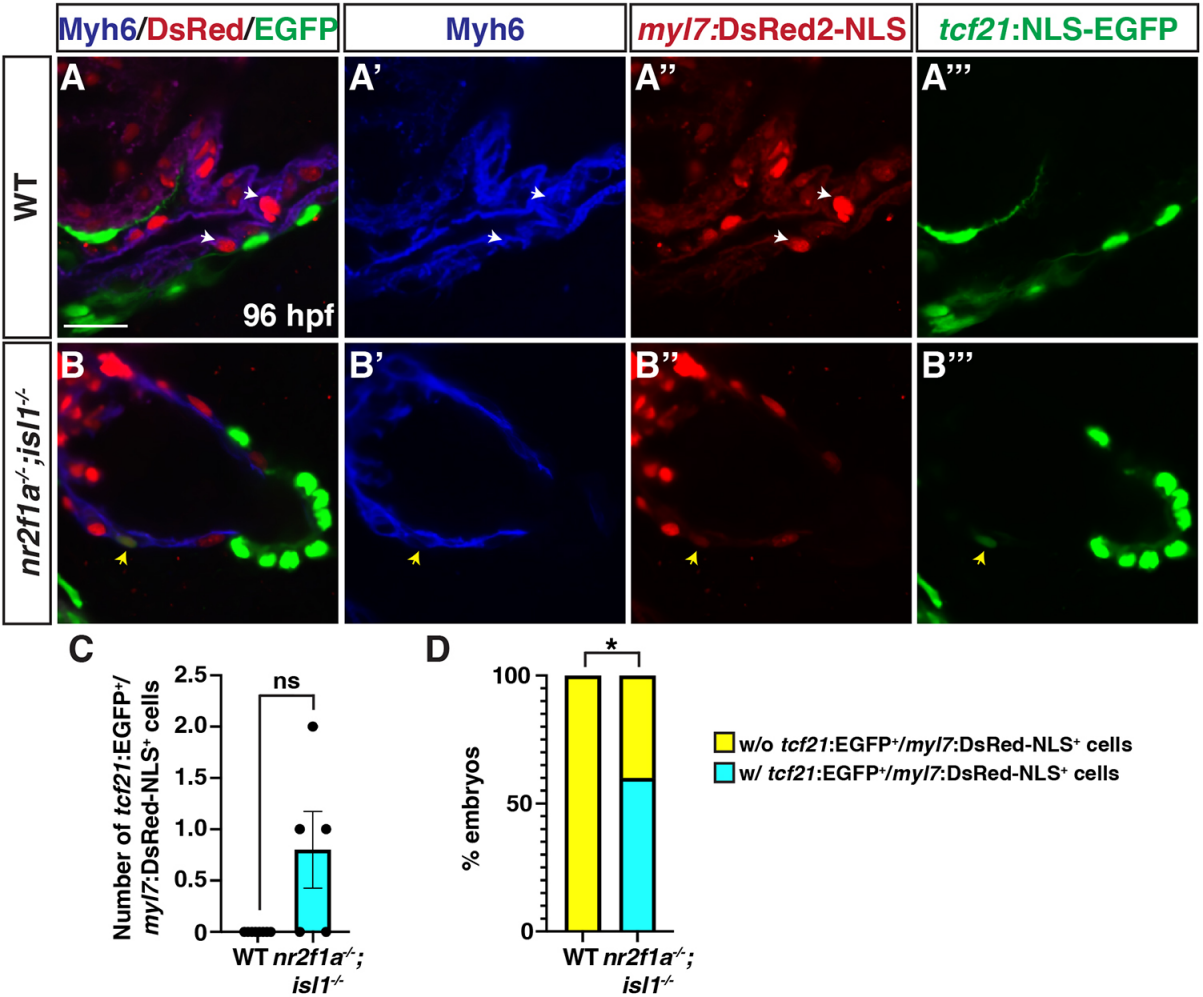
**Co-expression of cardiomyocyte markers with *tcf21:*NLS-EGFP in *nr2f1a*;*isl1* mutant hearts.** (A-B‴) Optical sections of the venous pole of wild-type and *nr2f1a^−/−^;isl1^−/−^* hearts at 96 hpf stained for Myh6 (blue), *myl7*:DsRed2-NLS (red) and *tcf21*:NLS-EGFP (green). (A-A‴) Representative Myh6^+^/*myl7*:DsRed2-NSL^+^/*tcf21*:NLS-EGFP^−^ cardiomyocytes in wild-type heart (white arrows). (B-B‴) Myh6^+^/*myl7*:DsRed2-NLS^+^/*tcf21*:NLS-EGFP^+^ cells in *nr2f1a*;*isl1* mutant heart (yellow arrows). Wild type (*n*=8) and *nr2f1a^−/−^;isl1^−/−^* (*n*=5). Scale bar: 10 μm. (C) Quantification of Myh6^+^/*myl7*:DsRed2-NLS^+^/*tcf21*:NLS-EGFP^+^ cells in wild-type and *nr2f1a^−/−^;isl1^−/−^* hearts. *P*=0.0993 (two-tailed Welch's *t*-test). (D) Proportion of embryos with at least one Myh6^+^/*myl7*:DsRed2-NLS^+^/*tcf21*:NLS-EGFP^+^ cell in wild-type and *nr2f1a^−/−^;isl1^−/−^* hearts. (D) *P=0.0350 (Fisher's exact test).

The presence of ECs within the myocardial layer of the *nr2f1a;isl1* mutants implies that, in the absence of Nr2f1a and Isl1, ECs could be infiltrating the myocardial layer and displacing ACs, or some of the remaining ACs at the venous pole could be transdifferentiating into ECs. To distinguish these possibilities, we first examined hearts in 96 hpf embryos carrying both the *myl7:DsRed2-NLS* and *fgf13a:EGFP* transgenes, with the rationale that a long half-life of the *myl7:*DsRed2-NLS reporter may mark AC nuclei that either have or have lost Myh6 expression and gained *fgf13a:*EGFP expression. In wild-type hearts, we found *myl7:*DsRed2-NLS^+^/*fgf13a:*EGFP^+^ cells co-expressing Myh6 exclusively at the venous pole of the heart, marking PCs (Fig. 5A-A‴). However, in *nr2f1a;isl1* mutant hearts, we observed co-expression of *myl7:*DsRed2-NLS^+^/*fgf13a*:EGFP^+^ with Myh6^+^ cardiomyocytes that were expressed sporadically throughout the diminutive atrial myocardium and were distinct from the putative AV node, some of which looked as if they are exiting the myocardial layer (Fig. 5B-B‴,D). Additionally, we observed *myl7*:DsRed2-NLS^+^/*fgf13a*:EGFP^+^ cells that were Myh6^−^ within these hearts (Fig. 5C-C‴,E), indicating that these co-labeled cells are concurrently expressed cardiomyocyte and EC markers. While the number of Myh6^−^/*myl7*:DsRed2-NLS^+^/*fgf13a*:EGFP^+^ cells/heart was lower than the number of Myh6^+^/*myl7*:DsRed2-NLS^+^/*fgf13a*:EGFP^+^ cells/heart in *nr2f1a;isl1* mutants, they were observed in almost all the hearts (Fig. 5F). To further explore if *nr2f1a;isl1* mutant hearts have cells that shared myocardial and EC markers, we next examined hearts at 96 hpf from embryos carrying the *myl7:DsRed2-NLS* and *tcf21:NLS-EGFP* transgenes. In wild-type embryos, at the venous pole of the heart *myl7:*DsRed2-NLS and *tcf21*:NLS-EGFP marked spatially distinct populations of cells, marking the nuclei of ACs in the myocardium and adjacent putative EC progenitors, respectively (Fig. 6A-A‴). However, in *nr2f1a;isl1* mutant hearts, we observed Myh6^+^ cells within the myocardial wall that co-expressed both transgenes, further supporting the observation that they possess both myocardial and epicardial characteristics (Fig. 6B-B‴). While the mean number of *myl7:*DsRed2-NLS^+^/*tcf21*:NLS-EGFP^+^ cells/heart (∼1 per/heart) only showed a trend relative to wild-type hearts (Fig. 6C), this was still comparable to the Myh6^−^/*myl7*:DsRed2-NLS^+^/*fgf13a*:EGFP^+^ cells/heart (Fig. 5D). However, at least one *myl7:*DsRed2-NLS^+^/*tcf21*:NLS-EGFP^+^ cell was found in the majority of the hearts examined (Fig. 6D).

**Fig. 7.**
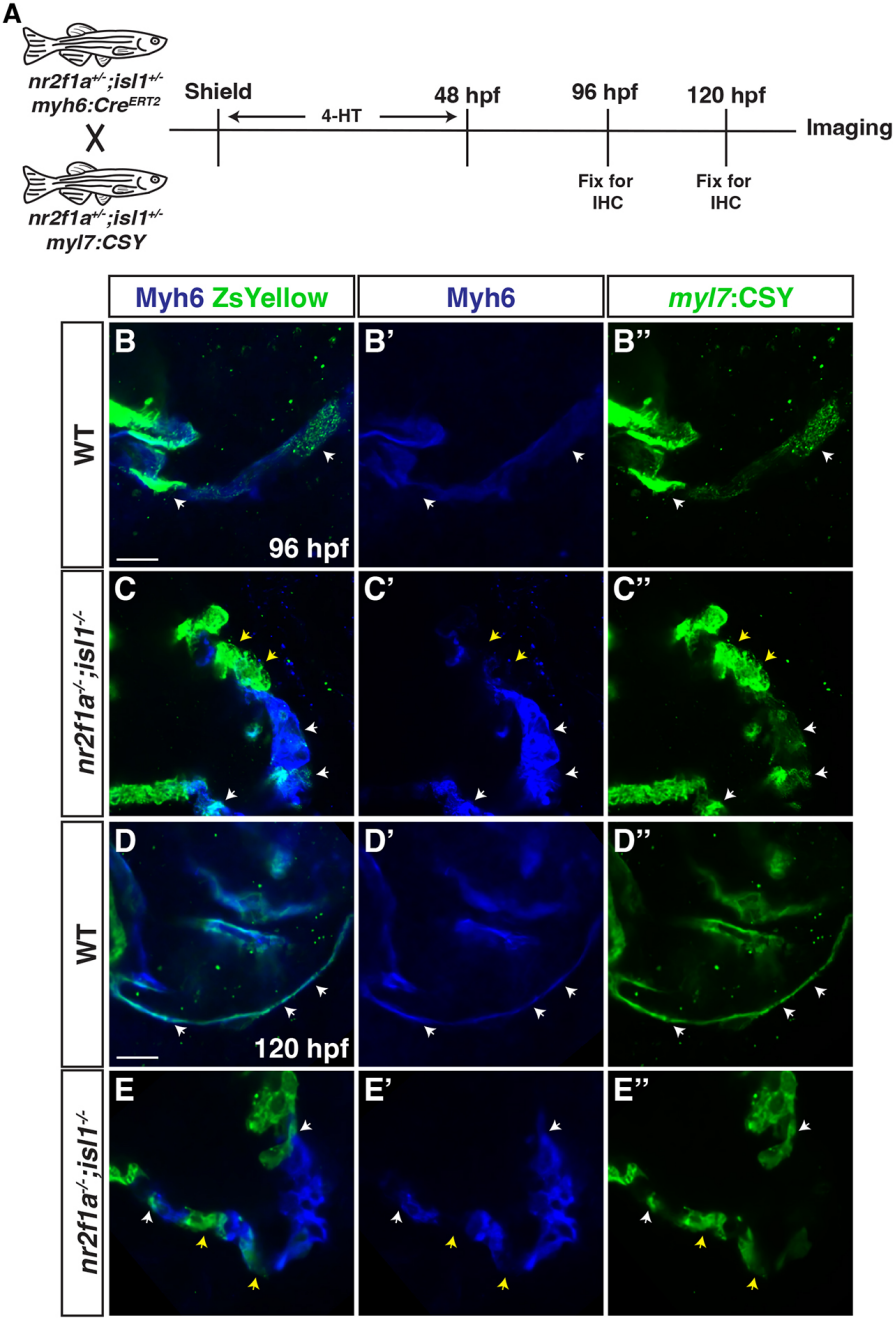
**Myh6 expression is lost in venous atrial cardiomyocytes of *nr2f1a*;*isl1* mutants.** (A) Schematic of lineage tracing of Myh6^+^ cells within the hearts of wild-type and *nr2f1a^−/−^;isl1^−/−^* embryos. (B-E″) Optical sections of the venous heart in wild-type and *nr2f1a^−/−^;isl1^−/−^* embryos at 96 hpf (B-C″) and 120 hpf (D-E″) stained for Myh6 (blue) and *myl7*:ZsYellow (green). (B-E″) Myh6^+^/ZsYellow^+^ cells (white arrows). (C-C″,E-E″) Myh6^−^/ZsYellow^+^ cells (yellow arrows). Scale bars: 10 μm. 96 hpf: wild type (*n*=2) and *nr2f1a^−/−^;isl1^−/−^* (*n*=13). 120 hpf: wild type (*n*=7) and *nr2f1a^−/−^;isl1^−/−^* (*n*=9).

Next, to determine if Myh6^+^ cardiomyocytes lose expression in *nr2f1a;isl1* mutant hearts, we performed lineage tracing with the *myh6:Cre^ERT2^* and *myl7:loxP-Cyan-Stop-loxP-ZsYellow* (*myl7:CSY*) transgenes (Zhang et al., 2013; Zhou et al., 2011). Embryos were treated with 4-hydroxytamoxifen (4-HT) from shield stage to 48 hpf, at which point the 4-HT was washed out, and embryos were allowed to develop until 96 or 120 hpf for analysis (Fig. 7A). In wild-type hearts of 4-HT treated *myh6:Cre^ERT2^*;*myl7:CSY* embryos, Myh6 and ZsYellow expression overlapped in the atria at both 96 and 120 hpf, indicating that Myh6^+^ ACs maintain Myh6 expression at later timepoints (Fig. 7B-B″,D-D″). Unlike wild-type sibling embryos, in *nr2f1a;isl1* mutants, there were two populations of ZsYellow^+^ cardiomyocytes within the atrium: the first co-expressed Myh6 and ZsYellow, similar to wild-type hearts; and the second population was ZsYellow^+^ and Myh6^−^, suggesting that these cells expressed Myh6 at earlier timepoints, but lost that expression by later timepoints (Fig. 7C-C″,E-E″). Cumulatively, these data suggest that, in the absence of both Nr2f1a and Isl1, ACs lose their myocardial identity and take on EC identity.

### Myocardial to epicardial transdifferentiation is not dependent on cardiac contractions

Our data suggest that *nr2f1a;isl1* mutant hearts have a progressive decrease in ACs (Myh6^+^/Myh7^−^ cardiomyocytes) (Fig. 1M), which we posited could be due to transdifferentiating ECs exiting the myocardial layer, as normally ECs undergo a contraction-dependent migration from the PEO to the ventricle (Peralta et al., 2013). To determine if cardiac contractions contribute to the emigration of transdifferentiated ECs from the diminutive atria of *nr2f1a*;*isl1* mutants, we injected embryos carrying the *tcf21:NLS-EGFP* transgene with the established *tnnt2a* morpholino (MO), which inhibits cardiac contraction (Sehnert et al., 2002). Wild-type sibling *tnnt2a* MO-injected embryos lack *tcf21:*NLS-EGFP^+^ cells on the ventricle, though they are retained in the PEO (Fig. S7A-B″). The hearts of *nr2f1a;isl1* mutants injected with the *tnnt2a* MO retained expression of *tcf21:*NLS-EGFP*^+^* cells within the atrial myocardial layer (Fig. S7C-D″), indicating that cardiac contraction is not required for differentiation of *tcf21:*NLS-EGFP^+^ cells within their atria. *nr2f1a;isl1* mutant embryos injected with the *tnnt2a* MO had a marginally higher percentage of hearts with *tcf21:*NLS-EGFP^+^ cells compared to uninjected *nr2f1a;isl1* mutant embryos (Fig. S7E). However, this trend was not significant. Therefore, cardiac contractions do not appear to be required to promote the exit of transdifferentiating ECs from the atrial myocardial layer.

### Shared gene expression of *nr2f1a*;*isl1* mutant ACs and ECs

**Fig. 8.**
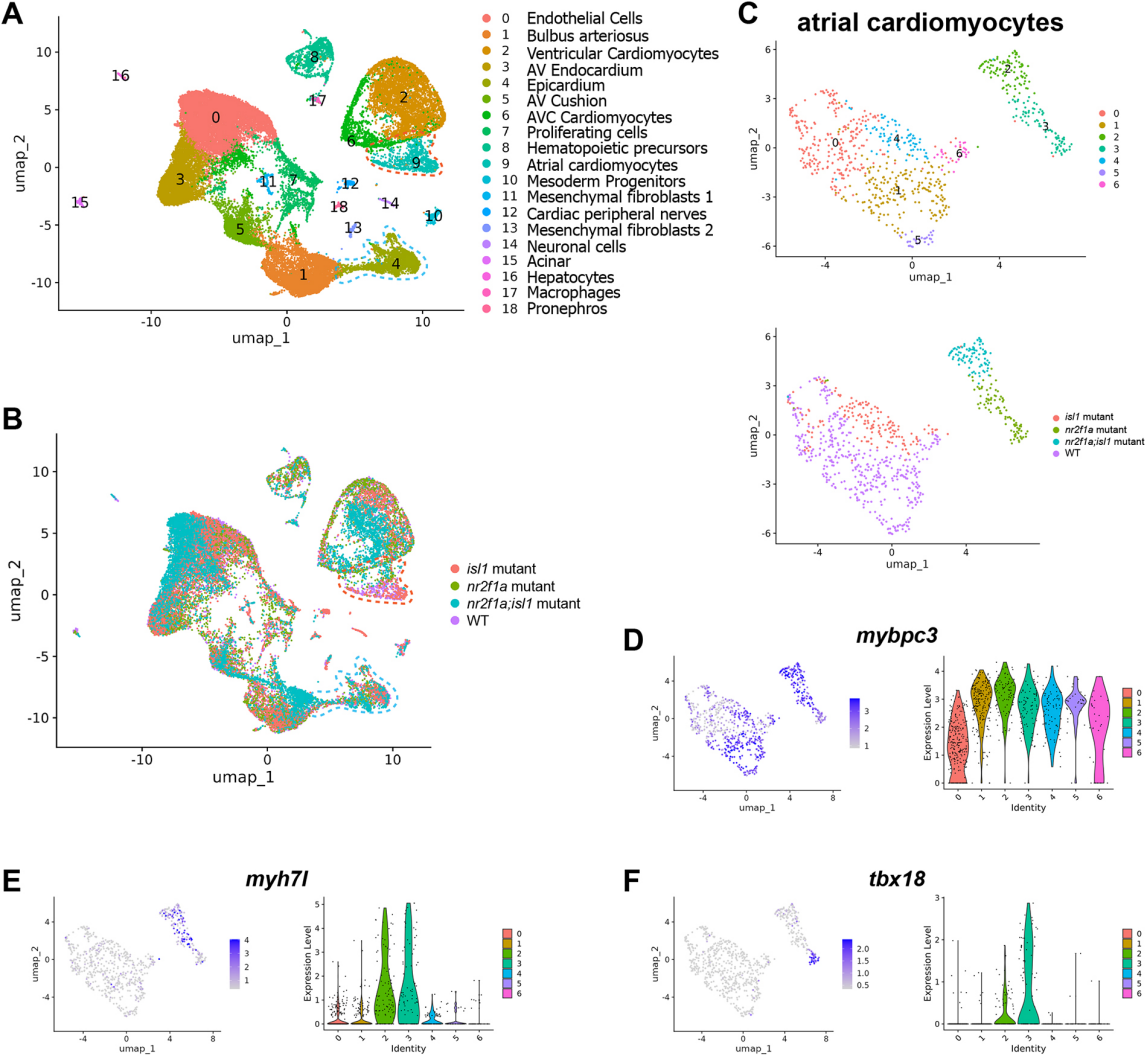
***nr2f1a*;*isl1* mutant atrial cardiomyocytes have ectopic ventricular and epicardial marker expression.** (A,B) UMAPs displaying the cell clusters identified and the conditions of the cells from the single-cell transcriptomics of the 96 hpf wild-type, *isl1* mutant, *nr2f1a* mutant and *nr2f1a*;*isl1* mutant hearts. (C) UMAPs showing the subclusters (top) and the conditions (bottom) of the cells from the atrial cardiomyocytes (ACs). (D-F) UMAPs (left) and violin plots (right) of the AC subclusters displaying expression of *mybpc3*, *myh7l* and *tbx18*.

To explore the gene signatures of ACs and ECs in these different conditions, we next performed single-cell transcriptomic analysis of hearts from wild-type, *nr2f1a*, *isl1* and *nr2f1a;isl1* mutant embryos at 96 hpf. The integration of the single-cell populations from these hearts indicated that we captured the major populations of cell types that would be expected, including ACs, VCs, ECs, endocardial cells, atrioventricular (AV) endocardial cells, AV cushion cells, bulbous arteriosus and proliferating cells (Fig. 8A,B; Fig. S8A; Table S1), which were assigned via manual curation of established markers and comparison to recently published single-cell datasets (Abu Nahia et al., 2024; Holowiecki et al., 2020; Weinberger et al., 2020). Cells from all four conditions were represented in the major clusters, while smaller clusters, some of which likely represent contamination from non-cardiac cell populations, lacked contributions from all the conditions (Fig. S8B). We observed that there was an increase in the proportion of VCs and a decrease in the proportion of ACs in the *nr2f1a* and *nr2f1a;isl1a* mutant hearts (Fig. S8B), corroborating effects on these cardiomyocyte populations at this stage that we observe from quantification in embryos (Fig. 1L-P and Fig. S2B,C) (Martin et al., 2023).

**Fig. 9.**
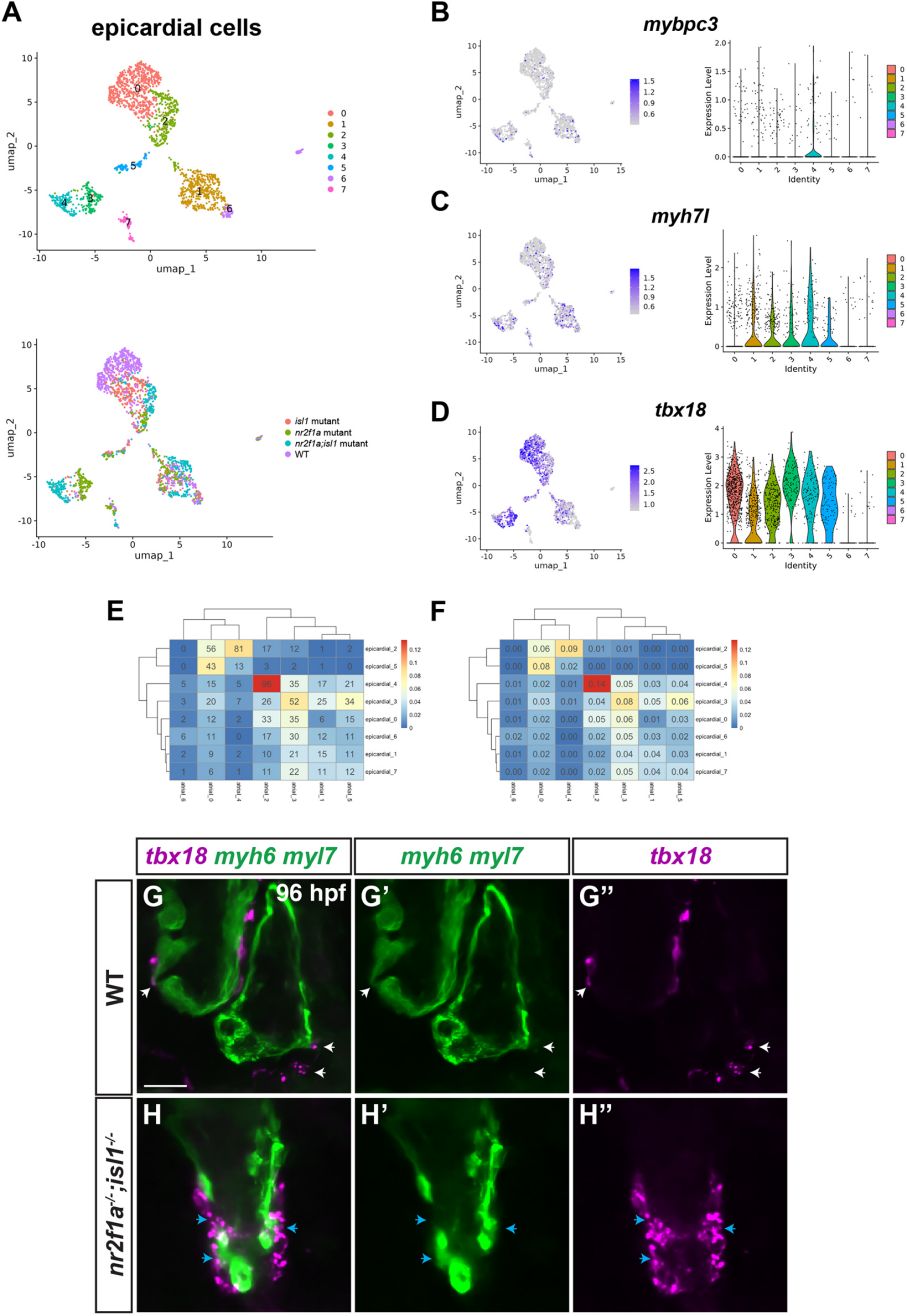
***nr2f1a*;*isl1* mutant atrial cardiomyocytes and epicardial cells share gene expression.** (A) UMAPs showing the subclusters (top) and the conditions (bottom) of the cells from the epicardial cells (ECs). (B-D) UMAPs (left) and violin plots (right) of the EC subclusters displaying expression of *mybpc3*, *myh7l* and *tbx18* in the atrial cardiomyocytes (ACs). (E,F) Number of enriched marker genes that are overlapping and Jaccard similarity scores in the subclusters of ACs and ECs. (G-H″) Optical sections of hybridization chain reaction (HCR) *in situ* for *tbx18*, *myl7* and *myh6* in wild-type sibling and *nr2f1a^−/−^;isl1^−/−^* embryos at 96 hpf. (G-G″) *tbx18* expression at the venous pole in the proepicardial organ (white arrows). (H-H″) *tbx18* expression in the atrial myocardial layer that lacks *myh6* expression and adjacent to *myh6* expression (blue arrows). Wild type (*n*=28) and *nr2f1a^−/−^;isl1^−/−^* (*n*=14). Scale bar: 10 μm.

To garner a better understanding of the specific effects of the mutations on transcriptional signatures in AC and EC populations, we performed subclustering (Figs 8A-C and 9A). The ACs were distributed into seven subclusters. Four of the AC subclusters predominantly comprised wild-type cells, including three AC populations (AC_C0, AC_C1 and AC_C6) and PCs (AC_C5) (Fig. 8C and Fig. S8C). AC_C4, which was immediately adjacent to the predominantly wild-type AC_C0 and AC_C1 clusters, solely comprised *isl1* mutant ACs. However, some *isl1* mutant ACs were also present in subclusters AC_C0 and AC_C6. PCs (AC_C5) were absent from the *isl1* mutant hearts (Fig. 8C and Fig. S8C), as would be expected from this and previous reports (Tessadori et al., 2012). In contrast to the wild-type and *isl1* ACs, the *nr2f1a* (AC_C3) and *nr2f1a;isl1a* (AC_C2) ACs clustered adjacent to each other and farther from the wild-type and *isl1* ACs (Fig. 8C), suggesting more similarity to each other than the wild-type and *isl1* populations due to the loss of *nr2f1a*. Examining gene expression enrichment within the subclusters, we found the AC markers *myh6* and *nr2f1a*, the transcripts of which are still expressed in mutants, and pan-cardiomyocyte markers, such as *mybpc3*, were expressed throughout the ACs and PCs (Fig. 8D; Fig. S9A,B; Table S2). The PC markers *isl1* and *shox2* showed ectopic expression in *nr2f1a* mutant hearts (AC_C3), consistent with ACs taking on PC marker identity (Fig. S9C,D) (Blaschke et al., 2007; de Pater et al., 2009; Hoffmann et al., 2013; Tessadori et al., 2012). However, these PC markers were lost in the adjacent *nr2f1a;isl1* mutant AC cluster (Fig. S9C,D), supporting the observation that the remaining ACs in the double mutants are not taking on PC identity. Although the *nr2f1a* (AC_C3) and *nr2f1a*;*isl1* (AC_C2) ACs still expressed AC markers, they also co-expressed VC markers, such as *myh7* and *myh7l* (Fig. 8E and Fig. S10A), supporting the observation that *nr2f1a* represses VC identity with ACs (Martin et al., 2023; Wu et al., 2013a). Given the potential transdifferentiation of ACs to ECs in the *nr2f1a;isl1* mutants, we examined the expression of established EC markers *wt1a*, *wt1b*, *tcf21* and *tbx18* in the AC clusters. Although there was no ectopic expression of *wt1a*, *wt1b* and *tcf21* in ACs (Fig. S10B-D), *tbx18* was ectopically expressed in ACs of both *nr2f1a* and *nr2f1a;isl1* mutants (Fig. 8F), indicating that *nr2f1a* is required to repress its expression within ACs.

We identified eight subclusters of ECs from the four conditions (Fig. 9A). EC_C0 primarily comprised wild-type and *isl1* mutant cells, while clusters EC_C1, EC_C2, EC_C5, EC_C6 and EC_C7 had cells from all four conditions (Fig. 9A and Fig. S8D). EC_C3 and EC_C4 clusters predominantly harbored *nr2f1a* and *nr2f1a*;*isl1* mutant ECs, respectively, and were located immediately adjacent to each other within the UMAP, yet more distant from the other clusters that contained wild-type and *isl1* mutant ECs (Fig. 9A and Fig. S8D). Gene expression analysis within the EC clusters showed that the EC markers *tcf21*, *wt1a*, *wt1b* and *tbx18* were enriched throughout all the EC clusters except EC_C6 (Fig. 9A and Fig. S9E-G). Gene ontology (GO) analysis of enriched genes within EC populations identified ‘heart morphogenesis’ (GO:0003007) as a term for the *nr2f1a*;*isl1* mutant cluster EC_C4 (Fig. S8E), which included genes such as *mybpc3*, *myh7l*, *tbx20*, *rbfox3a*, *wwtr1* and *alcama* (Table S3). Although some of these cardiac genes were also enriched in the *nr2f1a* mutant cluster EC_C3, GO analysis did not identify this term. These heart markers were not enriched in the other EC clusters. Interestingly, cardiac genes enriched in the *nr2f1a;isl1* mutant EC_C4 cluster were shared with those found in the *nr2f1a;isl1* mutant AC_C2 cluster, such as *mybpc3*, *myh7* and *myh7l* (Fig. 8D,E and Fig. S10E). Therefore, we asked if the *nr2f1a;isl1* mutant AC and EC clusters share ectopic gene expression. We examined the overlap in enriched genes identified within AC_C2 and EC_C4 clusters. We found that ∼12% of the enriched genes were shared between the *nr2f1a;isl1* mutant AC_C2 and EC_C4 clusters (Fig. 9E,F), including most of the aforementioned genes associated with heart morphogenesis, which was higher than comparison between any of the other AC and EC clusters. Thus, our data support the observations that ACs and ECs from *nr2f1a*;*isl1* mutants share ectopic gene signatures related to heart development and morphogenesis.

### *tcf21* and *tbx18* promote EC identity in *nr2f1a*;*isl1* mutants

We next wanted to understand whether critical regulators of EC development are required to promote the transition to EC identity in ACs of *nr2f1a*;*isl1* mutants. Overexpression of *wt1a* or *wt1b* in zebrafish is sufficient to drive a myocardial to EC fate switch within VCs, in which VCs delaminate from the myocardial layer (Marques et al., 2022). As this phenotype is reminiscent of what we observe in the atria of *nr2f1a;isl1* mutant hearts, we examined expression of *wt1a* using hybridization chain reaction (HCR) *in situ* and *wt1b* using the *wt1b*:*EGFP* transgene (Perner et al., 2007). *wt1a* and *wt1b*:EGFP expression were similar to *tcf21:*NLS-EGFP within the *nr2f1a;isl1* mutant atria at 96 hpf, with *wt1a*^+^ and *wt1b:*EGFP^+^ cells present within gaps of Myh6 expression (Fig. S11A-D‴). To determine whether either of these genes are required for the AC to EC transformation, we knocked-down *wt1a* and *wt1b* expression using CRISPR/Cas9 (Hoshijima et al., 2019). *Wt1a* knockdown (KD) in wild-type sibling embryos phenocopied established *wt1a* mutants and lacked *tcf21:*NLS-EGFP^+^ ECs on their ventricles (Fig. S12A-D) (Boezio et al., 2023). However, *wt1a* KD in *nr2f1a;isl1* mutants did not prevent the appearance of *tcf21:*NLS-EGFP^+^ cells within the atrial myocardium (Fig. S13A-B‴,D-E‴). KD of *wt1b*, for which there does not appear to be a requirement in early epicardial development (Sanz-Morejón et al., 2019), also did not affect the appearance of *tcf21:*NLS-EGFP^+^ cells within the atrial myocardium of *nr2f1a;isl1* mutant embryos (Fig. S13C-C‴,F-F‴).

**Fig. 10.**
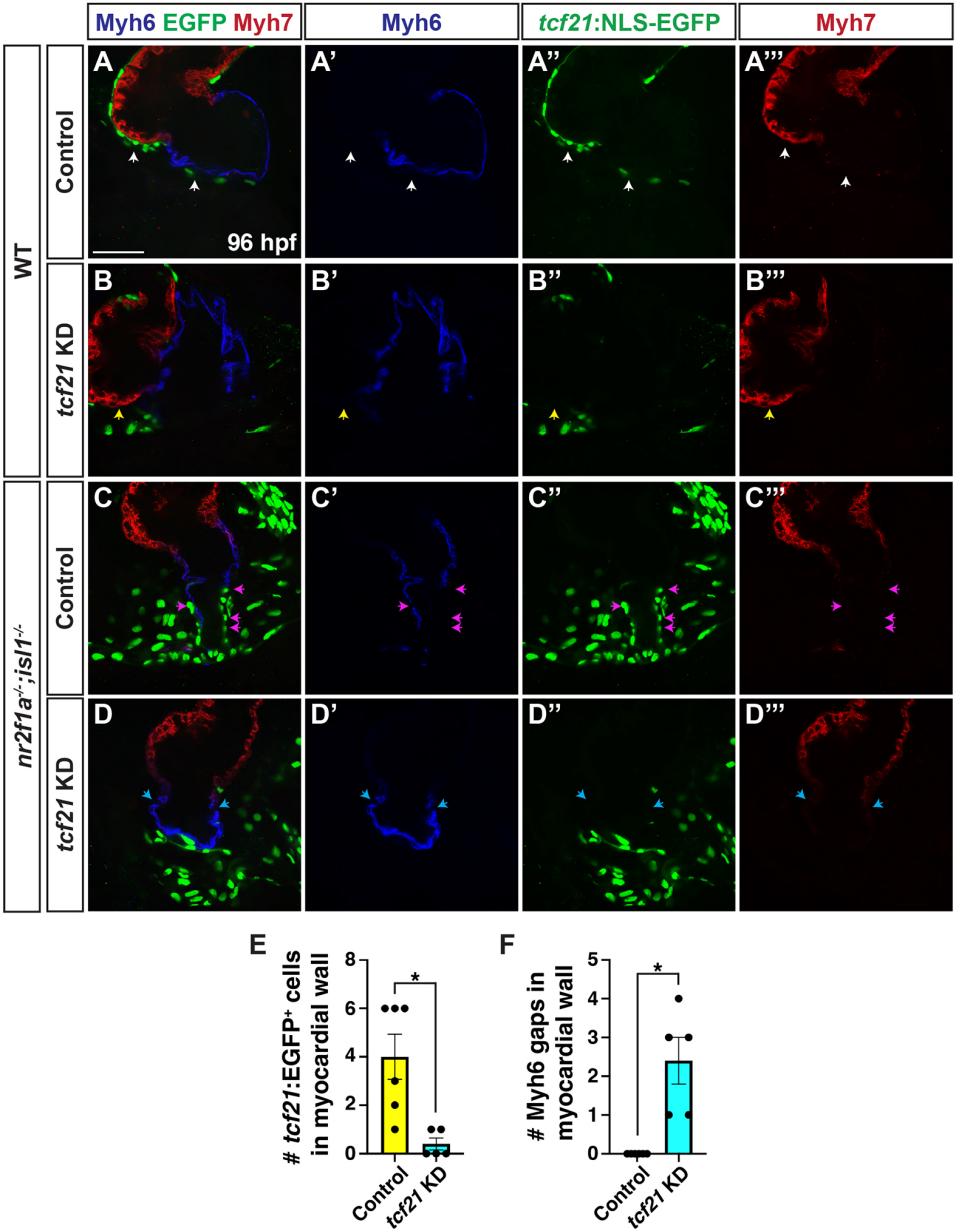
***tcf21* is required to promote epicardial cell gene expression in *nr2f1a;isl1* mutant atria.** (A-D‴) Optical sections of immunohistochemistry for Myh6 (blue), Myh7 (red) and *tcf21*:NLS-EGFP (green) in control and *tcf21* knockdown (KD) wild-type and *nr2f1a^−/−^;isl1^−/−^* hearts. (A-A‴) Epicardial cells (ECs) on the ventricle (white arrowheads). (B-B‴) The lack of ECs on the ventricle (yellow arrowheads). (C-C‴) *tcf21*:NLS-EGFP^+^/Myh6^−^ cells in myocardial wall (magenta arrowheads). (D-D‴) Gaps in Myh6 within the myocardial wall (blue arrowheads). Scale bar: 25 μm. Control: wild type (*n*=9) and *nr2f1a^−/−^;isl1^−/−^* (*n*=6); *tcf21* KD: wild type (*n*=9) and *nr2f1a^−/−^;isl1^−/−^* (*n*=5). (E) Quantification *tcf21*:NLS-EGFP^+^ cells within the control and *tcf21* KD *nr2f1a^−/−^;isl1^−/−^* hearts. *P=0.0106 (two-tailed Welch's *t*-test). (F) Quantification of gaps in Myh6^+^ myocardial wall of control and *tcf21* KD *nr2f1a^−/−^;isl1^−/−^* hearts. *P=0.0161 (two-tailed Welch's *t*-test).

In contrast to *wt1a* mutants, *tcf21* has a more limited requirement in promoting zebrafish EC development. *tcf21* mutant zebrafish embryos have a variable reduction in ECs that traverse to the ventricle compared to *wt1a* mutants (Boezio et al., 2023). Furthermore, *tcf21* was not sufficient to drive transformation of VCs to ECs, like ectopic expression of *wt1a* or *wt1b* (Marques et al., 2022). KD of *tcf21* recapitulated established mutants with a variable reduction in ventricular *tcf21:*NLS-EGFP^+^ ECs on wild-type hearts, with the most severe lacking ECs on their ventricles (Fig. S12E-H) (Boezio et al., 2023). In *nr2f1a;isl1* mutants, KD of *tcf21* produced a marked decrease in *tcf21:*NLS-EGFP^+^ cells within the atrial myocardial layer (Fig. 10A-E). Interestingly, there was an increase in the number of gaps in Myh6 expression that lacked *tcf21:*NLS-EGFP^+^ within the *nr2f1a;isl1* mutant atrial walls (Fig. 10D,D′,F). As the CRISPR guides target exons within *tcf21* (Fig. S12G), these should not directly affect expression of the transgenic BAC *tcf21:NLS-GFP* reporter, and its expression was not affected in other extra-cardiac tissues (Fig. 10B). Thus, *tcf21* depletion is sufficient to prevent venous ACs from expressing epicardial markers in *nr2f1a*;*isl1* mutants.

**Fig. 11.**
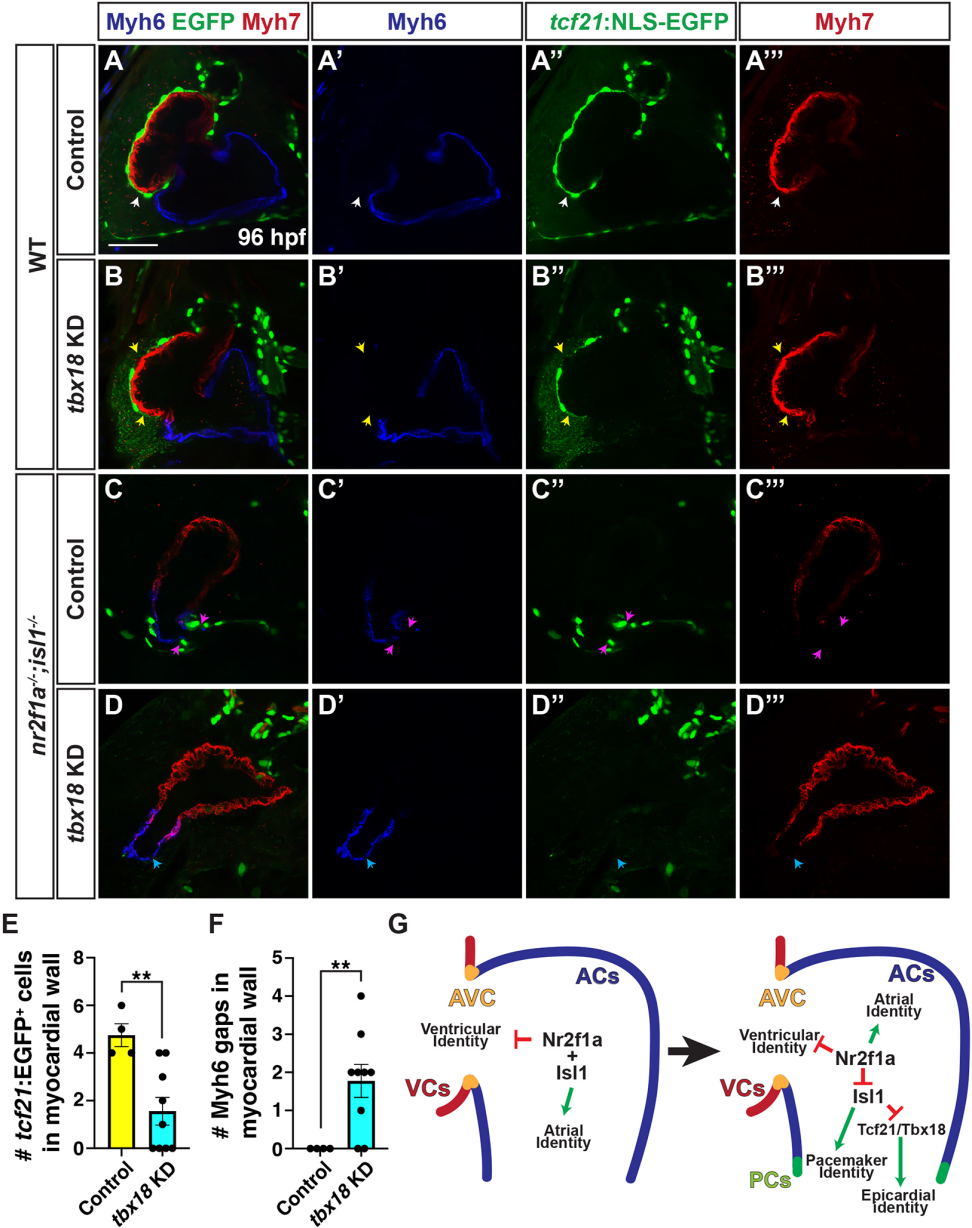
***tbx18* is required to promote epicardial cell gene expression in *nr2f1a;isl1* mutant atria.** (A-D‴) Optical sections of immunohistochemistry for Myh6 (blue), Myh7 (red) and *tcf21*:NLS-EGFP (green) in control and *tbx18* knockdown (KD) wild-type and *nr2f1a^−/−^;isl1^−/−^* hearts. (A-A‴) Epicardial cells (ECs) on the ventricle (white arrowheads). (B-B‴) The lack of ECs on the ventricle (yellow arrowheads). (C-C‴) *tcf21*:NLS-EGFP^+^/Myh6^−^ cells in myocardial wall (magenta arrowheads). (D-D‴) Gaps in Myh6 within the myocardial wall (blue arrowheads). Scale bar: 25 μm. Control: WT (*n*=4) and *nr2f1a^−/−^;isl1^−/−^* (*n*=4); *tbx18* KD: wild type (*n*=9) and *nr2f1a^−/−^;isl1^−/−^* (*n*=22). (E) Quantification *tcf21*:NLS-EGFP^+^ cells within the hearts of control and *tbx18* KD *nr2f1a^−/−^;isl1^−/−^* embryos. **P=0.0017 (two-tailed Welch's *t*-test). (F) Quantification of gaps in Myh6^+^ myocardial wall of hearts from control and *tbx18* KD *nr2f1a^−/−^;isl1^−/−^* embryos. **P=0.0035 (two-tailed Welch's *t*-test). (G) Model indicating functional interactions of Nr2f1a and Isl1 within the zebrafish heart. Initially, Isl1 augments Nr2f1a maintenance of atrial cardiomyocyte (AC) identity. Subsequently, Nr2f1a and Isl1 have opposing roles within the venous atrium, with Nr2f1a promoting working AC identity and Isl1 promoting atrial cardiomyocyte identity. In the absence of both Nr2f1a and Isl1, ACs fail to maintain identity and transdifferentiate into ECs via Tcf21 or Tbx18.

Although *tbx18* is implicated in the differentiation of EC-derived smooth muscle in mice (Wu et al., 2013b), a role has not been shown yet in zebrafish. Our single-cell transcriptomic analysis indicated that *tbx18* was ectopically expressed in the ACs of *nr2f1a;isl1* mutant hearts (Fig. 9D), which cannot be attributed to expression in transdifferentiating PCs. Consistent with the transcriptomic analysis, HCR showed that *tbx18* is broadly expressed in ECs that accumulate adjacent to the venous atrium, as well as within the pockets of Myh6^−^ cells within the atrial wall (Fig. 9G-H″), similar to *wt1a*, *wt1b* and *tcf21*. In wild-type sibling embryos, we found that KD of *tbx18* with CRISPR/Cas9 had a variable effect on the accumulation of ECs on the ventricle with the most severe completely lacking ECs on the ventricle (Fig. 11A-B‴ and Fig. S12I-L), reminiscent of *tcf21* mutants/KD (Fig. S12E-H) (Boezio et al., 2023). KD of *tbx18* in *nr2f1a;isl1* mutants produced a similar effect to *tcf21* KD, where there was a loss of *tcf21:*NLS-EGFP^+^ cells coupled with an increase in gaps in Myh6 expression (Fig. 11C-F). Therefore, our data indicate that *tcf21* or *tbx18* are required for the acquisition of EC fate in *nr2f1a;isl1* mutant ACs, but do not appear to be necessary for downregulation of Myh6 within ACs.

## DISCUSSION

Here, we have identified that Nr2f1a and Isl1 have multiple concurrent requirements that function in the maintenance of atrial myocardial identity (Fig. 11G). While *isl1* mutants have normal chamber morphology (de Pater et al., 2009; Tessadori et al., 2012), loss of Isl1 in *nr2f1a* mutants exacerbates the expansion of the AVC and ventricular markers, and leads to a reduction in Myh6^+^ ACs compared to *nr2f1a* mutants alone (Martin et al., 2023). In *nr2f1a;isl1* mutant hearts, ventricular markers eventually expand almost entirely to the venous pole. As Nr2f1a and Isl1 are both expressed in presumptive progenitor cells at the venous pole, while Isl1 is not expressed in differentiated ACs (de Pater et al., 2009; Duong et al., 2018), it suggests an early requirement for these factors that establishes the subsequent ability to refine and repress ventricular identity, as well as the later absence of PC expansion from the venous pole. Furthermore, we did not observe co-expression of PC and VC markers in *nr2f1a* mutants with IHC (Martin et al., 2023), though our single-cell transcriptomic data may suggest that there is eventually overlap, which would require further investigation. Ectopic expression of Isl1 can promote PC identity and represses ventricular identity in mouse ESCs and zebrafish embryos (Dorn et al., 2015; Liang et al., 2015). Thus, an earlier cooperative requirement of Nr2f1a and Isl1, and the ectopic expression of Isl1 in *nr2f1a* mutants may both contribute to limiting the expression and expansion of ventricular markers within ACs.

Within the small population of ACs at the venous pole of *nr2f1a;isl1* mutant hearts, there is an absence of PC differentiation, similar to *isl1* mutants (Abu Nahia et al., 2021; de Pater et al., 2009; Tessadori et al., 2012), supporting the observation that *isl1* is required to promote PC differentiation and the expansion of PC identity within ACs of *nr2f1a* mutants. Furthermore, some of the ACs in the diminutive atria of *nr2f1a;isl1* mutant hearts then progressively lose expression of Myh6, begin to express EC markers, including *wt1a*, *wt1b*, *tcf21* and *tbx18*, and appear to migrate out of the myocardial layer and into the epicardium. As we have not observed AC-to-EC transdifferentiation in either *nr2f1a* or *isl1* mutants alone, this suggests cooperative roles for these TFs in the repression of EC identity within ACs. As with repression of ventricular identity, the overlap of Nr2f1a and Isl1 likely occurs in cardiac progenitors at the venous pole. Consistent with an early requirement of Nr2f1a in maintenance of AC identity, we previously found that induction of Nkx2.5 prior to 24 hpf was able to repress conversion of ACs to PCs in the *nr2f1a* mutants (Martin et al., 2023). Thus, we posit that the failure to maintain identity AC and the acquisition of EC identity at later stages of cardiogenesis is due to cooperation between Nr2f1a and Isl1 TFs at earlier stages of cardiac differentiation when they have overlapping expression. However, future experiments are needed to definitively decipher the specific timing of their cooperative requirements.

Although we did not identify a distinct cluster of putative transdifferentiating cells that co-expressed AC and EC markers in *nr2f1a;isl1* mutant hearts from the single-cell transcriptomic analysis, we reasoned this could be because these cells are a relatively small number in *nr2f1a*;*isl1* mutant hearts and/or that these cells are not distinguishable from other ECs, due to a lack of spatial information. However, our transcriptomic analysis supports the observation that ACs and ECs within the *nr2f1a;isl1* mutant hearts share significant overlap of enriched gene expression. While loss of *nr2f1a* alone does lead to ectopic expression of many of these genes, loss of *isl1* and *nr2f1a* together enhances the shared set of enriched genes within the mutant AC and EC populations. These data support a regulatory hierarchy whereby in the absence of Nr2f1a, which maintains AC identity, and Isl1, which is needed to promote PC differentiation, a potential intermediate state between ACs and ECs is created, which we posit facilitates the ACs defaulting to an EC identity (Fig. 11G). In mice, hypomorphic *Nr2f2* mutants have defects in development of the ventricular epicardium and PEO cluster at the venous pole (Lin et al., 2012). The clustering of ECs at the venous pole and variable ventricular EC coverage in *nr2f1a;isl1* mutant hearts is reminiscent of the EC defects in hypomorphic *Nr2f2* mouse mutants (Lin et al., 2012). However, more work is needed to investigate the requirements for *nr2f1a* alone in epicardial development of zebrafish. Of the EC markers, the transcriptomic analysis shows that *tbx18* is ectopically expressed in ACs of *nr2f1a* mutants and *nr2f1a;isl1* mutants, suggesting it is required to repress *tbx18* within ACs. While *tbx18* is also expressed in PCs and can promote ectopic PC identity in mice (Kapoor et al., 2012; Wiese et al., 2009), our work suggests that, within this regulatory network, *tbx18* is repressed by Nr2f1a independently of Isl1 (Fig. 11G). Our data support the observation that, in the absence of Isl1, ectopic *tbx18* participates in promoting EC identity within mutant ACs.

There are multiple examples of conditions whereby if myocardial wall integrity is impaired, cardiomyocytes can be extruded from the myocardium (Rasouli et al., 2018; Rydeen and Waxman, 2016). Our data are consistent with the hypothesis that in addition to the transdifferentiation of Myh6^+^ cells to Myh7^+^ cells, a progressive conversion of ACs to ECs and exit from the atrial wall also contribute to the greater deficit of ACs in *nr2f1a;isl1* mutants compared to *nr2f1a* mutants. Interestingly, a recent study has shown that overexpression of *wt1a* or *wt1b*, but not *tcf21*, in zebrafish cardiomyocytes was sufficient to promote a fate transformation from VCs to EC-like identity and their egression from the ventricular wall (Marques et al., 2022). A conversion of ACs to EC-like cells with ectopic *wt1a* was not reported. Nevertheless, the ability of *wt1a* to induce EC identity also correlates with its greater requirement for EC migration from the PEO compared to *tcf21* (Boezio et al., 2023). In contrast to what may have been expected from the previous gain- and loss-of-function analyses (Marques et al., 2022), our results suggest that either *tcf21* or *tbx18*, both of which in zebrafish appear to have more variable requirements in the initial migration from the PEO, is necessary for the conversion to EC identity within ACs. Thus, the regulatory networks within the atrium and mechanisms underlying the transdifferentiation to EC identity appear to involve putatively more downstream factors, such as *tcf21* and *tbx18*, in the absence of both *nr2f1a* and *isl1*.

In conclusion, this work expands our knowledge of cardiomyocyte plasticity and the regulatory networks that promote and maintain AC identity. We find that Nr2f1a and Isl1 have concurrent requirements limiting ventricular identity and repressing EC identity within the atrial myocardium. As Nr2f1a functions upstream of Isl1 to repress SAN identity within ACs and loss of both Nr2f1a and Isl1 leads to an acquisition of EC identity at the venous pole, this suggests a series of putative binary decisions in the maintenance of AC identity, whereby EC identity may become a default state without input from both these transcription factors (Fig. 11G). Future work specifically examining the cis-regulatory elements regulated by the EC core transcription factors, as well as the cohort of enriched genes within ACs and ECs of the *nr2f1a*;*isl1* mutants, will allow us to decipher the precise transcriptional mechanisms underlying these regulatory networks governing AC identity, which may provide insights into the etiology of congenital heart defects and improve bioengineering strategies.

## MATERIALS AND METHODS

### Ethics statement

All zebrafish husbandry and experiments were performed in accordance with protocols approved by the Cincinnati Children's Hospital Medical Center (CCHMC) Institutional Animal Care and Use Committee (IACUC).

### Zebrafish lines

Adult zebrafish were raised and maintained under standard laboratory conditions (Westerfield, 2000). Transgenic lines used were: *Tg(-5.1myl7:DsRed2-NLS)^f2^* (Mably et al., 2003), *SqET33-mi59B* (Poon et al., 2016), *TgBAC(tcf21:NLS-EGFP)^pd41^* (Wang et al., 2011), *Tg(tcf21:H2A-mCherry)^pd252^* (Cao et al., 2017), *Tg(myh6:Cre^ERT2^*)*^sd20^* (Zhang et al., 2013), *Tg(myl7:CSY)^fb2^* (Zhou et al., 2011), and *Tg(myl7:jGCaMP7c)^ci1031^* and *Tg1(myl7:mCherry)^sd7^* (Palencia-Desai et al., 2011). Mutant alleles used were: *nr2f1a^ci1009^* (Duong et al., 2018), *isl1^ci1032^* and *nr2f1a*;*isl1^ci1033^*. Wild-type lines were a mixed AB/TL background. For all experiments, embryos were grown in 0.003% 1-phenyl-2-thiourea (PTU) to prevent pigmentation.

### Generation of *isl1* and *nr2f1a;isl1* mutant alleles

The *isl1* mutant and *nr2f1a;isl1* linked mutant alleles were generated using CRISPR/Cas9 and processes that have been described previously (Gagnon et al., 2014; Hruscha et al., 2013; Talbot and Amacher, 2014). ChopChop (https://chopchop.cbu.uib.no) was used to select guides for *isl1*. We used a multiplex guide RNA (gRNA) approach with two gRNAs: one targeted the 3′ end of exon 3 and the other targeted the 5′ end of exon 4 of *isl1*. gRNAs were templated using PCR, as previously described (Hruscha et al., 2013), and synthesized using a MEGAshortscript T7 Transcription Kit (Life Technologies, AM1354).

To generate the *isl1* mutant allele, 150 pg of each gRNA along with 6 μM EnGen Spy Cas9-NLS (New England Biolabs, M0646M) was injected into one-cell stage wild-type embryos. F1 progeny from the F0 fish were screened for deletions using PCR. We recovered an allele with a 1622 bp deletion that spans the 3′ end of exon 3, the entirety of the introns 3-4, and the 5′ end of exon 4, which results in a premature stop codon. Embryos homozygous for this deletion phenocopy the previously published *isl1* mutant allele (de Pater et al., 2009; Tessadori et al., 2012), supporting it being a loss-of-function mutation.

To generate the *nr2f1a;isl1* mutant allele, the aforementioned gRNA pair was injected into one-cell stage embryos resulting from a cross of *nr2f1a* heterozygous adults using the specifications above. Adult F0 zebrafish were first genotyped to select for fish heterozygous for the *nr2f1a* mutant allele. F1 progeny from *nr2f1a^+/−^* F0 adults were then screened for *isl1* deletions with PCR. Embryos determined to have both *nr2f1a* and *isl1* mutant alleles were raised to adulthood. The F1 adults were genotyped via PCR from fin clips. Those found to be carrying both *nr2f1a* and *isl1* mutant alleles were then outcrossed. Individual F2 progeny were genotyped with PCR via fin clips to determine if the alleles were on the same chromosome. The *isl1* mutant allele that was recovered on the same chromosome as the *nr2f1a* mutant allele has an identical 1622 bp deletion to our *isl1* mutant allele that was generated independently of *nr2f1a*. Primer and guide information are available in Table S4.

### Knock down using CRISPR/Cas9

To knock down *tcf21*, *wt1a*, *wt1b* and *tbx18*, we used a CRISPR/Cas9 method similar to those shown to recapitulate mutant phenotypes (Hoshijima et al., 2019). Multiple guide RNAs to coding exons within each of the genes were selected using ChopChop (https://chopchop.cbu.uib.no). crRNAs (guide RNAs) for the genes and tracrRNA were ordered from IDT. crRNA:tracrRNA duplexes were prepared according to the manufacturer's descriptions and have been described previously (Hoshijima et al., 2019; Martin et al., 2023). Briefly, to make a 50 μM solution of the crRNA:tracrRNA duplex, equal volumes of 100 μM target-specific crRNA and tracrRNA were mixed together and annealed by heating to 95°C for 5 min, then cooled at 0.1°C/s to 25°C, followed by rapid cooling to 4°C in a PCR machine. An equal volume of duplex buffer (IDT) was then added to further dilute the crRNA:tracrRNA duplex to 25 μM. 1 μl of each 25 μM crRNA:tracrRNA duplex, 0.4 μl of 61 μM Cas9 stock (Alt-R S.p. Cas9 Nuclease V3, IDT), 1.6 μl H_2_O and 1 μl Phenol Red were combined to generate 5 μM of the crRNA:tracrRNA duplex and Cas9 complexes for injection. Prior to injection, the CRISPR/Cas9 complex solution was incubated at 37°C for 5 min and then allowed to rest at room temperature. 1 nl of the 5 μM guide RNA:Cas9 complex was injected into one-cell stage embryos. Efficiency of guide pairs was first assessed using PCR of multiple individual pools of 10 embryos at 24 hpf. Phenotypes generated in injected embryos for efficient guide RNA pairs targeting *wt1a*, *wt1b* and *tcf21* were comparable to published mutants (Boezio et al., 2023; Sanz-Morejón et al., 2019). For all experiments, the efficiency of the selected guide RNA pairs was checked on individual embryos following imaging and/or quantification. Guide RNA and primer sequences are provided in Table S4.

### Generation of *Tg(myl7:jGCaMP7c)* transgenic line

The *Tg(myl7:jGCaMP7c)* transgenic line was generated using standard Gateway/Tol2 methods (Kwan et al., 2007). The jGCaMP7c sequence was amplified from the *pGP-CMV-jGCaMP7c* plasmid (Addgene plasmid #105320; Dana et al., 2019) and cloned into the pDONR221 middle entry vector (Invitrogen). Primers used for subcloning are indicated in Table S4. Gateway cloning was then used to place the *p5E-myl7* 5′ entry clone, *pME-jGCaMP7c* middle-entry clone and *p3E-polyA* 3′ entry clone (Tol2 Kit, plasmid #302; Kwan et al., 2007) into the *pDest-Tol2-P2a;α-crys:DsRed* destination vector (Mandal et al., 2013). Sequences of generated plasmids were confirmed using Sanger sequencing. To generate transgenic embryos, 25 pg of *myl7:jGCaMP7c* plasmid and 25 pg of Tol2 mRNA (Kwan et al., 2007) were co-injected into one-cell stage wild-type embryos. Injected embryos were raised to adulthood, then outcrossed to wild-type fish to identify transgenic founders with *myl7:jGCaMP7c* expression in the heart.

### Immunohistochemistry and cell quantification

Immunohistochemistry (IHC) was performed as previously described (Waxman et al., 2008). Briefly, embryos were fixed in 1% formaldehyde in PBS at room temperature for 1 h, then washed in 1×PBS and 2×0.1% saponin in PBS for 5-10 min at room temperature, and blocked in saponin blocking solution (0.1% saponin, 10% sheep serum and 1×PBS) for 1 h at room temperature. Primary antibodies were diluted in saponin blocking solution and incubated at 4°C overnight. Embryos were then washed in 0.1% saponin in PBS multiple times and incubated with secondary antibodies diluted in saponin blocking solution for 2 h at room temperature. Embryos were washed multiple times with 0.1% saponin in PBS before imaging. Antibody information is provided in Table S5. IHC for quantification of AVC cardiomyocytes required the sequential use of two rabbit primary antibodies: anti-DsRed (Clontech; 632496) and anti-Myh7 (Song et al., 2019). As described previously (Martin et al., 2023), embryos were first stained, as described above, for anti-Myh6 (S46) and anti-DsRed using secondary antibodies anti-mouse IgG1-Dylight405 (BioLegend, 409109; 1:250) and anti-rabbit-TRITC (Southern Biotech, 4050-03; 1:100). After washing off the secondary antibody, embryos were then incubated in saponin blocking solution for 1 h, followed by incubation with the anti-Myh7 primary antibody (YenZym; Song et al., 2019; 1:250) overnight at 4°C. Embryos were then washed with 0.1% saponin in PBS multiple times before being incubated with the anti-rabbit-Alexa-488 secondary antibody (Invitrogen, A11008; 1:500) for 1 h at room temperature. After which, they were washed with 0.1% saponin in PBS before imaging.

Embryos were imaged on a Nikon A1R inverted confocal microscope using either a 20× water immersion or a 60× water immersion objective. Nikon's Denoise-AI was employed on images taken using an HD resonance scanner. Cardiomyocytes were quantified using ImageJ. For quantification of AVC cardiomyocytes: *myl7*:DsRed2-NLS^+^/Myh6^+^/Myh7^−^ cardiomyocytes were counted as ACs, *myl7*:DsRed2-NLS^+^/Myh6^+^/Myh7^+^ cardiomyocytes were counted as AVC and *myl7*:DsRed2-NLS^+^/Myh6^−^/Myh7^+^ cardiomyocytes were counted as VCs. For quantification of ACs and VCs in *isl1* mutants: *myl7*:DsRed2-NLS^+^/Myh6^+^ cardiomyocytes were counted as ACs and *myl7*:DsRed2-NLS^+^/Myh6^−^ were counted as VCs. For all experiments, embryos were genotyped following imaging and/or quantification. We did not notice phenotypic differences between fish that were homozygous for wild-type alleles or heterozygous for the *isl1* or *nr2f1a*;*isl1* mutant alleles. Therefore, both were used as controls and are indicated as WT in figures.

### Hybridization chain reaction *in situ*

Hybridization chain reaction (HCR) was performed as has been previously described (Munjal et al., 2021). HCR probes for *myl7*, *myh6*, *wt1a* and *tbx18* were designed as previously described (Munjal et al., 2021). HCR Amplifiers (v3.0), probe hybridization, probe wash and amplifier buffers were from Molecular Instruments. Embryos from an incross of *nr2f1a^+/−^;isl1^+/−^* fish were grown in embryo water. At 96 hpf, embryos were fixed in 4% PFA overnight at 4°C. After fixation, they were washed three times in PBST followed by dehydration in a methanol series and stored in 100% methanol overnight at −20°C. Embryos were then rehydrated in a methanol series and washed twice with 100% PBST. Embryos were permeabilized in 30 μg/ml proteinase K for 40 min at room temperature, washed three times with PBST, postfixed with 4% PFA for 20 min and then washed five times with PBST. Embryos were then transferred to 1.5 ml tubes, 500 μl of probe hybridization buffer (Molecular Instruments) was added and the embryos were incubated at 37°C for 1 h. Probes were diluted in probe hybridization buffer to 300 pmol to make the probe solution. 200 μl of prewarmed probe solution was added and embryos were incubated at 37°C for 2 days. Embryos were first washed four times in prewarmed probe wash buffer (Molecular Instruments) for 15 min each at 37°C followed by two washes with 5×sodium chloride/sodium citrate/0.1% Tween (SSCT) at room temperature. Samples were incubated for 1 h at room temperature in 500 μl of amplification buffer (Molecular Instruments). Amplifier hairpins were prepared by incubating aliquots at 95°C for 90 s and then allowed to cool to room temperature in the dark for 45 min, as per manufacturer's instructions. Prepared hairpins were diluted 1:50 in probe amplification buffer. 100 μl of diluted hairpins was added to embryos followed by incubation at room temperature in the dark for 2 days. Embryos were then washed five times with 5×SSCT and imaged on a Nikon A1R inverted confocal microscope using a 60× water immersion objective. Nikon's Denoise-AI was employed on images taken using a HD resonance scanner.

### Isolation of cells for single-cell transcriptomic analysis

Hearts were isolated from wild-type, *nr2f1a^−/−^*, *isl1^−/−^* and *nr2f1a^−/−^;isl1^−/−^* embryos carrying the *myl7:mCherry* transgene as described previously (Lombardo et al., 2015). 300 wild-type embryos, 230 *nr2f1a^−/−^* embryos, 300 *isl1^−/−^* embryos and 250 *nr2f1a^−/−^;isl1^−/−^* embryos carrying the *myl7:mCherry* transgene were collected for the isolation of hearts. At 96 hpf, the embryos were anesthetized in 0.16 mg/ml tricaine and transferred to 1.5 ml tubes (∼100 embryos/tube). The embryos were then washed once with 1 ml embryo dissociation media (EDM, L-15 media+10% FBS) and maintained on ice. After adding 1 ml fresh EDM, embryos were gently triturated with a pipette tip until they were mostly dissociated and dissected hearts could be visualized via a microscope. The dissociated embryos were then applied to a 100 μm filter (Falcon; 352360) that had been placed on a 50 ml conical tube, such that isolated hearts would flow through the filter into the tube. The filter was then washed twice with 1 ml EDM. The flow through was applied to a 40 μm filter (Falcon; 352340) to collect the hearts. The filter was washed twice with 1 ml EDM. The hearts were then washed off the filter by turning the filter upside down on a petri dish coated in 2% agar and washing three times with 1 ml EDM. Using a Zeiss_v12 fluorescent dissecting microscope, mCherry^+^ hearts were then isolated from non-fluorescent debris using forceps and transferred to a 1.5 ml tube with 500 μl EDM on ice.

Approximately 70 intact wild-type hearts, ∼115 intact *nr2f1a^−/−^* hearts, ∼70 intact *isl1^−/−^* hearts and ∼95 intact *nr2f1a^−/−^;isl1^−/−^* hearts were recovered. Isolated hearts were then dissociated into a single-cell suspension, as described previously (Bresciani et al., 2018), but with the following modifications. Isolated hearts were centrifuged at 159 ***g*** for 5 min at 4°C. Supernatant was removed and hearts were washed one with 1×PBS followed by centrifugation at 159 ***g*** for 5 min at 4°C. Hearts were then suspended in a solution of 440 μl trypsin (Thermo Fisher; 15400054) and 60 μl Liberase (Millipore Sigma; 5401020001), and placed in a 30°C heat block and triturated with a pipette every 2 min until hearts were dissociated (∼15 min). After the hearts were dissociated, 500 μl of EDM was added to stop the dissociation, followed by centrifugation at 497 ***g*** for 5 min at 4°C. Supernatant was removed and cells were resuspended in 1 ml 1×PBS+1% FBS. Cells were centrifuged at 497 ***g*** at 4° for 5 min, resuspended in 1 ml 1×PBS+1% FBS, and applied to a 40 μm filter (pluriSelect; 43-10040-60) in a 1.5 ml tube. Single cells were concentrated by centrifugation at 497 ***g*** at 4°C for 5 min and then resuspended in 50 μl 1×PBS+1% FBS. This single-cell preparation was submitted to the CCHMC Gene Expression Core and sequenced using the 10x Genomics Chromium platform.

### Single-cell transcriptomic analysis

Reads were processed by Cell Ranger v.7.2.0 using the zebrafish reference genome GRCz11 to generate a gene expression matrix, and the Seurat scRNA-seq analysis workflow (Hao et al., 2021) was then run. Briefly, after generating the gene expression matrix, cells from all samples were merged, and those cells with the number of reads lower than 1000 or higher than 50,000, fewer than 300 genes, more than 30% mitochondrial reads and more than 1% of reads mapped to hemoglobin genes were excluded from the downstream analysis. This resulted in 10,015 wild-type, 9709 *nr2f1a* mutant, 6343 *isl1* mutant, and 9707 *nr2f1a;isl1* mutant cells that passed quality control and were used for further analysis. Clustering analysis was carried out using FindClusters function with the resolution parameter set to 0.3. UMAP dimension reduction was generated using RunUMAP function with the top 20 principal components. Cluster markers were found using FindAllMarker function, keeping only genes whose expression in the analyzed cluster is at least log2(fold-change)>0.5 compared to average expression in other clusters and genes that are expressed in at least 25% of cells in that cluster. Annotations of cell types were carried out by manual inspection of the expression to known markers and corroborated from (Abu Nahia et al., 2024). To find distinct subtypes, ACs and ECs were further reclustered using the workflow mentioned above. To evaluate the similarity between epicardial and atrial subclusters, we quantified the overlap of their respective marker sets using Jaccard statistics. Gene ontology (GO) analysis was performed using the Gene Ontology Resource (Ashburner et al., 2000; Gene Ontology Consortium et al., 2023). scRNA-seq data have been deposited in GEO under accession number GSE319970.

### Live imaging

To analyze heart rate and arrhythmias, wild-type and *nr2f1a;isl1* mutant embryos carrying the *Tg(myl7:jGCaMP7c)* transgene were anesthetized in 0.16 mg/ml tricaine and mounted in 1% low melt agarose. Hearts were imaged on a Nikon Ti-2 SpectraX Widefield microscope with an Andor Xyla 4.2 megapixel, 16-bit sCMOS monochromatic camera at 50-100 frames per second (fps) in a temperature-controlled chamber set to 28.5°C. Analysis was performed in Nikon Elements Software.

### Lineage tracing

To track ACs, we crossed *nr2f1a^+/−^;isl1^+/−^;Tg(myl7:CSY)* zebrafish to *nr2f1a^+/−^;isl1^+/−^;Tg(myh6:Cre^ERT2^)* zebrafish. At shield stage, embryos were placed into glass vials (30 embryos/vial) and treated with 10 μM 4-hydroxytamoxifen (4-HT; Sigma, H7904) until 48 hpf. After 48 hpf, the 4-HT was washed out and the embryos were returned to petri dishes to develop until 96 or 120 hpf when they were collected for IHC using the saponin protocol described above.

### Statistical analysis

Comparisons between groups were analyzed using Welch's *t*-test and two-way ANOVAs with multiple comparisons as appropriate. Fisher's exact test was used to determine if two proportions were statistically distinct. Statistical analysis was performed using GraphPad Prism. *P*<0.05 was considered statistically significant for all analysis. Error bars in all graphs indicate the standard error of the mean (s.e.m.).

## Supplementary Material



10.1242/develop.205396_sup1 Supplementary information

Table S1. Gene enrichment for clusters in hearts at 96 hpf.

Table S2. Gene enrichment in atrial cardiomyocyte clusters.

Table S3. Gene enrichment in epicardial cell clusters.

Table S4. Guide RNA and primer sequences.

Table S5. Primary and secondary antibody information.
