# Supplementary material for: Nr2f1a and Isl1 repress acquisition of epicardial identity in venous atrial cardiomyocytes
Source: Development. 2026 Jun 4;153(11):dev205396. doi: 10.1242/dev.205396 (PMC13286355; doi:10.1242/dev.205396)
Supplement: Supplementary information [file develop-153-205396-s1.pdf]

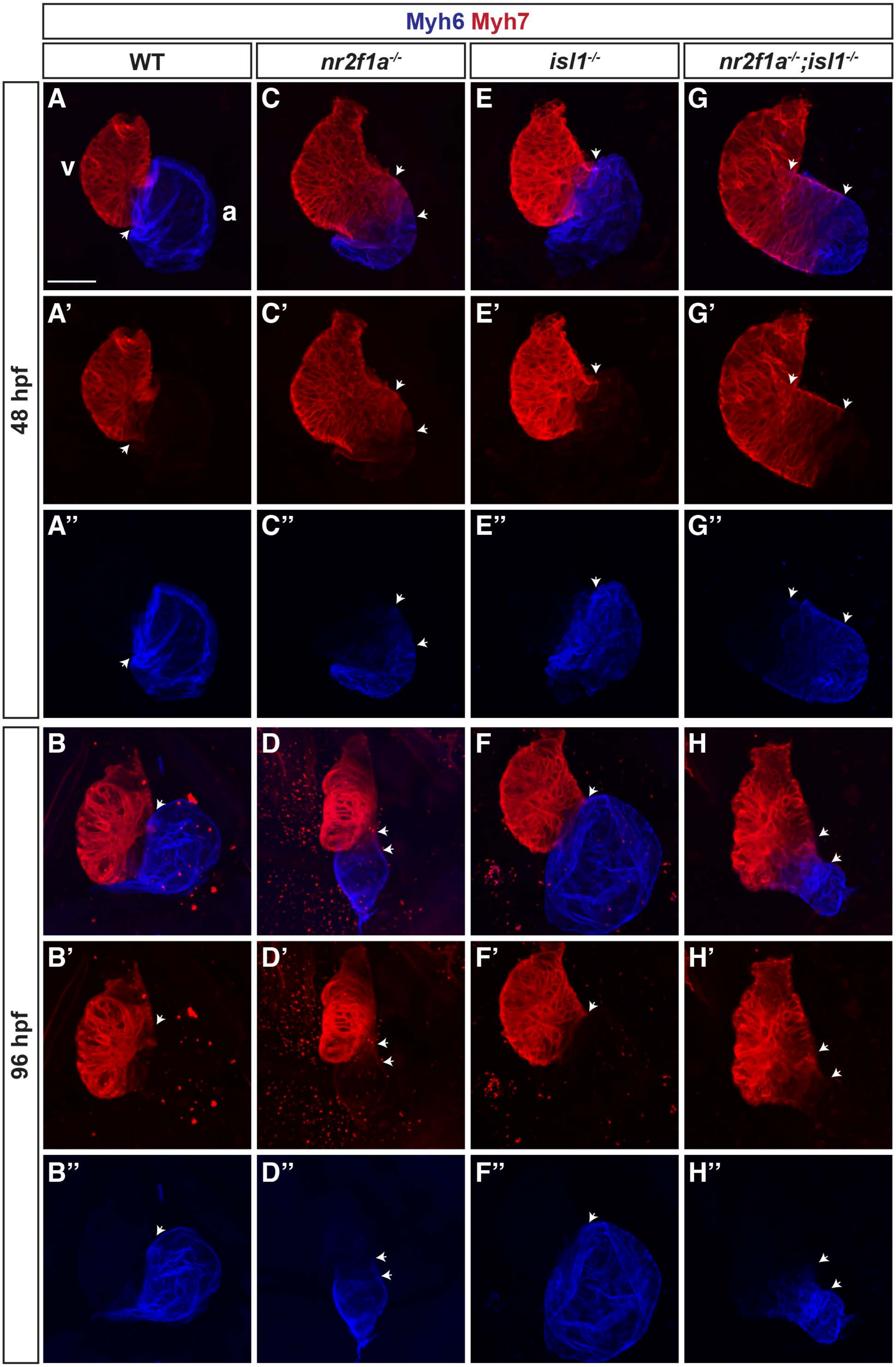
Myh6 Myh7
WT
nr2f1a-/-
isl1-/-
nr2f1a-/-;isl1-/-
48 hpf
96 hpf
A
C
E
G
v
a
A'
C'
E'
G'
A''
C''
E''
G''
B
D
F
H
B'
D'
F'
H'
B''
D''
F''
H''

Fig. S1. Myh6 and Myh7 expression in hearts of WT, *nr2f1a* mutant, *isl1* mutant, and *nr2f1a;isl1* mutant embryos. A-H'') Overlay and individual channels showing IHC for Myh6 (blue) and Myh7 (red) in WT, *nr2f1a*$^{-/-}$, *isl1*$^{-/-}$, and *nr2f1a*$^{-/-}$;*isl1*$^{-/-}$ hearts from **Fig. 1D-K**. Overlapping expression of Myh6 and Myh7 in the AVC (white arrows). Scale bar: 25 µm. 48 hpf: WT (n = 11), *nr2f1a*$^{-/-}$ (n = 4), *isl1*$^{-/-}$ (n = 13), *nr2f1a*$^{-/-}$;*isl1*$^{-/-}$ (n = 7); 96 hpf: WT (n = 16), *nr2f1a*$^{-/-}$ (n = 11), *isl1*$^{-/-}$ (n = 8), *nr2f1a*$^{-/-}$;*isl1*$^{-/-}$ (n = 8).

Development • Supplementary information

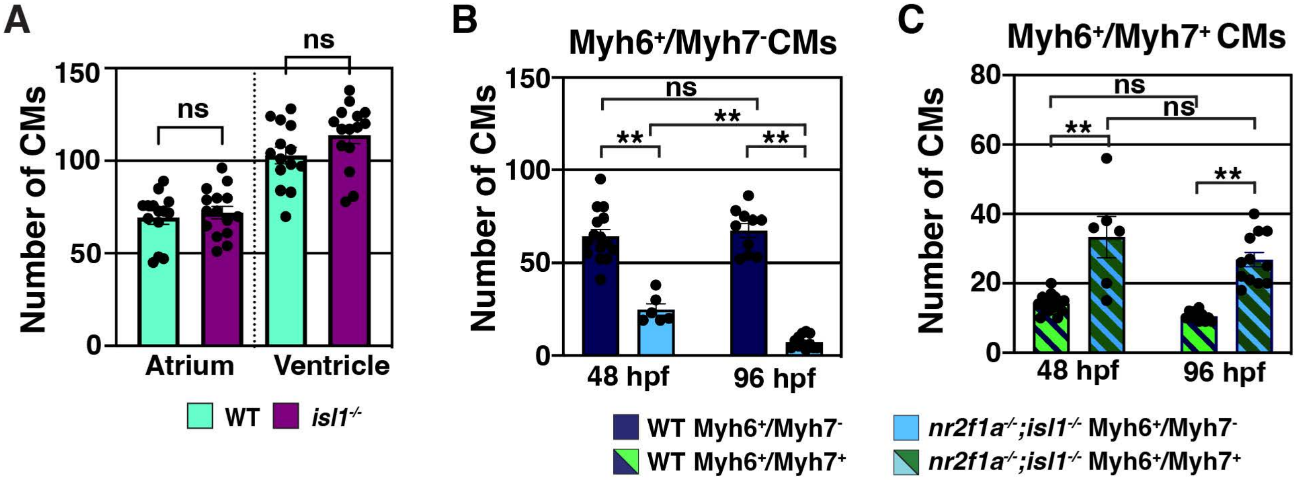

Fig. S2. *Nr2f1a;isl1* mutants have a progressive loss of Myh6⁺ cardiomyocytes. A) Quantification of cardiomyocytes (CMs) in the atria and ventricles of *isl1* mutants. Atrium indicates Myh6$^+$/Myh7$^-$ CMs. Ventricle indicates Myh6$^-$/Myh7$^+$ CMs. WT (n = 14); isl1$^{-/-}$ (n = 15). Not significant (ns) using Welch's t-test. **B,C)** Quantification of Myh6$^+$/Myh7$^-$ (Myh6$^+$-only) CMs and Myh6$^+$/Myh7$^+$ CMs in WT and *nr2f1a;isl1* mutants. 48 hpf: WT (n = 15), *nr2f1a$^{-/-}$;isl1$^{-/-}$* (n = 6); 96 hpf: WT (n = 10), *nr2f1a$^{-/-}$;isl1$^{-/-}$* (n = 12). ** P < 0.001 (using one-way ANOVA with multiple comparisons).

Development • Supplementary information

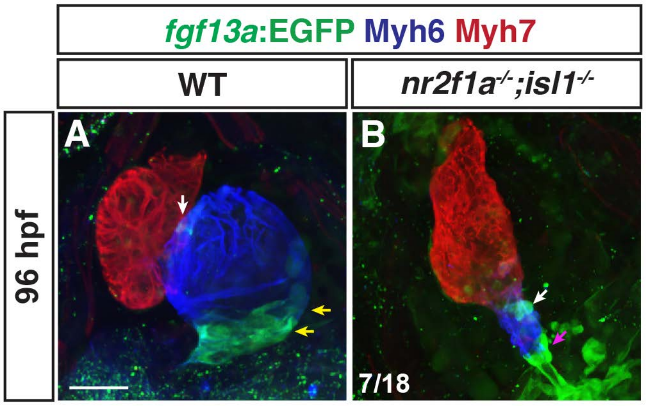

Fig. S3. *Nr2f1a;isl1* mutants lack PCs. A,B) IHC for *fgf13a*:EGFP (green), Myh6 (blue), and Myh7 (red) in WT and *nr2f1a*$^{-/-}$*;isl1*$^{-/-}$ hearts at 96 hpf. The limits of *fgf13a*:EGFP expression within the WT heart (yellow arrow). *Fgf13a*:EGFP at the venous pole that does not overlap with Myh6 (magenta arrow). AV node (white arrows). The 96 hpf WT heart in A is also presented in Fig. 2B for comparison to the phenotypes observed in *nr2f1a*$^{-/-}$, *isl1*$^{-/-}$, and the majority of *nr2f1a*$^{-/-}$*;isl1*$^{-/-}$ hearts (11/18). In **B**, 7 of 18 embryos from a representative clutch showed a similar phenotype. Scale bar: 25 μm. WT (n = 13), *nr2f1a*$^{-/-}$*;isl1*$^{-/-}$ (n = 18).

Development • Supplementary information

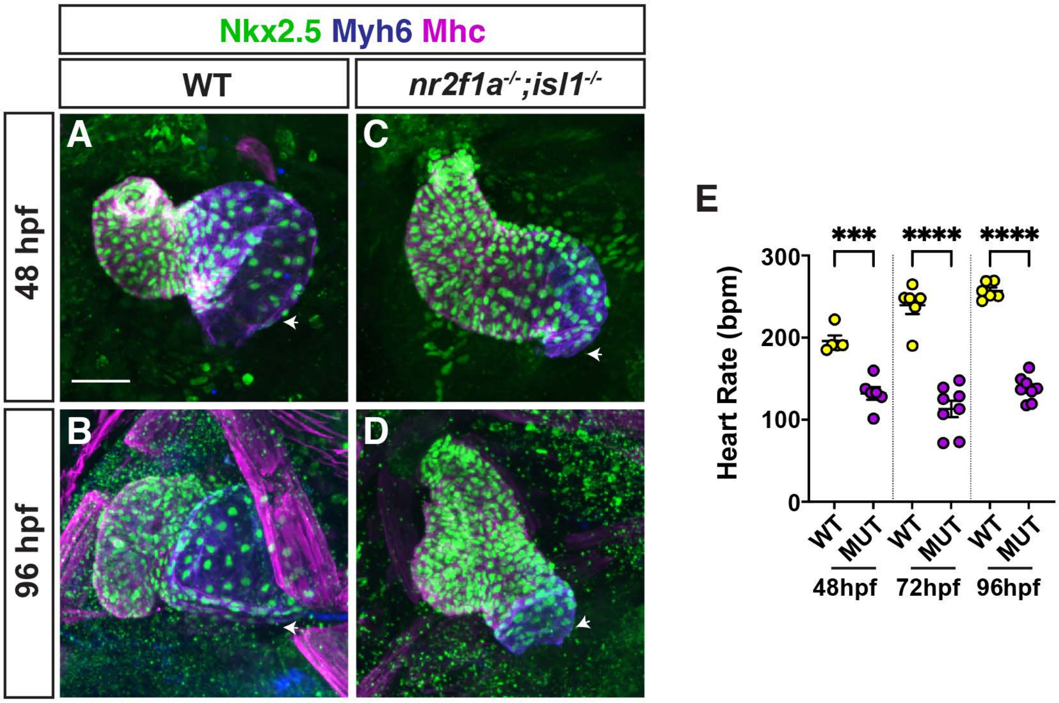

Fig. S4. Nkx2.5 expression and heart rate in *nr2f1a;isl1* mutants. A-D) IHC for Nkx2.5 (green), Mhc (magenta), and Myh6 (blue) in WT and *nr2f1a*$^{-/-}$*;isl1*$^{-/-}$ hearts. Border of Nkx2.5^{+} nuclei within the venous pole of the heart (white arrows). Scale bar: 25 µm. 48 hpf: WT (n = 8), *nr2f1a*$^{-/-}$*;isl1*$^{-/-}$ (n = 7); 96 hpf: WT (n = 10), *nr2f1a*$^{-/-}$*;isl1*$^{-/-}$ (n = 5). **E)** Quantification of heart rates in WT sibling and *nr2f1a*$^{-/-}$*;isl1*$^{-/-}$ embryos at 48 through 96 hpf. 48 hpf: WT (n = 5), *nr2f1a*$^{-/-}$*;isl1*$^{-/-}$ (n = 6); 72 hpf: WT (n = 6), *nr2f1a*$^{-/-}$*;isl1*$^{-/-}$ (n = 8); 96 hpf: WT (n = 6), *nr2f1a*$^{-/-}$*;isl1*$^{-/-}$ (n = 8). *** $P = 0.0002$; **** $P < 0.0001$ (using ANOVA with multiple comparisons).

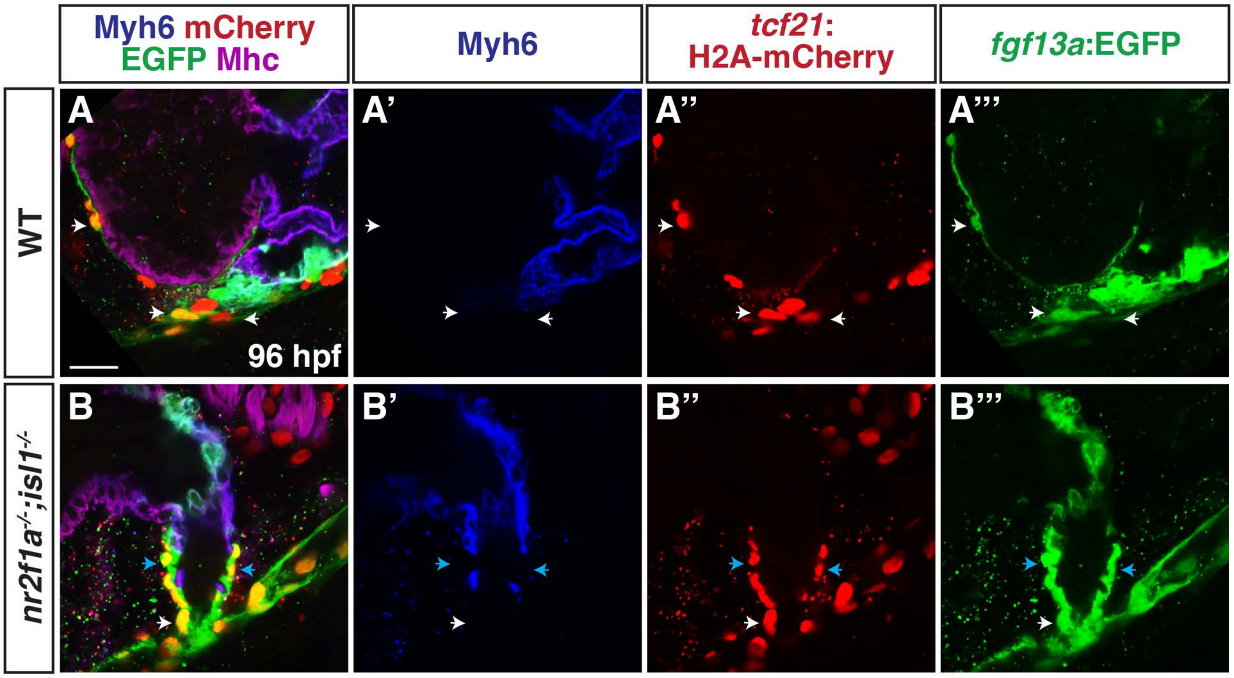

Fig. S5. *Fgf13a:EGFP* is expressed in ECs. A-B''') Optical sections of IHC for *fgf13a*:EGFP (green), *tcf21*:H2A-mCherry (red), Mhc (magenta), and Myh6 (blue) in WT and *nr2f1a*$^{-/-}$;*isl1*$^{-/-}$ hearts at 96 hpf. Overlapping *fgf13a*:EGFP and *tcf21*:H2A-mCherry expression in the epicardium at the venous pole and ventricle (white arrows). Myh6^{-}/*fgf13a*:EGFP^{+}/*tcf21*:H2A-mCherry^{+} cells in *nr2f1a*$^{-/-}$;*isl1*$^{-/-}$ hearts (blue arrows). Scale bar: 10 μm. WT (n = 14), *nr2f1a*$^{-/-}$;*isl1*$^{-/-}$ (n = 5).

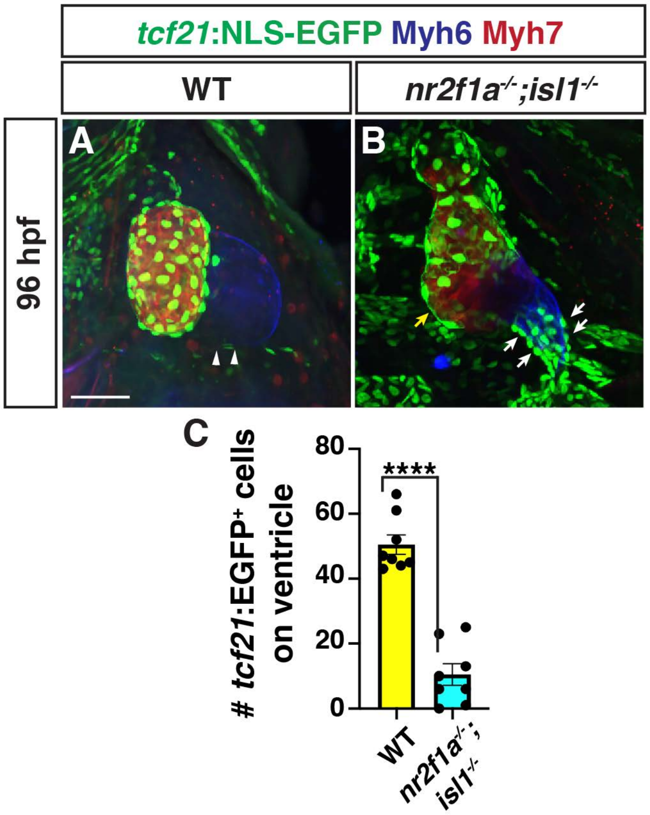

Fig. S6. *Tcf21:NLS-EGFP* expression in *nr2f1a;isl1* mutants. A,B) Confocal images of IHC for *tcf21*:NLS-EGFP (green), Myh7 (red), and Myh6 (blue) in WT and *nr2f1a*$^{-/-}$;*isl1*$^{-/-}$ hearts at 96 hpf. In **A**, *tcf21*:NLS-EGFP^{+} cells adjacent to the venous pole (white arrowheads). In **B**, *tcf21*:NLS-EGFP^{+} cells on and adjacent to the atrium of the *nr2f1a*$^{-/-}$;*isl1*$^{-/-}$ embryo (white arrows). In **B**, the reduced nuclei and more sparse coverage of the ECs on the ventricle of *nr2f1a*$^{-/-}$;*isl1*$^{-/-}$ embryo (yellow arrow). Scale bar: 25 µm. WT (n = 8), *nr2f1a*$^{-/-}$;*isl1*$^{-/-}$ (n = 8). C) Quantification of *tcf21*:NLS-EGFP^{+} ECs on the ventricles of WT and *nr2f1a*$^{-/-}$;*isl1*$^{-/-}$ mutants. **** $P < 0.0001$ (using two-tailed Welch's t-test).

Development • Supplementary information

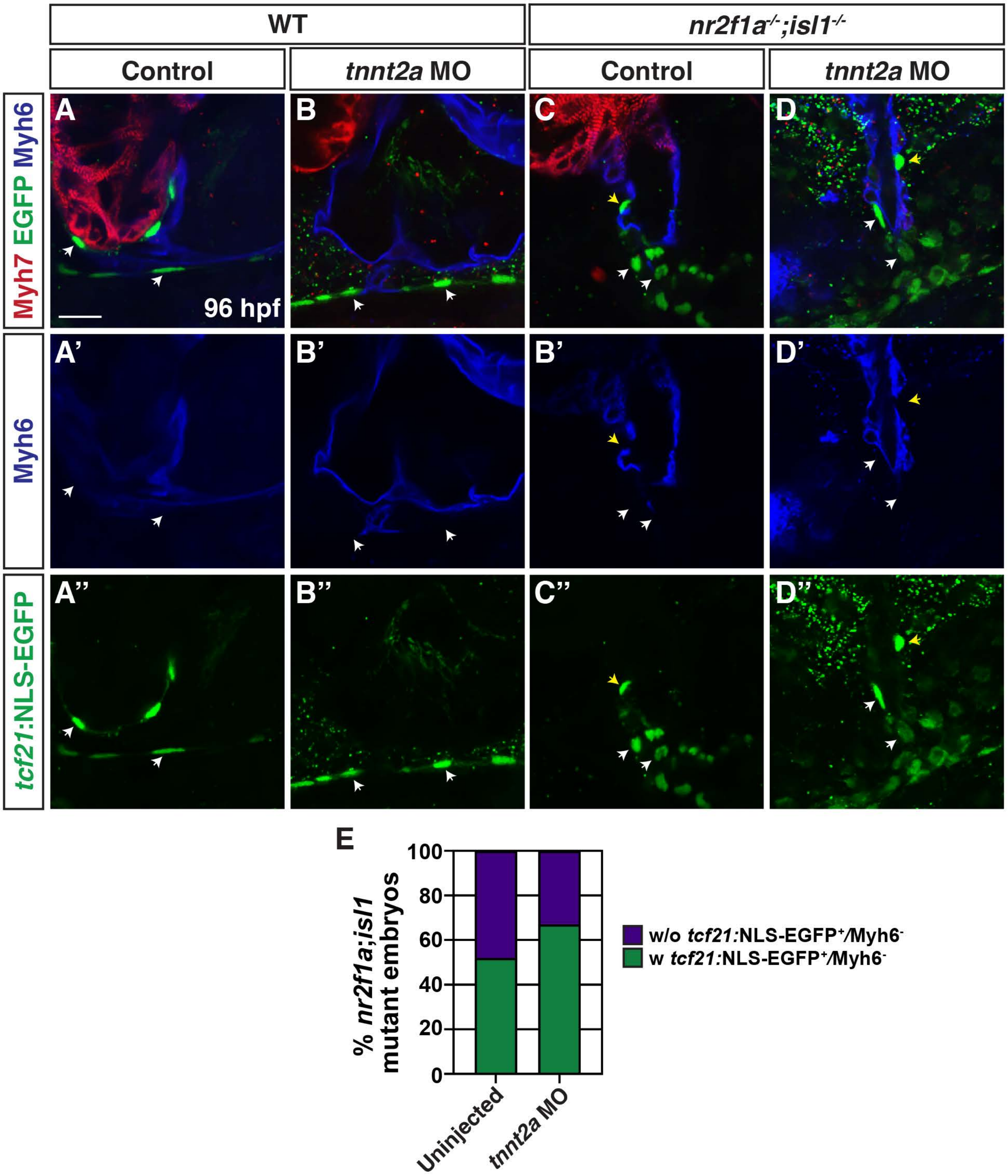

Fig. S7. Cardiac contractions do not significantly affect ECs within the myocardial layer. A-D'') Optical sections of IHC for *tcf21*:NLS-EGFP (green), Myh7 (red), and Myh6 (blue) in heart of control and *tnnt2a* MO-injected WT and *nr2f1a*$^{-/-}$;*isl1*$^{-/-}$ embryos at 96 hpf. *Tcf21*:NLS-EGFP^{+} cells on the ventricle (in WT) and adjacent to the venous pole (white arrows). *Tcf21*:NLS-EGFP^{+}/Myh6^{-} cells in the atrial myocardial wall (yellow arrows). Scale bar: 10 µm. Uninjected: WT (n = 16), *nr2f1a*$^{-/-}$;*isl1*$^{-/-}$ (n = 23); *tnnt2a* MO: WT (n = 39), *nr2f1a*$^{-/-}$;*isl1*$^{-/-}$ (n = 18). **E)** Percentage of control and *tnnt2a* MO-injected *nr2f1a*$^{-/-}$;*isl1*$^{-/-}$ embryos with and without *tcf21*:NLS-EGFP^{+}/Myh6^{-} cells within the atrial wall. Uninjected *nr2f1a*$^{-/-}$;*isl1*$^{-/-}$ (n = 23); *tnnt2a* MO-injected *nr2f1a*$^{-/-}$;*isl1*$^{-/-}$ (n = 18). Not significant using using Fisher's exact test.

Development • Supplementary information

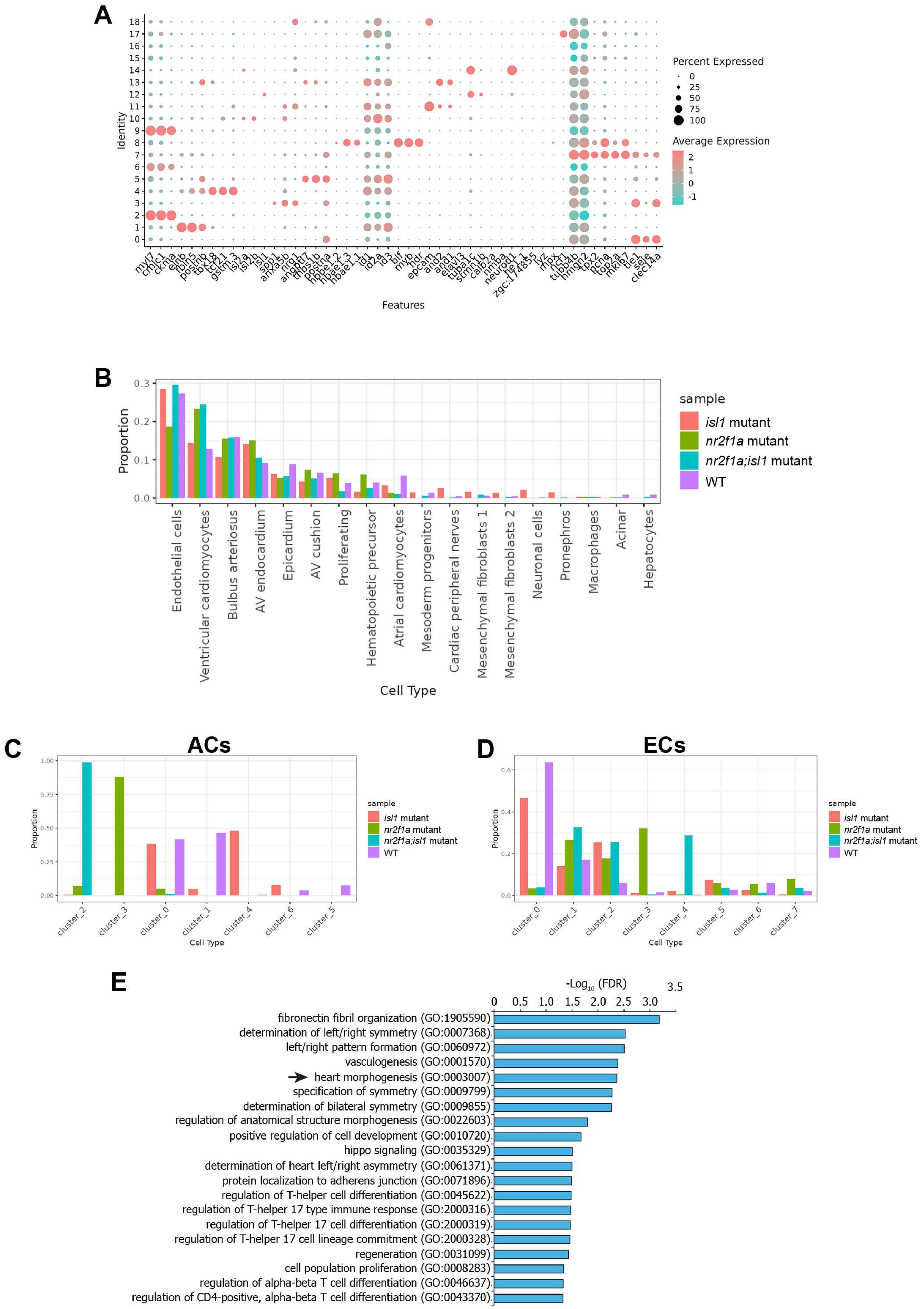

Development • Supplementary information

Fig. S8. Marker, cluster contributions, and GO analysis of 96 hpf hearts. A) Marker gene expression in clusters from the single cell transcriptomics of the 96 hpf WT, *isl1* mutant, *nr2f1a* mutant, and *nr2f1a;isl1* mutant hearts. **B)** Proportion of cells from each condition in the different cell clusters. C) Proportion of cells from each condition in the AC subclusters. **D)** Proportion of cells from each condition in the EC subclusters. **E)** GO analysis of the EC cluster 4.

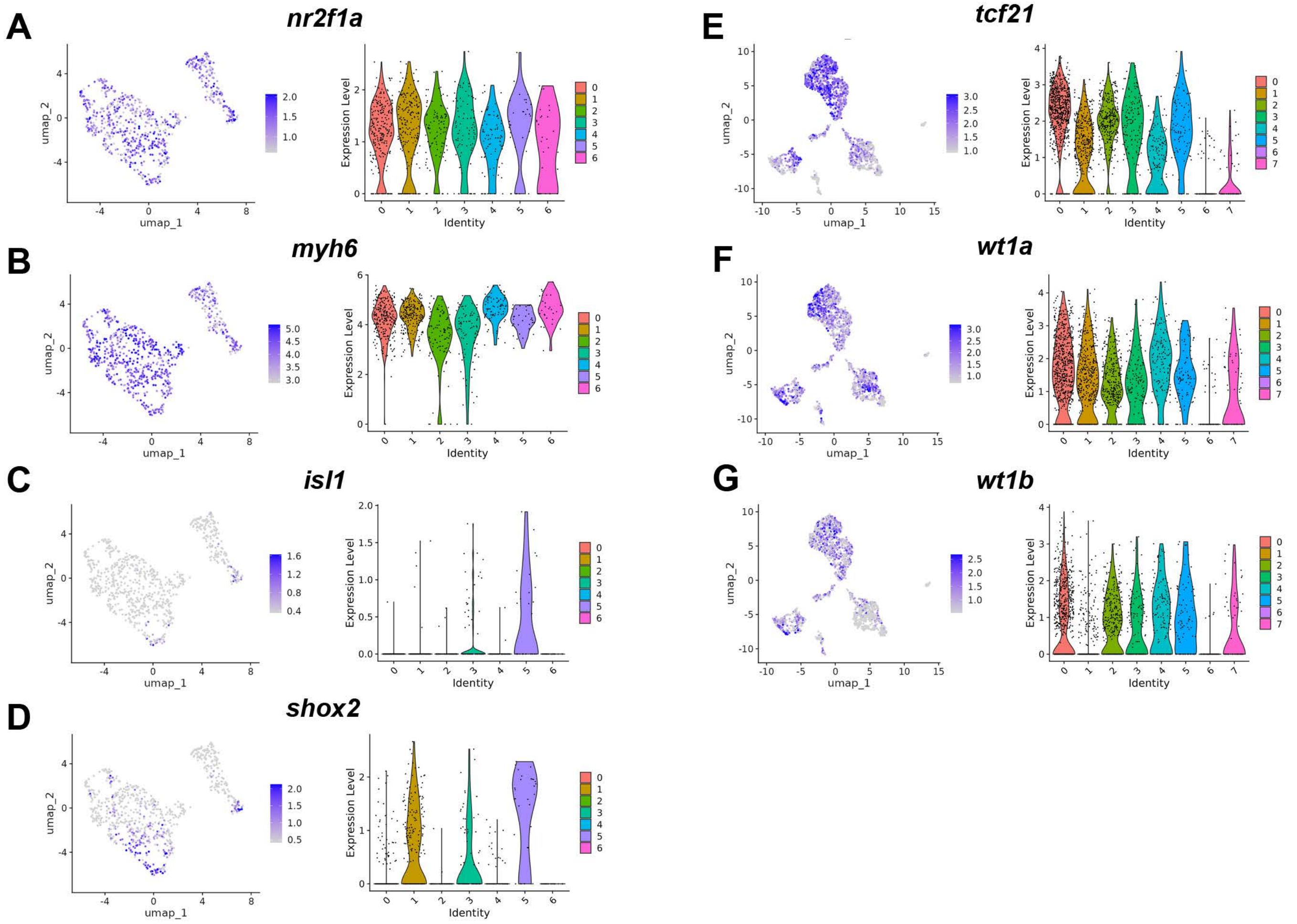

Fig. S9. Marker gene expression within the AC and EC subclusters. A-D) UMAPs (left) and violin plots (right) of the AC subclusters displaying expression of *nr2f1a*, *myh6*, *isl1*, and *shox2*. **E-G)** UMAPs (left) and violins plots (right) of the EC subclusters displaying expression of *tcf21*, *wt1a*, and *wt1b*.

Development • Supplementary information

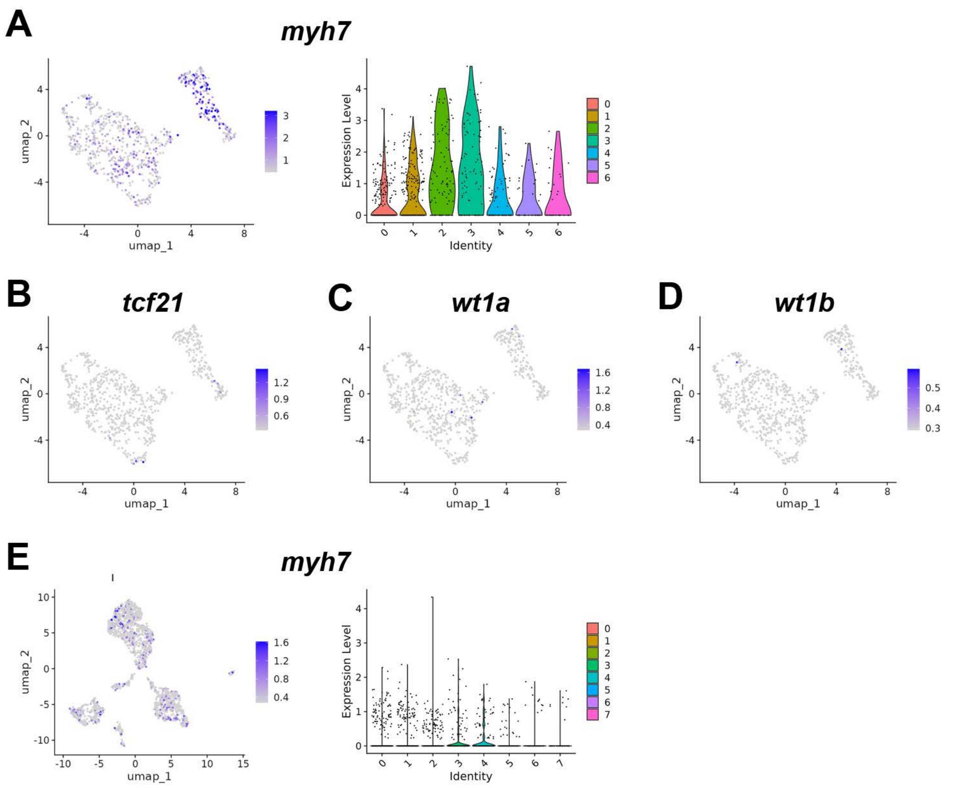

Fig. S10. Marker gene expression within AC and EC subclusters. A) UMAP (left) and violin plot (right) of *myh7* expression in the AC subclusters. **B-D)** UMAPs showing lack of *tcf21*, *wt1a*, and *wt1b* expression in AC subclusters. **E)** UMAP (left) and violin plot (right) of *myh7* expression in the EC subclusters.

Development • Supplementary information

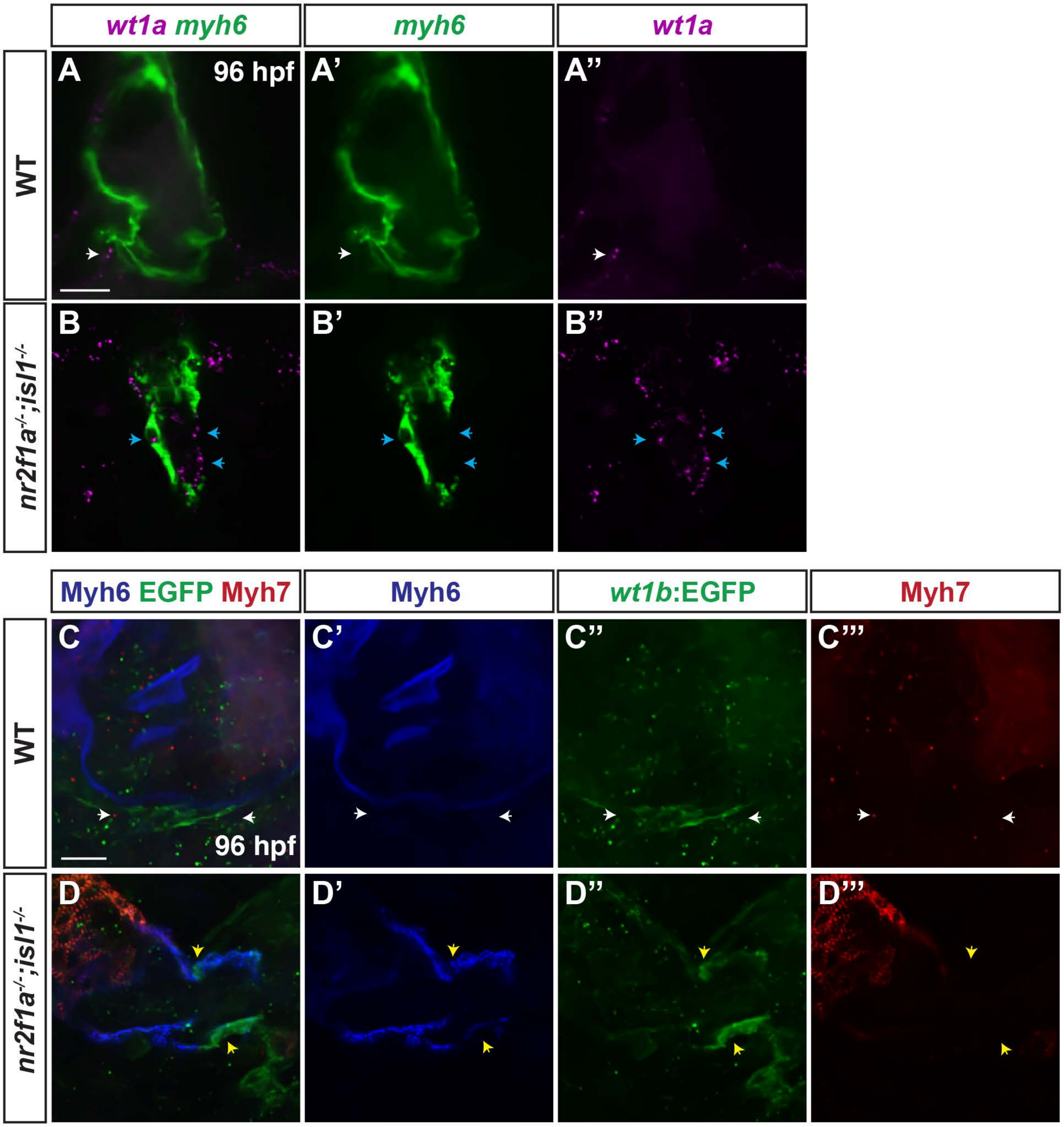

Fig. S11. *Wt1a* and *wt1b* expression in the atrial myocardium of *nr2f1a;isl1* mutant hearts. A-B'') Optical sections of hybridization chain reaction (HCR) in situ for *wt1a* and *myh6* in WT sibling and *nr2f1a*$^{-/-}$;*isl1*$^{-/-}$ embryo hearts at 96 hpf. In **A-A''**, *wt1a* expression at the venous pole in the PEO (white arrows). In **B-B''**, *wt1a* expression in the atrial myocardial layer that lack myh6 expression (blue arrows). WT (n = 3), *nr2f1a*$^{-/-}$;*isl1*$^{-/-}$ (n = 3). **C-D''')** Optical sections of IHC for *wt1b*:EGFP (green), Myh6 (blue), Myh7 (red) in WT sibling and *nr2f1a*$^{-/-}$;*isl1*$^{-/-}$ embryos at 96 hpf. In **C-C'''**, *wt1b*:EGFP expression at the venous pole of the heart (white arrows). In **D-D'''**, indicate *wt1b*:EGFP + cells in the atrial myocardial layer that lack Myh6 (yellow arrows). WT (n = 8), *nr2f1a*$^{-/-}$;*isl1*$^{-/-}$ (n = 8). Scale bars: 10 μm.

Development • Supplementary information

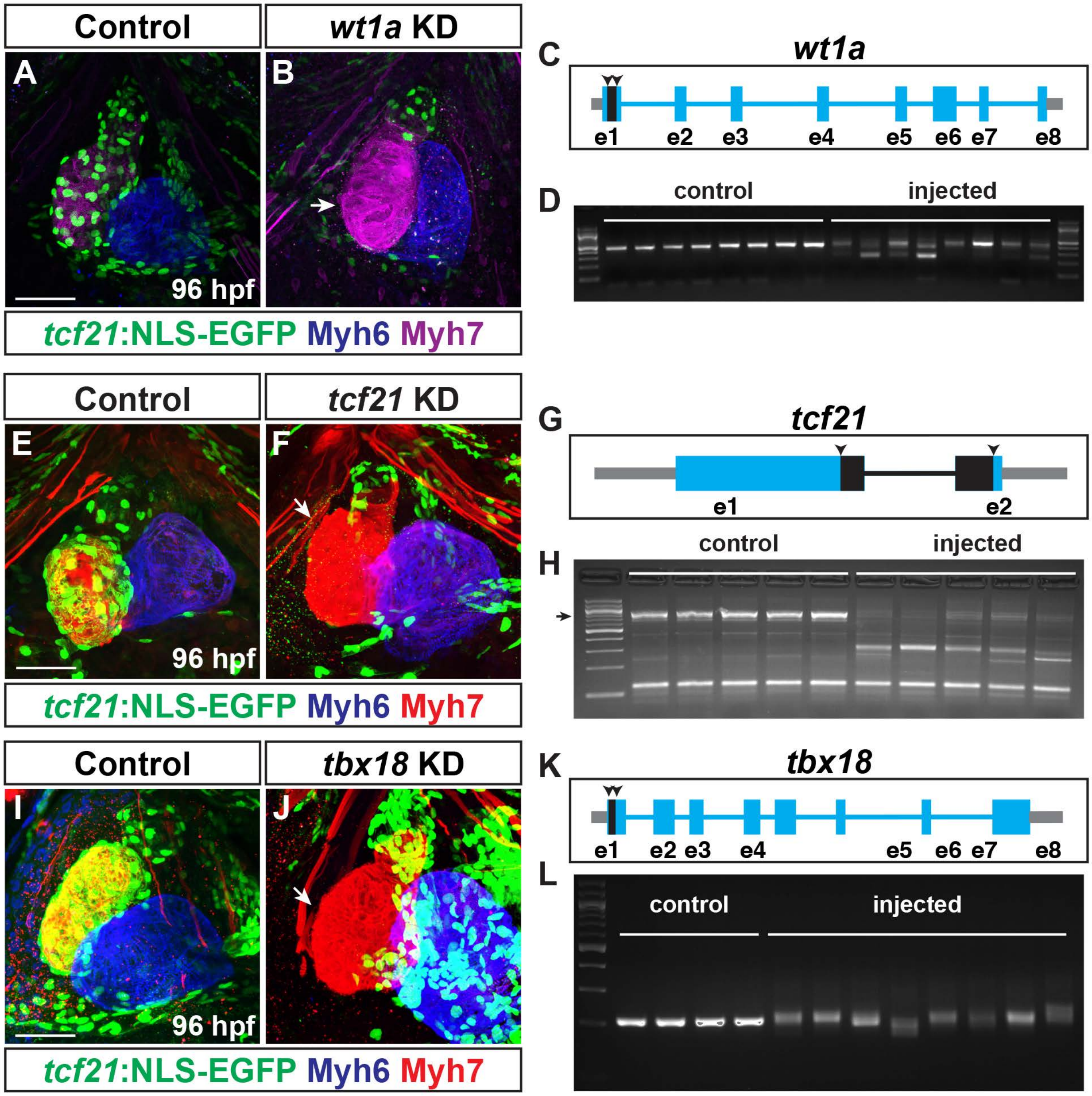

Fig. S12. *Wt1a*, *tcf21*, and *tbx18* KD causes a loss of ECs on the ventricle. A,B) Confocal images of IHC for *tcf21*:NLS-EGFP (green), Myh7 (magenta), and Myh6 (blue) in hearts of control and wt1a knockdown (KD) (CRISPR/Cas9-injected) embryos. Control (n = 8); wt1a KD (n = 8). **C)** Schematic of the *wt1a* locus. Black arrows indicate the location of the *wt1a* guides used. Black between arrows indicates the potential deletion created by co-injection of guides. **D)** Gel indicating PCR for *wt1a* locus in representative individual control and *wt1a* CRISPR/Cas9-injected embryos. **E,F)** Confocal images of IHC for *tcf21*:NLS-EGFP (green), Myh7 (red), and Myh6 (blue) in hearts of control and *tcf21* KD (CRISPR/Cas9-injected) embryos. Control (n = 20); *tcf21* KD (n = 22). **G)** Schematic of the *tcf21* locus. Black arrows indicate the location of the *tcf21* guides used. Black between arrows indicates the potential deletion created by co-injection of guides. H) Gel indicating PCR for *tcf21* locus in representative individual control and *tcf21* CRISPR/Cas9-injected embryos. Black arrow indicates WT band in control embryos. **I,J)** Confocal images of IHC for *tcf21*:NLS-EGFP (green), Myh7 (red), and Myh6 (blue) in hearts of control and *tbx18* KD (CRISPR/Cas9-injected) embryos. Control (n = 4); *tbx18* KD (n = 8). **K)** Schematic of the *tbx18* locus. Black arrows indicate the location of the *tbx18* guides used. Black between arrows indicates the potential deletion created by co-injection of guides. **I)** Gel indicating PCR for *tbx18* locus in representative individual control and *tbx18* CRISPR/Cas9-injected embryos. Scale bars: 25 μm.

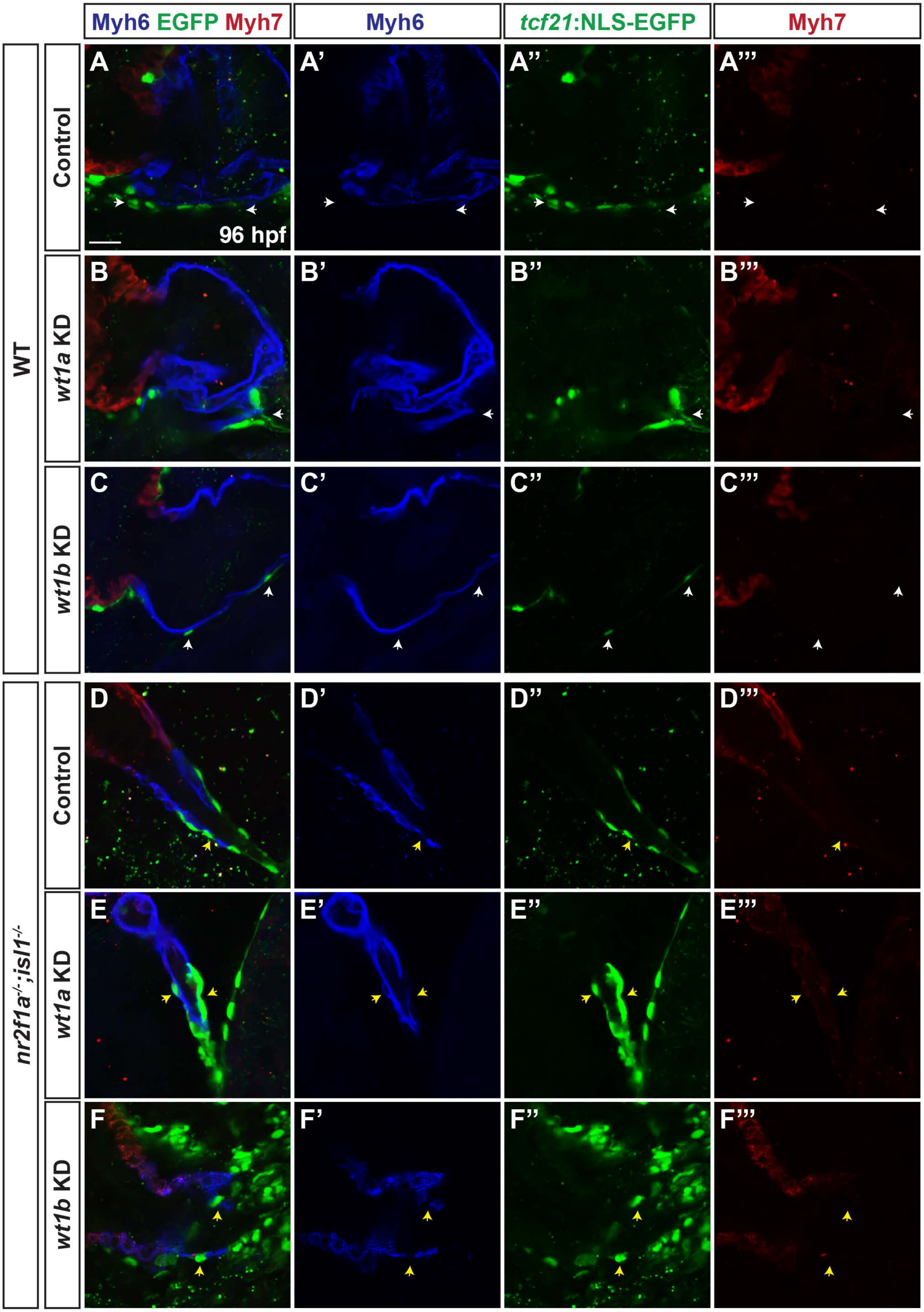
Myh6 EGFP Myh7
Myh6
tcf21:NLS-EGFP
Myh7
WT
Control
wt1a KD
wt1b KD
nr2f1a-/-;isl1-/-
Control
wt1a KD
wt1b KD
A
A’
A”
A’’’
96 hpf
B
B’
B”
B’’’
C
C’
C”
C’’’
D
D’
D”
D’’’
E
E’
E”
E’’’
F
F’
F”
F’’’

Fig. S13. *Wt1a* and *wt1b* are not required to promote EC gene expression in *nr2f1a;isl1* mutant atria. A-F''') Optical sections of IHC for Myh6 (blue), Myh7 (red), and *tcf21*:NLS-EGFP (green) in the hearts of control (uninjected), *wt1a* knockdown (KD), and *wt1b* KD WT and *nr2f1a*$^{-/-}$;*isl1*$^{-/-}$ embryos at 96 hpf. In **A-C'''**, *tcf21*:NLS-EGFP^{+} ECs at the venous pole of the heart (white arrowheads). In **D-F'''**, *tcf21*:NLS-EGFP^{+}/Myh6^{-} cells within the myocardial wall (yellow arrowheads). Scale bar: 10 µm. WT: uninjected (n= 12), *wt1a* KD (n= 26), *wt1b* KD (n= 4); *nr2f1a*$^{-/-}$;*isl1*$^{-/-}$: uninjected (n= 12), *wt1a* KD (n= 12), *wt1b* KD (n = 3).

Table S1. Gene enrichment for clusters in hearts at 96 hpf.

Available for download at
https://journals.biologists.com/dev/article-lookup/doi/10.1242/dev.205396#supplementary-data

Table S2. Gene enrichment in atrial cardiomyocyte clusters.

Available for download at
https://journals.biologists.com/dev/article-lookup/doi/10.1242/dev.205396#supplementary-data

Table S3. Gene enrichment in epicardial cell clusters.

Available for download at
https://journals.biologists.com/dev/article-lookup/doi/10.1242/dev.205396#supplementary-data

Table S4. Guide RNA and primer sequences.

Available for download at
https://journals.biologists.com/dev/article-lookup/doi/10.1242/dev.205396#supplementary-data

Table S5. Primary and secondary antibody information.

Available for download at
https://journals.biologists.com/dev/article-lookup/doi/10.1242/dev.205396#supplementary-data

Development • Supplementary information
