# Supplementary material for: Nr2f1a and Isl1 repress acquisition of epicardial identity in venous atrial cardiomyocytes
Source: Development. 2026 Jun 4;153(11):dev205396. doi: 10.1242/dev.205396 (PMC13286355; doi:10.1242/dev.205396)
Supplement: Supplementary file 1 [file develop-153-205396_review_history.pdf]

Nr2f1a and Isl1 repress acquisition of epicardial identity in venous atrial cardiomyocytes

Kendall E. Martin, Andrew T. Fernandes, Mohammed Sayed, Margaret A. Hanlon, Samuel Fallon, Hee-Woong Lim and Joshua S. Waxman
DOI: 10.1242/dev.205396

Editor: Benoit Bruneau

Review timeline

Original submission:	21 November 2025
Editorial decision:	9 January 2026
First revision received:	28 April 2026
Accepted:	29 April 2026

Original submission

First decision letter

MS ID#: dev.205396

MS TITLE: Nr2f1a and Isl1 repress acquisition of epicardial identity in venous atrial cardiomyocytes

AUTHORS: Kendall E. Martin, Andrew T. Fernandes, Mohammed Sayed, Margaret A. Hanlon, Samuel Fallon, Hee-Woong Lim and Joshua Waxman

Dear Dr Waxman,

I have now received all the referees reports on the above manuscript, and have reached a decision. The referees' comments are appended below.

The overall evaluation is positive and we would like to publish a revised manuscript in Development, provided that the referees' comments can be satisfactorily addressed. Please attend to all of the reviewers' comments in your revised manuscript and detail them in your point-by-point response. If you do not agree with any of their criticisms or suggestions explain clearly why this is so. If it would be helpful, you are welcome to contact us to discuss your revision in greater detail. Please send us a point-by-point response indicating your plans for addressing the referees' comments, and we will look over this and provide further guidance.

Reviewer 1

Advance summary and potential significance to field

In this manuscript, Martin et al. explore the interaction between Nr2f1a and Isl1 in the differentiation of different cell types in the developing zebrafish atrium. They find that Nr2f1a and Isl1 work together to maintain atrial myocardial identity in two stages, first against an alternative differentiation pathway to ventricular and atrioventricular canal cardiomyocytes at the atrial outflow and then against transdifferentiation to epicardial cells at the venous pole. This is a valuable addition to the understanding of gene regulatory networks involved in atrial chamber identity during zebrafish heart development. I recommend publication in Development with minor revisions mainly involving additional analyses.

© 2026. Published by The Company of Biologists under the terms of the Creative Commons Attribution License (https://creativecommons.org/licenses/by/4.0/).

Comments for the author

Fig 1.
1) The partitioning of cells into VC, AVC, and AC identities would be clearer if the number and fraction of each of Myh6+/Myh7+, Myh6+/Myh7-, Myh6-/Myh7+ were simply shown for each of WT, nr2f1a-/- and nr2f1a-/-;isl1-/- on a single panel in Fig. 1. Currently Myh6+/Myh7+ are counted both in Myh6+ and Myh7+ populations, and one needs to look at many different panels to understand how the partitioning of cells changes across the genotypes. I currently see the expansion of AVC identity into the atrium in nr2f1a-/-;isl1-/- vs nr2f1a-/-, but I do not see the support for VC identity also increasing in nr2f1a-/-;isl1-/- vs nr2f1a-/-.
2) The difference in AVC extent between the nr2f1a -/- and nr2f1a-/-;isl1-/- genotypes is not entirely convincing in the representative images F,G,J,K due to variations in morphology and positioning of the heart. Would they be more apparent if separate images were shown for each channel? Could a quantitative analysis be performed for each genotype describing the average Myh6 and Myh7 intensities as a function of distance from the venous pole?
3) How are Myh6+ and Myh7+ calls determined? Is the proportion of cells binned into each of Myh6+/Myh7+, Myh6+/Myh7-, Myh6-/Myh7+ sensitive to the cutoffs chosen for each marker? In other words, are expression differences for each cell type binary (unambiguous positive or negative) or quantitatively graded and is this consistent across all genotypes?

Fig. 2:
4) The Myh6+ status of fgf13a:GFP+ cells in the two groups of double mutant embryos in Fig 2H and Fig S2 is not clear. Are there any fgf13a:GFP+/Myh6+ cells at all in either group as there are in WT and nr2f1a-/- single mutants? If so, how can we be certain that these are not pacemaker cells? The functional data seems insufficient in Fig 3 and S3 - disruption in heart rate and conduction path could happen without complete depletion of pacemaker cells.

Fig. 3:
5) Was motion correction performed for analysis of GCaMP imaging? Alternatively, was analysis done to demonstrate that motion is negligible in the DF/F traces (e.g. by choosing several regions of interest of different sizes and positions within the same sample and showing that the traces are identical)? If not, the durations and shapes of Ca2+ transients are not interpretable and the authors should clearly note this (the experiment is still useful for comparing heart rate and heart rate variability).
6) I suggest the term "intensity trace" as opposed to "kymograph" for the legend of plots B, D, F, H to avoid confusion. Kymographs in modern signal processing commonly refer to a plot with time on one axis, a spatial coordinate on another, and a quantity such as intensity plotted as color (as opposed to the traces generated by an analog "kymograph" instrument).
7) A spatial description of GCaMP activity should be shown (e.g. a modern kymograph, or an activation map) to illustrate what propagation looks like from the primary and secondary pacemakers and support the claim in the text of propagation from AV node. Quantification of the frequency of these events versus normal propagation should be done.

Figs. 4-10
8) Representative images are generally convincing but quantification should be done to support the biological claims, for example:
* Fig 4: fraction of fgf13a:GFP cells that are Myh6+ in WT, nr2f1a-/-, and nr2f1a-/-;isl1-/- genotypes
* Fig 5 A-B: Myh6 intensity in myl7:DsRed2-NLS+/fgf13a:EGFP+ cells for WT and nr2f1a-/-;isl1-/- genotypes
* Fig 5 C-D: Number of myl7:DsRed2-NLS+/tcf21:NLS-GFP+ cells in WT versus nr2f1a-/-;isl1-/-
* Fig 6: Fraction or number of myl7:CSY cells that are Myh6-
* Fig 9: Frequency of tcf21:GFP in myocardial wall of nr2f1a-/-;isl1-/- hearts with and without tcf21 KD, either # cells or fraction of perimeter, as well as size of "gaps" in myh6 with and without tcf21 KD
* Fig 10: Frequency of tcf21:GFP in myocardial wall of nr2f1a-/-;isl1-/- hearts with and without tbx18 KD, either # cells or fraction of perimeter, as well as size of "gaps" in myh6 with and without tbx18 KD

© 2026. Published by The Company of Biologists under the terms of the Creative Commons Attribution License (https://creativecommons.org/licenses/by/4.0/).

Discussion:
9) Lines 401-403: "It is feasible that the reduced number of ACs in nr2f1a;isl1 mutants compared to nr2f1a mutants via conversion to ECs and exit from the atrial wall could be contributing to the greater deficit of ACs in these hearts." Is it possible to estimate the number of ACs converting to ECs? Is it consistent with the small (insignificant) discrepancy in total CM number in Fig. S1F? If the EC conversion rate is greater than expected, does this suggest an additional mechanism to maintain total CM number by proliferation or transdifferentiation?

Reviewer 2

Advance summary and potential significance to field

The report by Martin and colleagues entitled "Nr2f1a and Isl1 repress acquisition of epicardial identity in venous atrial cardiomyocytes" is a careful and detailed analysis of TF cooperation during early cardiac lineage specification. By comparing single nr2f1a and isl1 to nr2f1a;isl1 double mutant zebrafish embryos the study finds that loss of Isl1, in addition to Nr2f1a, exacerbates the loss of atrial cardiomyocyte (AC) identify and total AC numbers.
nr2f1a;isl1 double mutant embryos further do not generate any pacemaker cardiomyocytes (PCs), reaffirming Isl1 role in PC specification. Interestingly, the loss of AC identify in nr2f1a;isl1 double mutant hearts results in a transdifferentiation of ACs to epicardial cells (ECs), and with that a continued loss of AC numbers. Lastly, the study demonstrates that the EC marker genes tcf21and tbx18, but not wt1a or wt1b, are required for the transdifferentiation from ACs to ECs in nr2f1a;isl1 double mutant hearts.
The manuscript is very well written, and the study well rationalized. The data support the conclusions made by the authors, and all data are well presented and described. The work is done rigorously, and includes extensive experimentation with many different and appropriate genetic models. The complementary single cell transcriptomic analysis is a strengths of the work, and nicely supports the conclusions obtained via marker analysis, but also adds interesting new mechanistic depth to the work. Most importantly, the finding that loss of both Isl1 and Nr2f1a leads to transdifferentiation from ACs to ECs is interesting, and adds important insight to our understanding of the roles of these key TFs in critical steps of early lineage formation in the heart. Some minor suggestions for improvement of the manuscript are listed below.

Comments for the author

1. Quantification of the data: The data and conclusions of the study are convincing, and continuously build over the course of the story in a consistent manner. Never the less, the work lacks quantification of some of the study's key findings, especially given the high number of emrbyos that have been analyzed according to the figure legend information. Examples are the number of ECs in WT and double mutants (Figure S4/5); ECs that might be derived from ACs (Figure 5), lineage-traced Myh6+ and Myh6- cells (Figure 6), and all of the experiments assessing the role of various candidates in EC formation (Figures 9/10 and supplementary). For some of these observations, the paper only illustrates as little as one cell, and while very exciting, an understanding of the frequency of these observations seems appropriate, at least for some of these findings.

2. Transdifferentiation from AC to EC: The transdifferentiation mechanism in the double mutants is an exciting finding, providing insight into plasticity and fate mechanisms regulated by the nr2f1a and isl1 regulatory networks respectively.
2.1 Both the single mutants have been extensively studied - if the study could confirm, either by reference to these previous studies, or by complementary experiments, that this mechanism is indeed unique to the double mutant, it would help to clearly understand the individual roles of the TFs, and potentially their cooperative actions.
2.2 Lineage-tracing is powerful and rigorous approach to assess the transdifferentiation of ACs in the mutant hearts. While likely beyond the scope of this study, it could potentially be complemented with live imaging of the relevant reporters to strengthen the claim, and more importantly, to gain an understanding of the migratory behavior of the transdifferentiating cells

© 2026. Published by The Company of Biologists under the terms of the Creative Commons Attribution License (https://creativecommons.org/licenses/by/4.0/).

and their exit from the atria, as well as the temporal sequence of the mechanism (see below for more details on that).
2.3 It appears that only select ACs are undergoing this transdifferentiation, and from the data provided it does not appear like there is a specific localization to the transdifferentiating cells. Given that Nr2f1a and Isl1 are both broadly expressed, can the authors comments, or speculate (maybe in the discussion) on why this only happens in a subset of cells?

3. Transcriptomic analysis: The study concludes with a careful transcriptomic analysis on a single cell level across all 4 relevant genotypes, which represents a highly informative approach to dissecting the molecular mechanism. The data nicely support the observations earlier in the study (fewer ACs in the nr2f1a and nr2f1a;isl1 mutants, and a loss of AC identity).It also uncovers new and interesting concepts, such as for example the fact that ECs in double mutants also change their transcriptomic identity, loose some EC markers expression and in turn have higher expression of myocardial genes. This points to interesting roles for both Nr2f1a and Isl1 in general AC and EC fate specification and/or fate maintenance, and the data set can likely be further explored along these lines in the future.
3.1 The manuscript doesn't state how the AC and EC clusters were identified, eg what markers were used to assign AC/EC fate in the original UMPA (Figire 7A). These would be informative to show, in addition to the information in Figure S7A. Similarly, based on the dotted line in Figure 7A, the subsequent analysis excludes the AVC cluster, which might be interesting to consider in the analysis, at least in a separate set of analysis.
3.2 Information of how many embryos were used per genotype for the analysis needs to be added - either to the text, figure legend or methods section.

4. EC phenotype: There seem to be many more ECs in the nr2f1a;isl1 mutant hearts, according to several of the images presented. Based on the findings this could be attributed to the transfifferentiaiton from ACs. However, the extent of EC number seems rather high, and poses the question if it can be excluded that other mechanisms lead to the large number of ECs. Did the authors assess if ECs in nr2f1a;isl1 mutant hearts have a higher proliferative rate (the transcriptomic data could potentially be queried to get a sense for this as well), or is there another possible reason for this phenomenon.

5. AC fate maintenance versus specification and plasticity: The study states in various places that Nr2f1a and Isl1 are involved in maintenance of atrial identity. The discussion also speculates that based on the early expression of Isl1, these TFs are likely required during early specification, and the transdifferentiation is a consequence of these early events. The question of when Nr2f1a and Isl1 are playing key roles is both interesting and challenging to address experimentally without inducible KD experiments, which is beyond scope. In the studies approach, the latest time point assessed is 120hpf, by which the atria has become very small. If Nr2f1a and Isl1 are needed for AC maintenance over early specification, it would be interesting to know if this a progressive phenotype, and (if embryos are viable long enough) if the entire atria eventually transdifferentiates to ECs. Answering this could begin to point to highly specific roles of Nr2f1a and Isl1 during specific time points of early heart development. Another way of getting at these questions would be to perform transcriptomic analysis at multiple time points, in future studies.

Minor:

Fig. 1L: since the study is interested in comparing single nr2f1a mutants with nr2f1a;isl1 double mutants, it would be helpful to have both of the single mutants quantified for the different CM populations (even if that has been done in previous studies).

Fig. S12: the images of Myh7 and Tcf21EGFP in both wt and nr2f1a;isl1 double mutants are shown in the wrong order I believe (C'' and C''', and F'' anf F''').

First revision

© 2026. Published by The Company of Biologists under the terms of the Creative Commons Attribution License (https://creativecommons.org/licenses/by/4.0/).

Author response to reviewers' comments

We appreciate the positive assessments and thoughtful comments of the initial manuscript from both reviewers. While both reviewers praised the value of the study and indicated it provides a worthwhile addition to our understanding of transcription factor regulatory networks in heart development, they suggested additional analysis that could strengthen the manuscript. We have directly addressed all the comments and the additional analysis requested from both reviewers. The additional analysis we have performed and revisions to the text has helped to strengthen the experiments and clarify points in the manuscript. Our point-by-point responses to the reviewers' comments are below the original comments in blue. Changes made in the revised manuscript in response to the comments are highlighted in gray.

Comments from the Reviewers:
Reviewer 1: SUMMARY OF THE ADVANCE MADE IN THIS PAPER AND ITS POTENTIAL SIGNIFICANCE TO THE FIELD

In this manuscript, Martin et al. explore the interaction between Nr2f1a and Isl1 in the differentiation of different cell types in the developing zebrafish atrium. They find that Nr2f1a and Isl1 work together to maintain atrial myocardial identity in two stages, first against an alternative differentiation pathway to ventricular and atrioventricular canal cardiomyocytes at the atrial outflow and then against transdifferentiation to epicardial cells at the venous pole. This is a valuable addition to the understanding of gene regulatory networks involved in atrial chamber identity during zebrafish heart development. I recommend publication in Development with minor revisions mainly involving additional analyses.

SUGGESTIONS TO AUTHORS

Fig 1.

1) The partitioning of cells into VC, AVC, and AC identities would be clearer if the number and fraction of each of Myh6+/Myh7+, Myh6+/Myh7-, Myh6-/Myh7+ were simply shown for each of WT, nr2f1a-/- and nr2f1a-/-;isl1-/- on a single panel in Fig. 1. Currently Myh6+/Myh7+ are counted both in Myh6+ and Myh7+ populations, and one needs to look at many different panels to understand how the partitioning of cells changes across the genotypes. I currently see the expansion of AVC identity into the atrium in nr2f1a-/-;isl1-/- vs nr2f1a-/-, but I do not see the support for VC identity also increasing in nr2f1a-/-;isl1-/- vs nr2f1a-/-.

We apologize for the confusion. We used the different graphs to try to clearly describe and display what we observe with the cardiomyocyte identities at the two stages. The different breakdowns of Myh6+ and Myh7+ were presented in Fig. S1 of the original manuscript. To try to clarify the presentation of the cardiomyocyte populations for the reader, we have moved most of the graphs depicting the populations for Fig. S1 into Fig. 1 of the revised manuscript. The graph showing the overall quantification of Myh6 and Myh7 populations is now indicated in Fig. 1P. The graph showing Myh7+-only and total Myh7+ cardiomyocytes are presented in Figs. 1N and 1O, respectively.

In addition, the comment seems to possibly indicate we are double counting cells. To be clear, we are not double counting cells with respect to the total cardiomyocyte populations (Fig. 1P). However, double positive cardiomyocytes (atrioventricular canal cardiomyocytes) are included when referring to total Myh6 and Myh7 populations and displayed in these graphs.

We have modified the text describing these results to try to clarify these points (lines 140-161 of the revised manuscript).

2) The difference in AVC extent between the nr2f1a -/- and nr2f1a-/-;isl1-/- genotypes is not entirely convincing in the representative images F,G,J,K due to variations in morphology and positioning of the heart. Would they be more apparent if separate images were shown for each channel? Could a quantitative analysis be performed for each genotype describing the average Myh6 and Myh7 intensities as a function of distance from the venous pole?

© 2026. Published by The Company of Biologists under the terms of the Creative Commons Attribution License (https://creativecommons.org/licenses/by/4.0/).

We apologize that the expanded AVC of *nr2f1a* and *nr2f1a;isl1* mutants may not have been clear in the original images in Fig. 1. To better illustrate the AVC (Myh6+/Myh7+ cardiomyocytes), we have changed the images in Fig. 1J and 1G and have provided the individual channels in a new Fig. S1 of the revised manuscript.

We appreciate the suggestion to quantify the overlap of Myh6 and Myh7-expression cardiomyocytes using an additional complementary method. However, we are wary of quantification of expression intensity as a function of distance from the venous pole. As Reviewer #1 indicates and is depicted in Fig. 1D-K, there can be significant variability in heart and chamber morphologies, which would make this an unreliable indication of cell number. Thus, we use a nuclear marker to quantify cardiomyocytes because it allows for quantification independent of differences in heart morphology.

3) How are Myh6+ and Myh7+ calls determined? Is the proportion of cells binned into each of Myh6+/Myh7+, Myh6+/Myh7-, Myh6-/Myh7+ sensitive to the cutoffs chosen for each marker? In other words, are expression differences for each cell type binary (unambiguous positive or negative) or quantitatively graded and is this consistent across all genotypes?

As described in the Methods (lines 553-562) as well as our previous publications, such as Martin et al, 2023, we use the *myl7*:DsRed-NLS transgenic line to count the Myh6+ and Myh7+ cardiomyocytes. ImageJ is used to manually quantify the cardiomyocytes from the max intensity projects. The quantification is not automated. While we have explored the use of automatic quantification, these tools have not been able to reliably pick out nuclei if there is minimal background and required manual inspection and correction anyway. Hence, the different cardiomyocyte categories are not binned via an automated software tool.

Regarding the cutoff of expression, Myh6 and Myh7 are expressed around the membrane of cardiomyocytes. Even though expression can vary in the cardiomyocytes and each can be reduced in the expanded AVC of *nr2f1a* and *nr2f1a;isl1* mutants, Myh6+ and Myh7+ cells can be clearly distinguished from Myh6- and Myh7- cardiomyocytes. This can be seen in the new Fig. S1 as well as previous publications, such as Martin et al, 2023.

Fig. 2:

4) The Myh6+ status of fgf13a:GFP+ cells in the two groups of double mutant embryos in Fig 2H and Fig S2 is not clear. Are there any fgf13a:GFP+/Myh6+ cells at all in either group as there are in WT and nr2f1a-/- single mutants? If so, how can we be certain that these are not pacemaker cells? The functional data seems insufficient in Fig 3 and S3 - disruption in heart rate and conduction path could happen without complete depletion of pacemaker cells.

As shown in the Fig. 2H and Fig. S2 (Fig. S3 of the revised manuscript), while there appears to be *fgf13a*:EGFP+ cells in the diminutive atria of *nr2f1a;isl1* mutants by 96 hpf, these cells do not look similar to those in WT or *nr2f1a* mutants. The differences between the *fgf13a*:EGFP+ cells in the WT, *nr2f1a*, and *nr2f1a*;*isl1* mutant hearts can best be seen in the higher magnification optical sections of Fig. 4. In WT hearts, the *fgf13a*:EGFP+/Myh6+ cells are restricted to the venous pole, consistent with being PCs. In the *nr2f1a* mutants, the *fgf13a*:EGFP+/Myh6+ cells are continuous from the venous pole up to the Myh6+/Myh7+ AVC cardiomyocytes, consistent with the expansion of PC identity (Martin et al, 2023). However, in the *nr2f1a*;*isl* mutants, we observe there are *fgf13a*:EGFP+/Myh6+ cells that appear by 96 hpf sporadically within the diminished venous pole, as well as *fgf13a*:EGFP+/Myh6- cells within the myocardial wall. We realize that we did not clearly distinguish the *fgf13a*:EGFP populations in the initial manuscript. We have revised the description in the revised manuscript to better articulate the phenotypic variability and different *fgf13a*:EGFP+ populations we observe within the small atria of the *nr2f1a*;*isl1* mutant hearts (lines 167-181 and lines 216-222 of the revised manuscript). Please also see our responses to the comments about Fig. 5, Fig. 9 and Fig. 10, where we quantified cells.

The conclusion that *fgf13a*:EGFP is not marking sinoatrial PCs in *nr2f1a;isl1* mutants is not solely based on the functional defects. In contrast to the *nr2f1a* mutant hearts, the *nr2f1a*;*isl1* mutants lack *isl1* expression in the hearts, which is currently the most definitive marker of pacemaker cardiomyocytes available in zebrafish (Tessadori et al., 2012). Our single cell data show that *isl1* as

© 2026. Published by The Company of Biologists under the terms of the Creative Commons Attribution License (https://creativecommons.org/licenses/by/4.0/).

well as *shox2*, another PC marker, are lost in the *nr2f1a;isl1* cardiomyocytes, but not the *nr2f1a* mutants in the single cell data (Fig. S8C,D). Note that we also examined other reported PC markers, including *hcn4* and *tbx3a*. However, *hcn4* was not specific to the PC population and *tbx3a* expression was below detection. Thus, they weren't informative and not included in the manuscript. Additionally, we show that Nkx2.5, which is expressed in working cardiomyocytes and excluded from PCs, is expressed throughout the diminutive atrium of the *nr2f1a*;*isl1* mutants (Fig. S4). As we showed previously, Nkx2.5 expression is lost in atrial cardiomyocytes of *nr2f1a* single mutants that are transdifferentiating into PCs (Martin et al, 2023).

Regarding the interpretations of the functional analysis, the arrythmias (irregular beading and long lapses in contraction) of the *nr2f1a*;*isl* mutant hearts recapitulates those in the published *isl1* mutants (de Pater et al, 2009) and our newly generated allele. Moreover, if there was just a reduction in sinoatrial PCs, we would expect that the hearts would have reduced hearts rates, similar to what has been reported in Ren et al, 2019 and likely not irregular beating patterns as observed in the *isl1* and *nr2f1a*;*isl1* mutants.

Thus, our interpretations are based on the combination of functional and expression data of currently available PC markers in zebrafish, which together are consistent with *nr2f1a*;*isl1* lacking sinoatrial PCs.

Fig. 3:

5) Was motion correction performed for analysis of GCaMP imaging? Alternatively, was analysis done to demonstrate that motion is negligible in the DF/F traces (e.g. by choosing several regions of interest of different sizes and positions within the same sample and showing that the traces are identical)? If not, the durations and shapes of Ca2+ transients are not interpretable and the authors should clearly note this (the experiment is still useful for comparing heart rate and heart rate variability).

We did not perform motion correction because we were imaging the hearts in live embryos and it was not necessary to examine the cardiac contractions and arrhythmias. We performed the imaging this way because we were not able to completely stop cardiac contractions nor did we use isolated hearts, using a variety of methods that have been reported previously (Chow et al., 2022; Jou et al., 2010; Marchant et al., 2022; Salgado-Almario et al., 2022). Thus, we are cognizant of these issues and were careful not make interpretations about conduction properties within the hearts with the gCamp imaging. The traces of the calcium transients were used to compare and present the heart rates and variability of contractions in the *nr2f1a*;*isl1* mutants. We have modified the statement in the Methods (line 635).

6) I suggest the term "intensity trace" as opposed to "kymograph" for the legend of plots B, D, F, H to avoid confusion. Kymographs in modern signal processing commonly refer to a plot with time on one axis, a spatial coordinate on another, and a quantity such as intensity plotted as color (as opposed to the traces generated by an analog "kymograph" instrument).

We have changed the term to “intensity traces.”

7) A spatial description of GCaMP activity should be shown (e.g. a modern kymograph, or an activation map) to illustrate what propagation looks like from the primary and secondary pacemakers and support the claim in the text of propagation from AV node. Quantification of the frequency of these events versus normal propagation should be done.

We agree that additional data supporting this statement should have been provided. However, since this data does not really add to the primary focus of the manuscript, we have removed the statement.

Figs. 4-10

8) Representative images are generally convincing but quantification should be done to support the biological claims, for example:

© 2026. Published by The Company of Biologists under the terms of the Creative Commons Attribution License (https://creativecommons.org/licenses/by/4.0/).

* Fig 4: fraction of fgf13a:GFP cells that are Myh6+ in WT, nr2f1a-/-, and nr2f1a-/-;isl1-/- genotypes

Unfortunately, we cannot accurately quantify the *fgf13a*:GFP cells for this set of experiments because we did not use a nuclear or cell membrane marker to label individual cardiomyocytes. However, as indicated in our response to the comment above regarding Fig. 2 and Fig. 2H, it is also not clear that blind quantification of the *fgf13a*:GFP+/Myh6+ cells in WT, *nr2f1a* mutant, and *nr2f1a*;*isl1* mutants would be informative for interpretation of the phenotype, as *fgf13a*:GFP+/Myh6+ cells represent different types of cells within the hearts. Again, we only observe *fgf13a*:GFP+/Myh6+ cells sporadically within the atria of *nr2f1a*;*isl1* mutants. In *nr2f1a* mutants, the ectopic *fgf13a*:EGFP expression expands from the venous pole of the atrium. In the *nr2f1a*;*isl1* mutants, they initially do not have *fgf13a*:EGFP within the heart at 48 hpf, similar to the *isl1* mutants (Fig. 2G). *Fgf13a*:EGFP then becomes expressed later sporadically in Myh6+ atrial myocardium and AV node cells by 96 hpf. Additionally, while *fgf13a*:EGFP can mark epicardial cells (ECs), we never observe epicardial cells on the atria of WT or *nr2f1a* mutant hearts at 96 hpf. We have not observed atrial epicardial cells in WT embryos until ~144 hpf. While we cannot accurately quantify these *fgf13a*:EGFP cells from this particular experiment, we have tried to better describe the different *fgf13a*:EGFP populations observed within the heart, as indicated in the response regarding Figs. 2 and 2H above (lines 166-180 and lines 215-221 of the revised manuscript), and have quantified these cells for experiments described in Fig. 5 (see below) in the revised manuscript.

* Fig 5 A-B: Myh6 intensity in myl7:DsRed2-NLS+/fgf13a:EGFP+ cells for WT and nr2f1a-/-;isl1-/- genotypes

We realize that based on our qualitative statement in the initial manuscript of the Myh6+/fgf13a:EGFP+/myl7:DsRed2-NLS+ cells in the *nr2f1a*;*isl1* mutants, it would justify quantification. However, the cell morphology differences between the atrial cardiomyocytes coupled with not using a membrane marker in the WT and *nr2f1a*;*isl1* mutants makes the comparison difficult to assess, as well determine what should be compared between the cells.

To assess the phenotype in the revised manuscript, instead we have quantified the number of distinct, non-AV node, sporadic Myh6+/*fgf13a*:EGFP+/*myl7*:DsRed-NLS+ cells and Myh6-/fgf13a:EGFP+/myl7:DsRed-NLS+ cell in the atria of WT and *nr2f1a;isl1* hearts. While neither of these cell types are observed in WT hearts, we observed Myh6+/*fgf13a*:EGFP+/*myl7*:DsRed-NLS+ and Myh6-/*fgf13a*:EGFP+/*myl7*:DsRed-NLS+ cells in the *nr2f1a*;*isl1* mutant hearts. Although there may be relatively few of these cells in the diminutive hearts, they are observed in the majority of *nr2f1a*;*isl1* mutant hearts. The appearance of Myh6-/*fgf13a*:EGFP+ cells in the myocardial wall is consistent with the hypothesis that atrial cardiomyocytes are losing Myh6+ expression and gaining *fgf13a*:EGFP expression. We apologize that the description of these cell populations within the *nr2f1a*;*isl1* mutant hearts was deficient in the initial manuscript. We have modified the text in the results section (lines 229-240) and added additional images and the quantification of cells to Fig. 5 of the revised manuscript.

* Fig 5 C-D: Number of myl7:DsRed2-NLS+/tcf21:NLS-GFP+ cells in WT versus nr2f1a-/-;isl1-/-

The number of co-labeled *myl7*:DsRed2-NLS+/*tcf21*:NLS-GFP+ cells we observed per embryo was small, but comparable to the number of Myh6-/*fgf13a*:EGFP+/*myl7*:DsRed-NLS+. While there was not a statistical difference in the number *myl7*:DsRed2-NLS+/*tcf21*:NLS-GFP+ cells between WT and *nr2f1a*;*isl1* mutant hearts, we never observed co-labeled cells in WT embryos and observed at least 1 co-labeled cell in the majority of *nr2f1a*;*isl1* mutant hearts examined. We have revised the text in the Results section (lines 240-250) and moved the images and added the quantitative data to a new Fig. 6 of the revised manuscript.

* Fig 6: Fraction or number of myl7:CSY cells that are Myh6-

While we recognize that quantification of the number of *myl7*:CSY+/Myh6- cells would be ideal, similar to our experiment for Fig. 4, we cannot accurately quantify the number of *myl7*:CSY+/Myh6- cells from these lineage-tracing experiments as we did not use a nuclear or membrane marker. Additionally, we have not assessed the efficiency of recombination. However, we do not think that

© 2026. Published by The Company of Biologists under the terms of the Creative Commons Attribution License (https://creativecommons.org/licenses/by/4.0/).

lack of quantification of *myl7*:CSY+/Myh6- cells precludes interpretation from the lineage-tracing experiment that atrial cardiomyocytes in the *nr2f1a*;*isl1* mutant hearts lose Myh6 expression, complementing the analysis in Fig. 4, Fig. 5, and Fig. 6 (of the revised manuscript).

* Fig 9: Frequency of tcf21:GFP in myocardial wall of nr2f1a-/-;isl1-/- hearts with and without tcf21 KD, either # cells or fraction of perimeter, as well as size of "gaps" in myh6 with and without tcf21 KD

We have quantified the *tcf21*:EGFP+ cells in the myocardial wall from the *nr2f1a*;*isl1* mutant hearts with and without tcf21 KD. However, measuring the size of the gaps is not the best indicator of rescue or informative as their size can be highly variable and dependent on the heart morphology. Therefore, we quantified the number of *tcf21*:EGFP+ gaps in the myocardial layer of *nr2f1a*;*isl1* mutant hearts with and without *tcf21* KD hearts. While this assessment also does not indicate cell number within the gaps, it showed that all gaps contain *tcf21*:EGFP+ cells in the control *nr2f1a*;*isl1* mutant hearts, while there were no or very few *tcf21*:EGFP+ cells in the myocardial gap in *nr2f1a*;*isl1* mutant hearts with *tcf21* KD. This is now mentioned in the text of the results (lines 363-366) and presented in Fig. 10 of the revised manuscript.

* Fig 10: Frequency of tcf21:GFP in myocardial wall of nr2f1a-/-;isl1-/- hearts with and without tbx18 KD, either # cells or fraction of perimeter, as well as size of "gaps" in myh6 with and without tbx18 KD

For the *tbx18* KD experiments, we have performed the same quantification of *tcf21*:EGFP+ cells and the number of gaps in the myocardial walls of *nr2f1a*;*isl1* mutant hearts as for *tcf21* KD. The quantification showed there were *tcf21*:EGFP+ cells in the myocardial wall of control *nr2f1a*;*isl1* mutant hearts, while there were fewer *tcf21*:EGFP+ cells and increased number of Myh6+ gaps in the myocardial walls of *nr2f1a*; *isl1* mutant hearts with *tcf18* KD. The quantification is now mentioned in the revised test of the results (lines 379-381) and presented in Fig. 11 of the revised manuscript.

Discussion:
9) Lines 401-403: "It is feasible that the reduced number of ACs in nr2f1a;isl1 mutants compared to nr2f1a mutants via conversion to ECs and exit from the atrial wall could be contributing to the greater deficit of ACs in these hearts." Is it possible to estimate the number of ACs converting to ECs? Is it consistent with the small (insignificant) discrepancy in total CM number in Fig. S1F? If the EC conversion rate is greater than expected, does this suggest an additional mechanism to maintain total CM number by proliferation or transdifferentiation?

We observe a deficit of ~17 atrial (Myh6+/Myh7-) cardiomyocytes from 48 to 96 hpf in the *nr2f1a*;*isl1* mutant hearts. The most *tcf21*:NLS-EGFP+ cells that we have observed at a given time is ~6. As this alone cannot explain the deficit, we hypothesize that the greater deficit in the *nr2f1a*;*isl1* mutants compared to the *nr2f1a* mutants, is due to two factors: 1) ACs (Myh6+) cells progressively transdifferentiating to ventricular (Myh7+) cells via transitioning from Myh6+/Myh7+ AVCs. While this is similar to *nr2f1a*, this phenotype and the extent of conversion to ventricular cardiomyocytes is exacerbated in the *nr2f1a*;*isl1* mutants; 2) The loss of ACs via conversion to ECs and their exit from the myocardial layer. Additionally, although not presented in the manuscript, we have not observed differences in proliferation in the *nr2f1a*;*isl1* mutant hearts compared to WT siblings. Thus, we do not think the conversion rate is greater than expected and the deficit is from a progressive loss of atrial cells via two mechanisms. We have modified the Discussion (lines 439-441) in the revised manuscript to better emphasize these points.

Reviewer 2: SUMMARY OF THE ADVANCE MADE IN THIS PAPER AND ITS POTENTIAL SIGNIFICANCE TO THE FIELD

The report by Martin and colleagues entitled "Nr2f1a and Isl1 repress acquisition of epicardial identity in venous atrial cardiomyocytes" is a careful and detailed analysis of TF cooperation during early cardiac lineage specification. By comparing single nr2f1a and isl1 to nr2f1a;isl1 double mutant zebrafish embryos the study finds that loss of Isl1, in addition to Nr2f1a, exacerbates the loss of atrial cardiomyocyte (AC) identify and total AC numbers.

© 2026. Published by The Company of Biologists under the terms of the Creative Commons Attribution License (https://creativecommons.org/licenses/by/4.0/).

nr2f1a;isl1 double mutant embryos further do not generate any pacemaker cardiomyocytes (PCs), reaffirming Isl1 role in PC specification. Interestingly, the loss of AC identify in nr2f1a;isl1 double mutant hearts results in a transdifferentiation of ACs to epicardial cells (ECs), and with that a continued loss of AC numbers. Lastly, the study demonstrates that the EC marker genes tcf21and tbx18, but not wt1a or wt1b, are required for the transdifferentiation from ACs to ECs in nr2f1a;isl1 double mutant hearts.
The manuscript is very well written, and the study well rationalized. The data support the conclusions made by the authors, and all data are well presented and described. The work is done rigorously, and includes extensive experimentation with many different and appropriate genetic models. The complementary single cell transcriptomic analysis is a strengths of the work, and nicely supports the conclusions obtained via marker analysis, but also adds interesting new mechanistic depth to the work. Most importantly, the finding that loss of both Isl1 and Nr2f1a leads to transdifferentiation from ACs to ECs is interesting, and adds important insight to our understanding of the roles of these key TFs in critical steps of early lineage formation in the heart.

Some minor suggestions for improvement of the manuscript are listed below.

SUGGESTIONS TO AUTHORS

1. Quantification of the data: The data and conclusions of the study are convincing, and continuously build over the course of the story in a consistent manner. Never the less, the work lacks quantification of some of the study's key findings, especially given the high number of emrbyos that have been analyzed according to the figure legend information. Examples are the number of ECs in WT and double mutants (Figure S4/5); ECs that might be derived from ACs (Figure 5), lineage-traced Myh6+ and Myh6- cells (Figure 6), and all of the experiments assessing the role of various candidates in EC formation (Figures 9/10 and supplementary). For some of these observations, the paper only illustrates as little as one cell, and while very exciting, an understanding of the frequency of these observations seems appropriate, at least for some of these findings.

Thank you for these suggestions to help strengthen our experiments. This point is similar to many that Reviewer #1 brought up. We have tried to provide quantification of the data wherever feasible that will provide greater insights into interpretation of the experiments. In the revised manuscript, we have provided quantification of cells for Fig. S5, Fig. 5, Fig. 9, and Fig. 10. This data is described in the text (lines 209-212, 229-250, 363-366, and 379-381) and provided in Fig. S6 (formerly Fig. S5), Fig. 5 (now separated into Fig. 5 and Fig. 6), Fig. 10 (formerly Fig. 9), and Fig. 11(formerly Fig. 10) of the revised manuscript. While in some of the analysis we on average only observe a single cell in the diminutive atrium, they are observed in the majority of embryos examined.

We did not provide quantification for Fig. S5 (formerly Fig. S4), as it will not change the interpretations of the experiments that *fgf13a*:EGFP marks ECs in WT and *nr2f1a*;*isl1* mutants.

2. Transdifferentiation from AC to EC: The transdifferentiation mechanism in the double mutants is an exciting finding, providing insight into plasticity and fate mechanisms regulated by the nr2f1a and isl1 regulatory networks respectively.

2.1 Both the single mutants have been extensively studied - if the study could confirm, either by reference to these previous studies, or by complementary experiments, that this mechanism is indeed unique to the double mutant, it would help to clearly understand the individual roles of the TFs, and potentially their cooperative actions.

We have not observed AC to EC transdifferentiation in either of the *nr2f1a* or *isl1* mutants alone. We have revised the text to clearly highlight the individual vs cooperative actions of these transcription factors (lines 407-415).

2.2 Lineage-tracing is powerful and rigorous approach to assess the transdifferentiation of ACs in the mutant hearts. While likely beyond the scope of this study, it could potentially be complemented with live imaging of the relevant reporters to strengthen the claim, and more importantly, to gain an understanding of the migratory behavior of the transdifferentiating cells

© 2026. Published by The Company of Biologists under the terms of the Creative Commons Attribution License (https://creativecommons.org/licenses/by/4.0/).

and their exit from the atria, as well as the temporal sequence of the mechanism (see below for more details on that).

Thank you for this suggestion. While we recognize that time-lapse imaging would be ideal and it would be exciting to observe transdifferentiating cells in live embryos, we have not yet been able to perform the potentially long time-lapse imaging to catch these events in the present study. Thus, we agree this experiment is beyond the scope of the present study.

2.3 It appears that only select ACs are undergoing this transdifferentiation, and from the data provided it does not appear like there is a specific localization to the transdifferentiating cells. Given that Nr2f1a and Isl1 are both broadly expressed, can the authors comments, or speculate (maybe in the discussion) on why this only happens in a subset of cells?

We agree that our data support some atrial cardiomyocytes (Myh6+/Myh7- cells) are undergoing transdifferentiation from ACs to ECs. Presently, the transdifferentiating cells would appear to be stochastic, as there is not a clear pattern or anatomical position that this appears to be occurring in the small atria of *nr2f1a;isl1* mutant hearts. Thus, we presently do not have a good answer as to why it happens in a subset of atrial cardiomyocytes. As is pointed out by Reviewer #2, it is not clear the protein localization, at least at the time when we observe the transition, could explain this observation. Nr2f1a is expressed throughout the atrial cardiomyocytes (Duong et al, 2018), while Isl1 is restricted to the venous pole (Ren et al., 2019; Tessadori et al, 2012). However, we previously reported that induction of Nkx2.5 is sufficient to repress PC identity in *nr2f1a* mutant atria, suggesting that the major requirements of Nr2f1a in maintenance of identity may actually be established during cardiac differentiation. We have added this point to the Discussion (lines 407-415).

3. Transcriptomic analysis: The study concludes with a careful transcriptomic analysis on a single cell level across all 4 relevant genotypes, which represents a highly informative approach to dissecting the molecular mechanism. The data nicely support the observations earlier in the study (fewer ACs in the nr2f1a and nr2f1a;isl1 mutants, and a loss of AC identity).It also uncovers new and interesting concepts, such as for example the fact that ECs in double mutants also change their transcriptomic identity, loose some EC markers expression and in turn have higher expression of myocardial genes. This points to interesting roles for both Nr2f1a and Isl1 in general AC and EC fate specification and/or fate maintenance, and the data set can likely be further explored along these lines in the future.

3.1 The manuscript doesn't state how the AC and EC clusters were identified, eg what markers were used to assign AC/EC fate in the original UMPA (Figire 7A). These would be informative to show, in addition to the information in Figure S7A. Similarly, based on the dotted line in Figure 7A, the subsequent analysis excludes the AVC cluster, which might be interesting to consider in the analysis, at least in a separate set of analysis.

To determine the identity of cell populations, we used manual curation for canonical markers established within the literature and comparison to those from our and others published single cell data sets. This is now mentioned in the text (lines 286-288) of the revised manuscript. The enriched genes beyond what is shown in the figures are included in Table S1. For ACs, these markers included *myh6 (amhc)*, *nr2f1a*, and *kcnj5*, with the first two markers being mentioned in the subclustering analysis. For epicardial cells, we used markers including *tcf21*, *tbx18*, *wt1a*, and *wt1b*, also a focus of the subclustering analysis. However, a significant description of the AC and ECs used for the original assignments seems a bit redundant as they overlap with the markers in the subclusters. Thus, without a way to succinctly present this in a non-redundant manner, we have not elaborated on this with respect to the original UMAP.

While we agree that examining the AVC would be interesting in the future. It was not selected for comparison because we do not observe putative transdifferentiation of AVCs to ECs in the AVC (Myh6+Myh7+) cardiomyocytes of the *nr2f1a;isl1* mutants. Thus, we focused on comparisons of the AC and ECs, since our data suggested these were the most relevant populations.

3.2 Information of how many embryos were used per genotype for the analysis needs to be added - either to the text, figure legend or methods section.

© 2026. Published by The Company of Biologists under the terms of the Creative Commons Attribution License (https://creativecommons.org/licenses/by/4.0/).

We have added the number of embryos used and hearts that were collected to ascertain the cells used for the single cell data to the Methods of the revised manuscript (lines 588-590 and 602-603).

4. EC phenotype: There seem to be many more ECs in the nr2f1a;isl1 mutant hearts, according to several of the images presented. Based on the findings this could be attributed to the transfifferentiaiton from ACs. However, the extent of EC number seems rather high, and poses the question if it can be excluded that other mechanisms lead to the large number of ECs. Did the authors assess if ECs in nr2f1a;isl1 mutant hearts have a higher proliferative rate (the transcriptomic data could potentially be queried to get a sense for this as well), or is there another possible reason for this phenomenon.

Based on our current data, we would not conclude that there are more ECs in *nr2f1a;isl1* mutants. As shown in Fig. S6 (formerly Fig. S5), *nr2f1a;isl1* mutants have ECs on the ventricle, though clearly fewer than WT sibling embryos, which as indicated above we have now quantified in the revised figure. Note that we did not count the putative ECs at the venous pole of the *nr2f1a;isl1* mutant hearts as the equivalent *tcf21*:NLS-EGFP cell type in WT heart cells is not clear.

We did assessed proliferation in the EC population from the transcriptomic data as suggested. There is not a difference between WT and *nr2f1a;isl1* mutant ECs based on a G2M phase module score in these cells. As this did not show a difference, we have not added it to the manuscript, though we are happy to provide this data if it is thought to be necessary.

5. AC fate maintenance versus specification and plasticity: The study states in various places that Nr2f1a and Isl1 are involved in maintenance of atrial identity. The discussion also speculates that based on the early expression of Isl1, these TFs are likely required during early specification, and the transdifferentiation is a consequence of these early events. The question of when Nr2f1a and Isl1 are playing key roles is both interesting and challenging to address experimentally without inducible KD experiments, which is beyond scope. In the studies approach, the latest time point assessed is 120hpf, by which the atria has become very small. If Nr2f1a and Isl1 are needed for AC maintenance over early specification, it would be interesting to know if this a progressive phenotype, and (if embryos are viable long enough) if the entire atria eventually transdifferentiates to ECs. Answering this could begin to point to highly specific roles of Nr2f1a and Isl1 during specific time points of early heart development. Another way of getting at these questions would be to perform transcriptomic analysis at multiple time points, in future studies.

Indeed, in *nr2f1a* mutants, it appears that all the Myh6+ (Myh7-) ACs will progressively transdifferentiate into PCs (Martin et al, 2023). However, 120 hpf is as far as we have taken the *nr2f1a;isl1* mutant embryos for analysis as their hearts become significantly distended. In addition, some embryos start dying and becoming necrotic past this stage. As indicated in the lineage tracing experiment at 120 hpf, we have not observed a complete transdifferentiation of remaining ACs into ECs.

Yes. We agree performing single cell transcriptomics over multiple time points in the future would be ideal and informative. It may be feasible now given the costs of sequencing have come down significantly.

Minor:

Fig. 1L: since the study is interested in comparing single nr2f1a mutants with nr2f1a;isl1 double mutants, it would be helpful to have both of the single mutants quantified for the different CM populations (even if that has been done in previous studies).

We compared *nr2f1a* mutants from previous work to the current work with *nr2f1a;isl1* double mutants because we were not able to make these comparisons within experiments from the same clutches. There is not a high enough recombination rate to garner *nr2f1a* mutants from crosses of *nr2f1a;isl1* heterozygous fish. The mean number of cardiomyocytes in all the conditions can vary a little between experiments due to a variety of reasons, which is why we make quantitative comparisons and perform statistical analysis between WT siblings and mutants within the same experimental replicate. Importantly, regardless of the quantification of cardiomyocytes, the trends

© 2026. Published by The Company of Biologists under the terms of the Creative Commons Attribution License (https://creativecommons.org/licenses/by/4.0/).

with respect to effect on cell number and proportions are consistent across replicates. Thus, using the proportions of cardiomyocytes is best for comparison between these different experiments.

Fig. S12: the images of Myh7 and Tcf21EGFP in both wt and nr2f1a;isl1 double mutants are shown in the wrong order I believe (C'' and C''', and F'' anf F''').

Thank you for catching this. It has been corrected.

Second decision letter

MS ID#: dev.205396R1

MS TITLE: Nr2f1a and Isl1 repress acquisition of epicardial identity in venous atrial cardiomyocytes

AUTHORS: Kendall E. Martin, Andrew T. Fernandes, Mohammed Sayed, Margaret A. Hanlon, Samuel Fallon, Hee-Woong Lim and Joshua Waxman

Dear Dr Waxman,

I am happy to tell you that your manuscript has been accepted for publication in Development, pending our standard publication integrity checks.

© 2026. Published by The Company of Biologists under the terms of the Creative Commons Attribution License (https://creativecommons.org/licenses/by/4.0/).
